# Block copolymer multistate material circuit

**DOI:** 10.1038/s41467-026-78278-2

**Published:** 2026-10-06

**Authors:** Hongxin Wei, Lingmin Lan, Li Luo, Ziqi Liu, Changxing Ji, Diyuan Zheng, Jie Chen, Robert J. Hickey, Chao Lang

**Affiliations:** 1 South China Advanced Institute for Soft Matter Science and Technology, State Key Laboratory of Advanced Papermaking and Paper-based Materials, School of Emergent Soft Matter, South China University of Technology, Guangzhou, China; 2 Guangdong Provincial Key Laboratory of Functional and Intelligent Hybrid Materials and Devices, Guangdong Basic Research Center of Excellence for Energy and Information Polymer Materials, South China University of Technology, Guangzhou, China; 3 Materials Science and Engineering, The Pennsylvania State University, University Park, PA USA; 4 Materials Research Institute, The Pennsylvania State University, University Park, PA USA

**Keywords:** Mechanical properties, Molecular self-assembly, Self-assembly

## Abstract

Nature maximizes efficiency and minimizes waste by using simple building blocks to create complex systems. Developing synthetic materials inspired by similar principles like cell multistability could enable sustainable material lifecycles and various applications. Here, we report a recyclable multistate material system with quantitatively defined states and circuit-like transition pathways, offering a series of switchable material properties by dual-phase crystalline control in self-assembled nanostructures. In state with high crystallinity and alignment, the material displays exceptional toughness (441 MJ m^−3^) higher than spider silk and true strength of 738 MPa, raising loads a million times its own weight. Fine-tuning deformation behaviors of rigid states provides excellent impact resistance (force damping up to 94%) outperforming UHMWPE and Kevlar. Rigid states can switch to soft states rapidly and reversibly, with modulus decreasing from 0.88 GPa to 0.1 MPa in seconds. These transitions also enable high-performance actuators for adaptive fabrics, biomedical devices, and micromotors.

## Abstract (web summary)

‘Here, the authors report a recyclable multistate material system with quantitatively defined states and circuit-like transition pathways, offering a series of switchable material properties by dual-phase crystalline control in self-assembled nanostructures.’

## Introduction

Nature relies on a limited set of substances to create materials with extraordinary diversity and functionality^1,2^. Through hierarchical structure control of building blocks such as proteins, polysaccharides, and minerals, nature engineers astonishingly diverse material properties tailored to specific functions^3–6^—from static features like the softness of cornea and the strength of bone, to dynamic behaviors like modulus switching in sea cucumbers and actuation of muscles. By operating in a closed-loop, self-sustaining system where resources are efficiently reused, it minimizes waste and reduces the need for excessive chemical complexity. A further demonstration of nature’s high efficiency and flexibility is multistability^7–9^, where cells differentiate into thousands of types and states relying on the same set of genes, dynamically changing cell functions and properties, adapting to the environment.

Developing nature-inspired multistate materials holds great potential for creating sustainable material production and usage cycles by reducing energy consumption and waste generation^10–13^. The adaptive properties of these materials, such as modulus changing^14–16^, actuation^17–21^, and programmability^22–24^, also create exciting opportunities for applications in biomedicals^25–28^, smart fabrics^29^, microelectromechanical systems (MEMS)^30,31^, robotics^32,33^, and related fields^34–37^. Although multistate systems have been successfully achieved through mechanical metamaterials^29,38,39^ (e.g., origami- and kirigami-inspired designs), creating single-material systems with multiple states that can be used stably at room temperature remains a significant challenge^15,16^. One promising route lies in crystalline block copolymers, where microphase-separated crystalline domains can be tuned in crystallinity and orientation as structural handles. While the crystallization behaviors and mechanisms of such systems, including double crystalline block copolymers, have been extensively studied, their states and transitions have not been systematically organized within a unified parameter space.

**Fig. 1:**
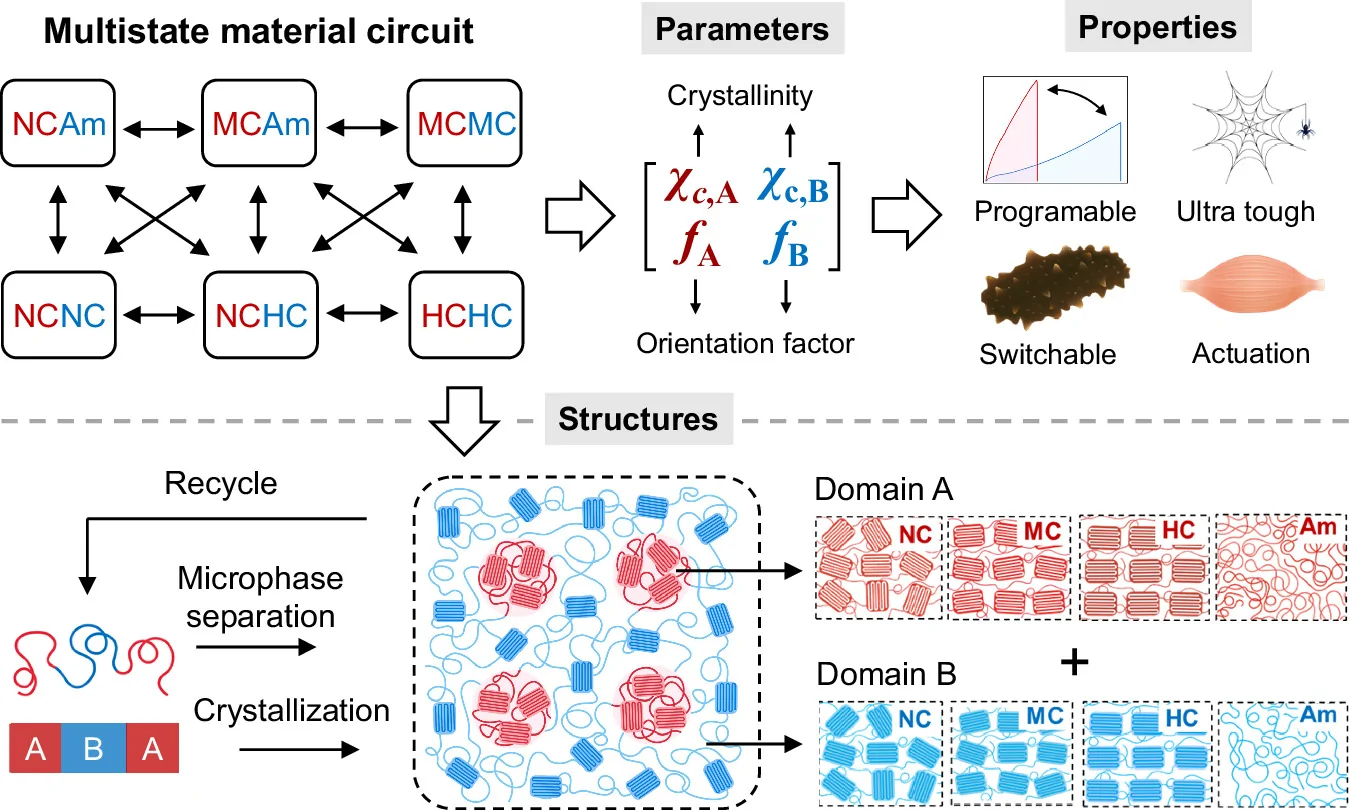
The MMC system was constructed from microphase-separated semi-crystalline A- and B-block domains connected by physical crosslinks. A 2 **×** 2 matrix consists of the degree of crystallinity (*χ*_*c*,A_ and *χ*_*c*,B_) and Hermans’ orientation factor (*f*_A_ and *f*_B_) of block A and block B was used to quantitatively describe the state of the system. To represent key states of the system, domains were categorized into 4 different types, *i.e*., Am, NC (NC’ for *χ*_*c*_ ≤ 10%), MC, and HC. The overall material state is defined as the domain state of the A-block followed by the B-block.

In this work, we report a multistate material circuit (MMC) system based on double-crystalline triblock copolymer, featuring a series of quantitatively defined states and a rich, circuit-like network of transitions (Fig. 1). Through separate control over microphase-separated A- and B-domains, each material state can be defined using a 2 × 2 matrix consisting of crystallinity (*χ*_*c*,A_ and *χ*_*c*,B_) and Hermans’ orientation factor (*f*_A_ and *f*_B_). Based on their effects on material properties and transition behaviors, the state of A- or B-block domains is classified as amorphous (Am), non-aligned crystalline (NC, *f* between 0 to −0.1), medium-aligned crystalline (MC, *f* between −0.1 to −0.35), and highly-aligned crystalline (HC, *f* between −0.35 to −0.5). The overall state of the material is then defined as the combination of A- and B-block domains.

We show that the MMC system provides on-demand control over a broad spectrum of material properties and functionalities, which can be achieved using the same polymer to reduce chemical complexity. Structure control alone—without chemical modification or additives — provided access to mechanical behaviors ranging from soft and deformable to strong and tough from a single composition. Using mild external stimuli such as hydration, heating, or straining, the system demonstrates both discrete (with a single end state) and continuous (involving different intermediate microstates) transitions, enabling versatile functionalities like dramatic modulus change reminiscent of sea cucumbers, muscle-like programmable actuation, and highly effective impact damping. The combination of continuous transitions and state quantification within a unified MMC framework allows on-demand control over material structure and properties. Since no chemical reactions or additives other than water were introduced, the system can be easily reset to the initial state or recycled to raw polymer, regardless of its current state.

## Results

### Material states, triggering methods, and transition pathways of MMC

We use polycaprolactone-*b*-poly(ethylene oxide)-*b*-polycaprolactone (C_400_O_1818_C_400_, Fig. S1 and Table S1) as a model polymer system to illustrate the construction of MMC. Although the crystallization behaviors of amorphous-crystalline^40,41^ or crystalline-crystalline block copolymers^42,43^ have been widely studied, their mechanical properties and responsive behaviors, such as modulus switching and actuation, have received far less attention. In this work, we focused on the self-assembled structure of disordered spheres with A-block being spherical micelles dispersed in the B-block matrix. With two end A-blocks of the same polymer chain spanning neighboring micelles to form a bridging configuration^44,45^, physical crosslinks were created to provide mechanical strength and recyclability for the polymer network^46,47^.

**Fig. 2:**
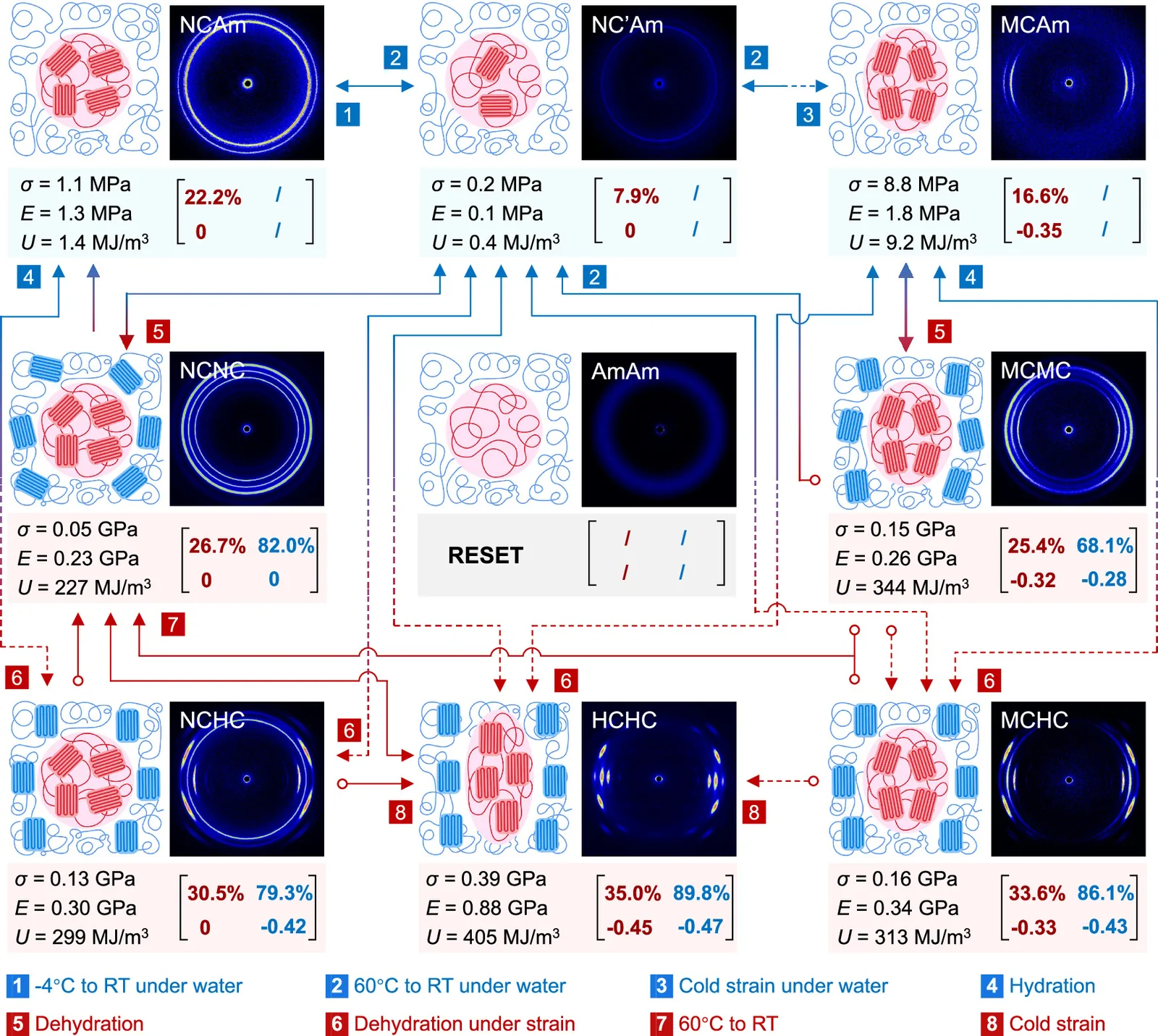
A circuit-like diagram maps key states, triggering methods, and transition pathways of the system. A broad range of mechanical properties at different states was achieved using the same polymer of C_400_O_1818_C_400_ (Fig. S1 and Table S1). Eight key states, including three hydrogel states (NCAm, NC’Am, and MCAm, blue-colored) and five dry states (NCNC, NCHC, MCMC, MCHC, and HCHC, red-colored) were listed based on material properties and transition behaviors (see more details in Table S2). Double-sided and one-sided arrow lines stand for reversible and irreversible transitions via labeled triggers, respectively. Dashed lines denote continuous transitions involving a series of intermediate states. All the states can be stably used at room temperature except for AmAm, which is used to reset the system. For each key state, the mechanical properties shown are representative of one of its microstates. The values were determined from engineering stress–strain curves and are reported as means of three independent measurements.

To fully elucidate the structure and transitions of MMC, its various key states, microstates, triggering methods, and detailed transition pathways were studied in detail (Table S2) and mapped onto a circuit-like diagram (Fig. 2). Based on the crystallinity (*χ*_*c*_) and Hermans’ orientation factor (*f*) of A- and B-block domains, eight key states were identified, including three hydrogel states (NC’Am, NCAm, and MCAm) and five dry states (NCNC, NCHC, MCMC, MCHC, and HCHC). In the hydrogel states, the B-block matrix was amorphous and swollen with water. Wide-angle X-ray scattering (WAXS) revealed scattering peaks from the A-block domains, corresponding to the 110 planes of the orthorhombic PCL crystal structure (Figs. S7 and S8, Table S3). Soft and elastic mechanical properties were thus expected, with Young’s modulus ranging from 0.1 to 1.8 MPa. In comparison, dry states displayed extra scattering peaks of 120 and 032 planes of the monoclinic PEO crystal structure (Figs. S7 and S8, Table S3). Mechanical performance was significantly enhanced compared to hydrogel states, with modulus ranging from 0.13 to 0.88 GPa and engineering strength from 42 to 388 MPa.

Using eight mild stimuli as triggers, eight key states can switch between each other through a network of 31 transition pathways (Fig. 2 and Table S2). As detailed below, the regulation of the four system parameters (*χ*_c,A_, *χ*_c,B_, *f*_A_, and *f*_B_) is not fully independent during state transitions—depending on the trigger, they may be coupled or changed separately. Having the lowest modulus, NC’Am functions as a transition hub connecting to six other key states through 13 pathways. Through freezing to RT under water (Trigger 1) of NC’Am, *χ*_*c*,A_ increases more than twofold from 7.9 to 22.2%, leading to NCAm with much higher strength and modulus. Cold strain under water (Trigger 3) converts NC’Am into MCAm through strain-induced crystallization. This is a continuous transition (Table S2 and Fig. S2) in which *χ*_*c*,A_ and *f*_A_ develop jointly and reach 16.6% and −0.35, respectively, indicating that chain orientation and crystallization proceed as a coupled process. This *f* value, which defines the MC state in our system, is the upper bound of PCL alignment attainable in the hydrogel state, set by the constrained mobility of PCL crystallites within the swollen PEO matrix. Both NCAm and MCAm can switch back to NC’Am through 60 °C to RT under water (Trigger 2), which induces melt recrystallization of the A-block domains.

Hydrogel states are transformed into dry states with minimal water content (Fig. S18) by dehydration (Trigger 5) or dehydration under strain (Trigger 6), where crystallization of the B-block matrix leads to a significant increase in modulus and strength. With dehydration (Trigger 5), both NCAm and NC’Am transform into NCNC with isotropic A- and B-block domains (*f*_A_ = *f*_B_ = 0) via quiescent crystallization. In comparison, MCAm is converted to MCMC with both domains aligned (*f*_A_ = −0.32, *f*_B_ = −0.28) via Trigger 5, highlighting the template effect of A-block during B-block crystallization^42,43^. Dehydration under strain (Trigger 6) enabled continuous transition of NC’Am, NCAm, and MCAm into various dry states ranging from NCHC to HCHC, depending on the applied strain (Table S2 and Figs. S3–S6). NCAm transforms into NCHC with a highly-aligned B-block matrix (*f*_B_ = −0.42) even with little applied strain, indicating that the B-block matrix is easily orientated during crystallization.

Hydration (Trigger 4) converts dry states into hydrogel states by dissolving crystallites in the B-block but not the A-block, accompanied by volumetric swelling (Fig. S17). As expected, NCNC/NCHC transforms into NCAm, and MCMC/MCHC into MCAm, with A-block alignment conserved. Notably, hydration of HCHC leads to MCAm rather than HCAm, indicating that amorphous and isotropic B-block matrix reduces the alignment of A-block domains when *f*_A_ is too high (*f*_A_ changed from −0.45 to −0.35). Among the dry states, eight different transition pathways exist based on Trigger 7 (60 °C to RT) and 8 (cold strain). Trigger 7 leads to the removal of alignment in both A- and B- blocks through melt recrystallization, switching other dry states into NCNC. Trigger 8 (cold strain) of NCNC, NCHC, MCMC, and MCHC leads to HCHC, where high crystallinity (*χ*_*c*,A_ = 35.0%, *χ*_*c*,B_ = 89.8%) and orientation factors (*f*_A_ = −0.45, *f*_B_ = −0.47) result in a combination of extremely high strength and toughness.

### Mechanical versatility of MMC by phase control of physically crosslinked microdomains

**Fig. 3:**
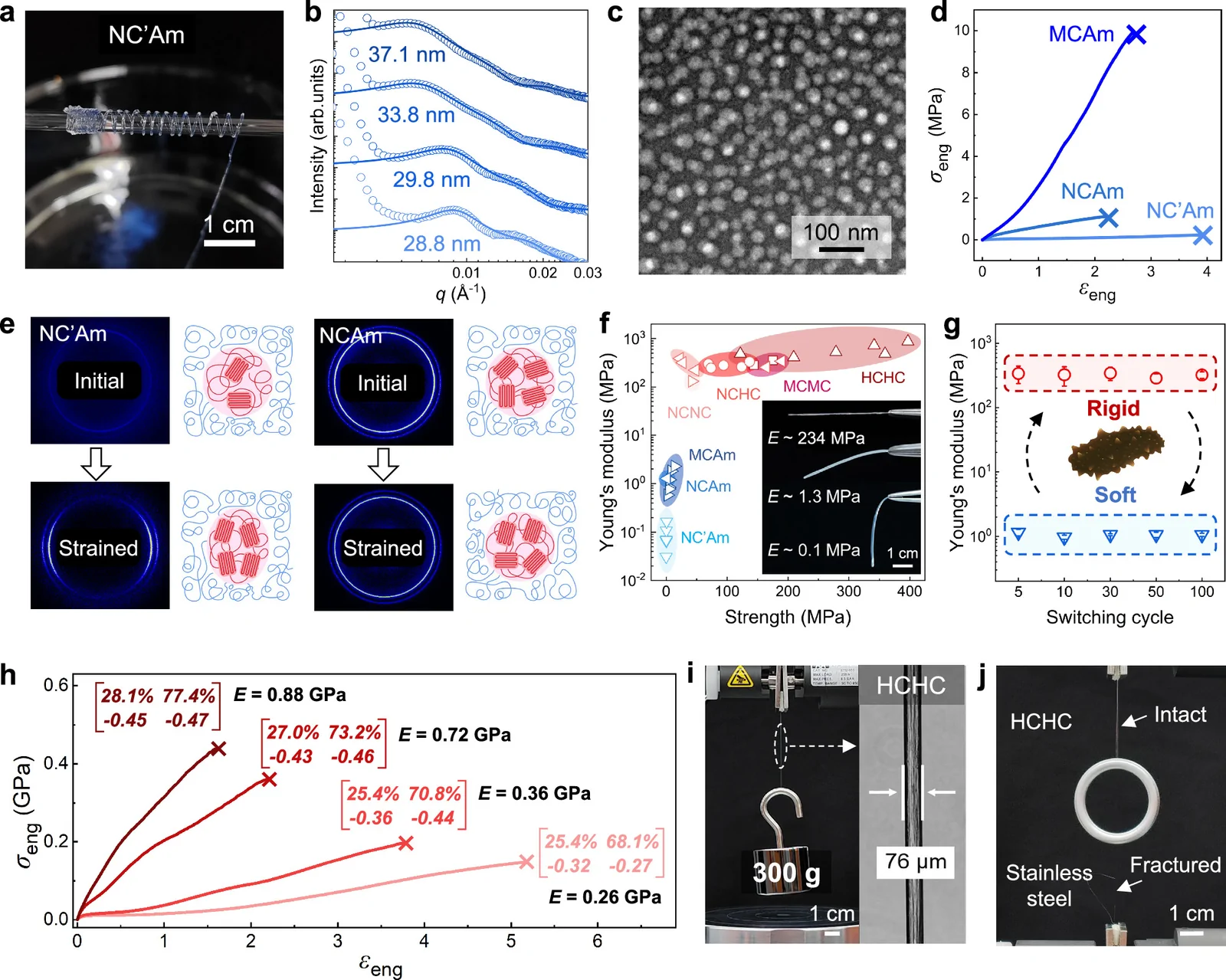
The MMC system displays a wide range of mechanical properties. **a** NC’Am in a fiber form. **b** Experimental and modeled SAXS data of NC’Am with different microdomain sizes from C_400_O_1818_C_400_ of different concentrations (5 wt%, 10 wt%, 15 wt%, and 17 wt%). **c** TEM image of the physically crosslinked disordered spherical microdomains. Similar results were obtained in three independent experiments. **d** Tensile stress–strain curves demonstrate an increasing trend of mechanical strength in the order of NC’Am, NCAm, and MCAm. **e** After strain, A-block domains become aligned with higher crystallinity in NC’Am but not in NCAm. **f** MMC covers modulus across several orders of magnitude. **g** The system can switch between the low-modulus (NCAm) and high-modulus (NCNC) states more than 100 cycles without performance decay. Circles and triangles show mean values, and error bars represent the standard deviation (s.d.) (*n* = 3 independent measurements). **h** Regulation of mechanical properties by fine-tuning microstates between MCMC and HCHC. **i** HCHC can hold a million times its own weight (0.3 mg fiber holds a weight of 300 g). **j** HCHC fractured stainless steel in a direct tensile test against each other. HCHC and stainless-steel fiber were both 10 cm in length and 0.3 mg in weight.

Rapid injection of triblock copolymer COC in water leads to nonsolvent-induced microphase separation and formation of NC’Am (Fig. 3a). Using small-angle X-ray scattering (SAXS) and negatively stained transmission electron microscopy (TEM), disordered spherical micelles from block copolymer self-assembly were observed (Figs. 3b, c and S9–S11, Table S4). In the TEM images, the light-colored spherical domains correspond to the unstained PCL micelle cores, while the hydrophilic PEO matrix appears dark due to negative staining with uranyl EM stain. The SAXS data were modeled with a spherical form factor and a Percus–Yevick structure factor^48,49^, with radius and center-to-center distance of the micelles increasing with the molecular weight of the hydrophobic block and concentration of precursor polymer solution (Figs. 3b and S9–S11, Table S4). Importantly, for the model system used for the rest of the study (C_400_O_1818_C_400_ at 17 wt%), the microdomain size remains essentially constant across the hydrogel and dry states (Fig. S11b).

Due to isotropic alignment, low crystallinity of A-block domains and a high degree of water swelling in B-block matrix (Fig. S13), NC’Am is soft and stretchable (Figs. 3d and S12, S14, Tables S5, S6), and demonstrates high transformability with direct access to six other key material states. In comparison, NCAm is less deformable due to higher crystallinity (*χ*_*c*,A_ = 22.2%) and rigidity of A-block domains, where trigger 3 leads to a significant increase of *χ*_*c*,A_ and *f*_A_ in NC’Am but not NCAm (Fig. 3e). NCAm shows an increase in strength and modulus but a decrease in stretchability compared to NC’Am (Figs. 3d and S12, Table S5). By further increasing alignment (*f*_A_ = −0.35), MCAm exhibits the highest modulus (1.8 MPa) and engineering strength (8.8 MPa) among hydrogel states (Figs. 3d and S12, Table S5).

Hydrogel and dry states switch between each other through hydration and dehydration of the B-block matrix. In combination, the material system covers a wide range of strength and modulus (Fig. 3f), fulfilling the needs of diverse material designs. The modulus of the system can be varied rapidly and reversibly, reminiscent of the response of sea cucumber dermis^15,16^. Sea cucumbers change the modulus of their nanostructured connective tissue by regulating aggregation states of collagen fibrils to enhance survival, inspiring the development of synthetic mimics for emerging applications in smart fabrics, medical implants, and soft robotics^16,29,50,51^. In our system, switching from HCHC to NC’Am via trigger 2 leads to an 8800-fold decrease in Young’s modulus (from 0.88 GPa to 0.1 MPa) within seconds. To illustrate the reversibility of modulus changes, the system was switched between NCAm and NCNC over 100 cycles without performance decay (Figs. 3g and S15 Tables S7, S8). The system exhibited the same self-assembled nanostructure when reset to NC’Am, attributed to physical crosslinks formed by phase-separated ABA triblock copolymers (Fig. S11b). Notably, MMC offers a large modulus switching range and significantly improved mechanical properties like toughness and strength (Fig. S16) compared to previously reported work (Table S9).

By controlling the strain value of Trigger 6 (dehydration under strain), MCAm undergoes continuous transitions into a series of fine-tuned microstates between MCMC and HCHC (Figs. 3h and S19, S20). Simultaneous increases of *χ*_*c*_ and *f* in A and B blocks lead to enhanced mechanical properties, which peak at HCHC with engineering strength of 388 MPa and toughness of 440 MJ/m³ (Figs. 3h and S20). This unusually high combination of strength and toughness was further demonstrated by an HCHC fiber of 0.3 mg in mass holding a weight of 300 g, a million times its own weight (Figs. 3i and S21, Supplementary Movie S1). In a direct comparative tensile test, HCHC fractured stainless steel of equal length and mass, demonstrating superior mechanical properties (Figs. 3j and S21, S22, Supplementary Movie S2).

### Fine-tuning deformation behaviors of necking for damping applications

**Fig. 4:**
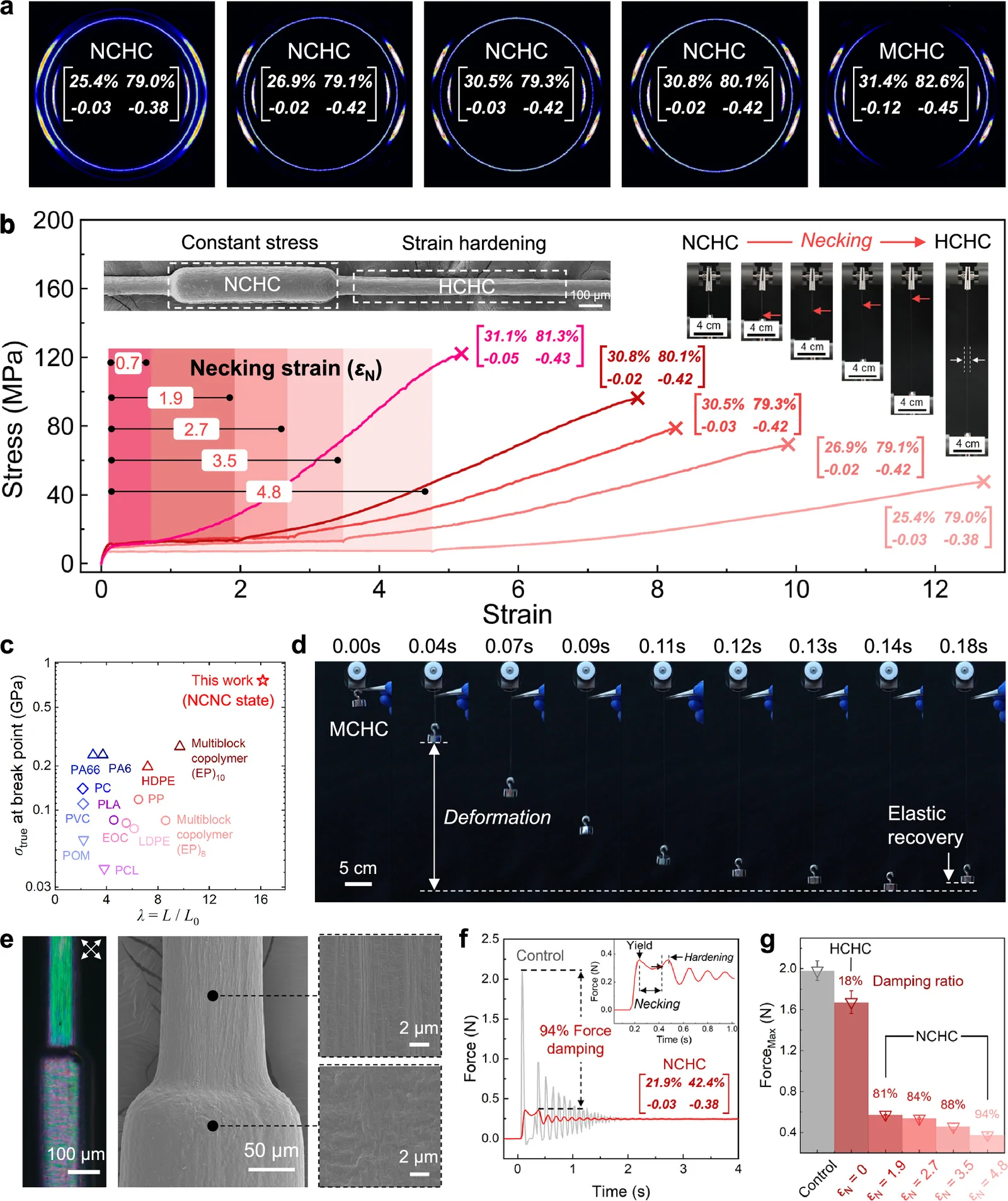
Tunable necking and strain-hardening behaviors for high-efficiency impact damping. **a** Fine-tuned state between NCHC and MCHC. **b** Stress-strain curves of fine-tuned NCHC states exhibit tunable necking and strain-hardening behaviors. Strain rate was 1 min^−1^. Left inset: a single fiber containing both NCNC and HCHC regions, prepared by applying trigger 8 (cold strain) to a portion of an NCNC sample; the strained region converts to HCHC, while the remainder stays as NCNC. Right inset: necking propagation of NCHC. **c** Comparison of this work with other necking and strain-hardening polymers. **d** Impact force damping of MCHC with a dropping weight of 25 g. **e** POM and SEM images of a partially necked sample of NCNC. The necked part displayed highly aligned structures due to fibrillation (Figs. S28 and S29). Similar results were obtained in three independent experiments. **f** NCHC displayed 94% reduction of impact force compared to HDPE. **g** Impact force damping efficiency of HCHC and NCHC with different necking strains. Triangles show mean values, and error bars represent the standard deviation (s.d.) (*n* = 3 independent measurements).

Necking develops as a localized deformation process during tensile loading of semi‑crystalline polymers, where fibrillation and strain-hardening prevent sudden failure by redistributing stress. In our system, a stress-constant and tunable plateau of necking followed by intense strain-hardening was observed during the tensile test of NCNC, NCHC, and MCHC (Figs. 4a, b and S23–26). By applying trigger 6 (dehydration under strain) to NC’Am at varying strain levels, a series of stable and continuous microstates can be achieved (e.g., NCHC and MCHC), leading to a tunable strain range of necking window based on the chain orientation across crystalline and amorphous regions (Fig. S25), allowing an extremely large necking strain^52,53^ (*ε*_N_ = 5.5) to be achieved (Figs. S23 and S26). Notably, the four-parameter matrix (*χ*_c,A_, *χ*_c,B_, *f*_A_, and *f*_B_) describes the crystalline component of the network and does not fully capture chain orientation in the amorphous regions. When samples share comparable values across these four parameters, differences in amorphous chain orientation can still produce substantially different stress–strain responses, as seen across the curves in Fig. 4b.

Considering the highly non-uniform strain distribution induced by necking, we further characterized the true stress-strain curve of NCNC to better reflect the load-bearing and strain-hardening properties (Fig. S27). Upon stretching, physical crosslinks and microphase separation enabled the PCL and PEO domains to develop high values of *χ*_*c*_ and *f*. This enhanced structure yielded an exceptionally high true strength (*σ*_true_ = 738 MPa) and elongation-at-break value (*λ* = 16). These mechanical properties not only surpassed those of PCL homopolymer significantly, but also outperformed other strain-hardening and necking polymers previously reported (Fig. 4c).

As indicated by polarized optical microscope (POM) and scanning electron microscopy (SEM), necking leads to highly aligned fibril-like structures due to strain-induced melt recrystallization, which provide high strength and toughness supporting further strain-hardening (Figs. 4e and S28, S29). Using cold strain (Trigger 8) as a convenient processing method, it is also possible to integrate states with and without necking behavior into the same specimen (Fig. 4b, inserted). NCNC, NCHC and MCHC with large-strained necking and intensive strain-hardening provide great opportunities for impact damping applications (Figs. 4d and S30, Supplementary Movie S3). Owing to its remarkable toughness and strength, HCHC demonstrated great impact resistance (Figs. S31 and S32, Supplementary Movie S4), surpassing high-performance impact-resistant materials like UHMWPE and Kevlar (Figs. S33 and S34). Attributed to the wide and stress-constant necking plateau, NCHC can reduce impact force up to 94% (Figs. 4f and S35), and is capable of absorbing three times more impact energy compared to HCHC. Impact force damping ratio of different material states scales with the *ε*_N_ value (Fig. 4g), which allows designing tunable cushion materials that dissipate energy across a broad intensity spectrum.

### Rapid transitions for actuation applications

**Fig. 5:**
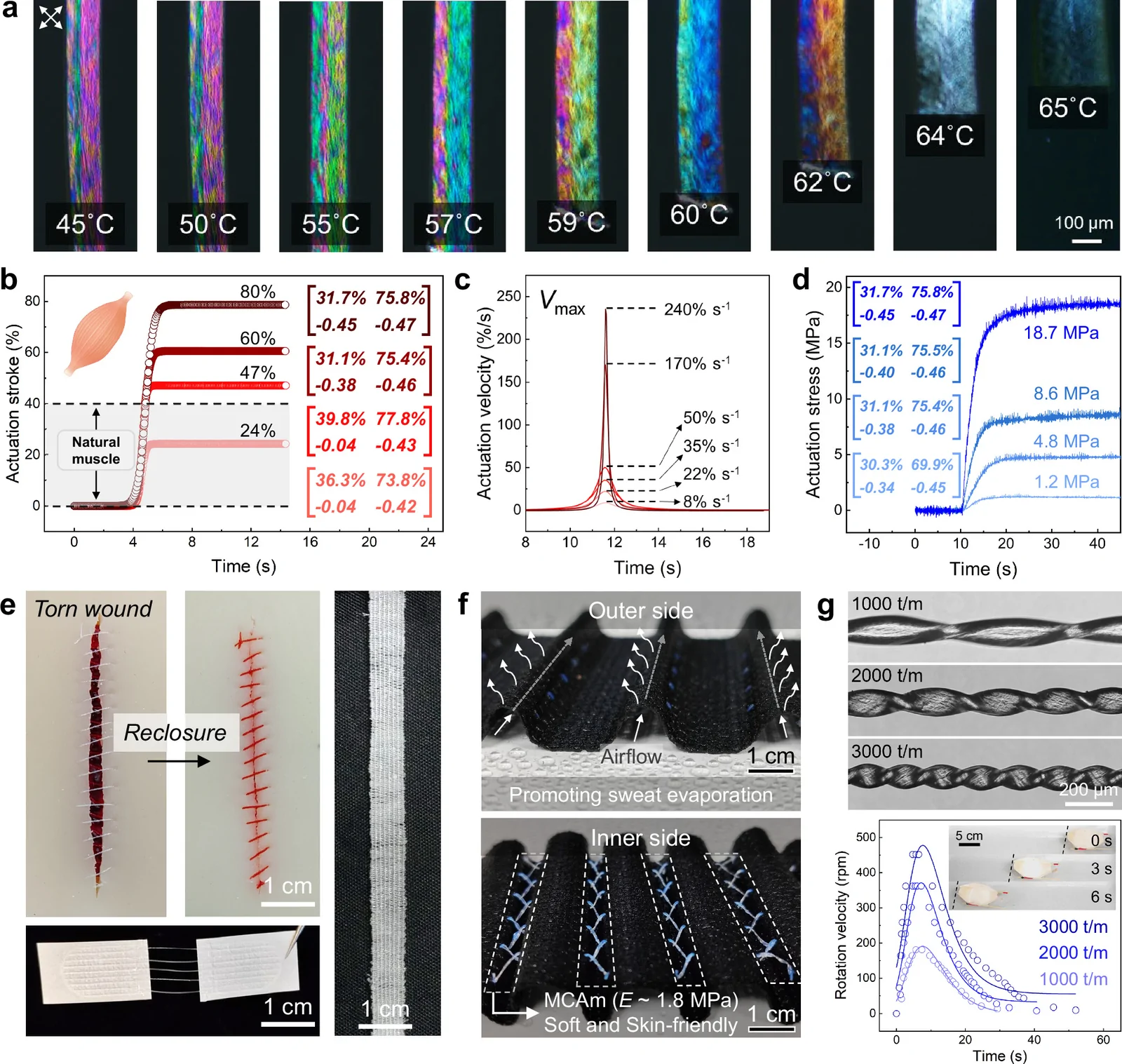
Actuation applied for self-closure sutures, smart fabrics, and rotational motors. **a** POM images of HCHC at different temperatures indicate actuation starts around 60 °C. **b–d** Tunable actuation stroke, velocity and stress through parameter control of material states. Heat was applied by vertically suspending the fiber between two hot plates. **e** Self-closure sutures, tension-reducing tapes, and biomedical fabrics can automatically close wounds when contacting blood. **f** HCHC, as inlaid in a smart garment, was switched to MCAm when triggered by sweat, which accelerates airflow and heat dissipation. **g** Top: Optical microscope images of rotational motors programmed at 1000, 2000 and 3000 turns/meter. Similar results were obtained in three independent experiments. Bottom: The motor demonstrates fast and tunable rotational speed and can be used to power model boats.

Through mild stimuli like hydration and gentle heating (60 °C), aligned states like HCHC, MCHC, and NCHC go through rapid transformation into more isotropic states like NCNC, NCAm and MCAm in seconds. Combined with great mechanical properties, the system enables programmable high-performance actuation with key parameters like stroke, velocity, stress, and energy density surpassing natural skeletal muscles (Figs. 5b–d and S38, S40–S42).

Through heating, material states before and after transition both display high modulus, which prevents deformation and provides a facile on-off switch of actuation (Supplementary Movie S5). Aligned dry states melt around 60 °C to generate actuation and then recrystallize isotropically to form NCNC at room temperature (Figs. 5a and S36, S37). In comparison, actuation via hydration will selectively swell the B-block matrix in water and lead to a soft hydrogel state MCAm, which permits continuous actuation until total release of the stored energy (Figs. S39–S41 and Supplementary Movie S6). A lower PCL block ratio (C_215_O_1818_C_215_) was found to enhance actuation performance, yielding increased energy density and actuation velocity (Fig. S42). Through fine-tuning of the material states, controllable actuation performance was achieved with a stroke of up to 80%, stress up to 18.7 MPa, and energy density up to 300 J/kg (Figs. 5b–d and S38, S42), all exceeding natural skeletal muscle (stroke of 40%, stress of 0.35 MPa, and energy density of 39 J/kg). Across the parameter matrix, actuation is governed primarily by block-B orientation under hydration and by both blocks under heating. Under hydration, the driving force is the loss of PEO orientation. Although *f*_B_ is nearly saturated, chain orientation in the amorphous PEO regions continues to develop with strain and contributes to the actuation response.

The MMC platform supports applications―biomedical devices, smart fabrics, and microengines―that conventional stimuli-responsive materials typically handle with separate systems (Figs. S43–S48). Aligned fibers were twisted into two- or four-strand yarns for developing smart biomedical devices and fabrics (Figs. 5e and S43). Sutures and tension-reducing tapes automatically tighten with programmable stroke and stress, and reclose torn wounds in situ upon contact with blood (Figs. 5e and S44, S45, Supplementary Movie S7). The yarn modulus is tunable across the soft-tissue range, so contraction transfers less strain to the wound margin (Figs. S44 and S45). The fibers display good biocompatibility with low hemolytic activity and cytotoxicity (Figs. S50–S52).

Co-woven with commodity textile, HCHC yarns act as sweat-triggered thermal-management elements, opening ventilation channels to increase local airflow and heat dissipation (Figs. 5f and S46, Supplementary Movie S8). Actuation is driven directly by the perspiration that signals the need for cooling, providing on-demand ventilation without external power. Twisted MMC yarns actuated by dehydration under strain function as rotational engines (Figs. 5g and S47), which power a model boat with fast and tunable rotational speed (up to 500 rpm) and high recovery rate (*R*_r_) of 90% (Fig. S48)^17,47,54^. Across these demonstrations, the actuating element is a single, unfilled material without chemical crosslinking, simplifying fabrication and end-of-life processing (Fig. S49) relative to composite or crosslinked materials.

## Discussion

In this work, we present a multistate material circuit framework that enables on-demand control and reversible switching among distinct material properties. Integrating physical crosslinks with crystallinity and orientation regulation of self-assembled microdomains, the system exhibits a broad property range, spanning from soft and elastic to strong and tough. Continuous transitions with quantitatively defined microstates enabled fine-tuning of deformation behaviors and provided superior impact damping. Discrete transitions allowed sea-cucumber-like rapid modulus switching, coupled with excellent mechanical performance and biocompatibility, which is ideal for designing adaptive fabrics and biomedical devices. Programmable and versatile actuators were also realized with stroke, stress, and energy density surpassing those of natural skeletal muscles. Composed of a single composition without covalent crosslinking, the system is readily recyclable. This approach, combining functional versatility with material economy, demonstrates great potential for developing an efficient and sustainable material production and usage cycle with diverse applications.

## Methods

### Materials

HO-PEO-OH (*M*_n_ = 80 kg/mol) was purchased from JenKem Technology Liaoning Ltd., and dried by azeotropic distillation with anhydrous toluene. Stannous isooctanoate (Sn(Oct)_2_) and ε-caprolactone (ε-CL) were obtained from Shanghai Aladdin Biochemical Technology Co., Ltd. Sn (Oct)_2_ was dried by azeotropic distillation with anhydrous toluene, ε-CL was purified with CaH_2_ for 48 h, vacuum distilled, and protected with argon until use.

### Synthesis of PCL-PEO-PCL triblock copolymer

Triblock copolymer poly(ε-caprolactone)-b-poly(ethylene oxide)-b-poly(ε-caprolactone), COC, was synthesized via ring-opening polymerization (ROP). In a typical synthesis, 2 g HO-PEO-OH (80 kg mol^−1^, 0.025 mmol), 2.4 mL ε-CL (2.5 g, 21.9 mmol) and 32.4 μL Sn(Oct)_2_ (0.1 mmol, 40.5 mg) were added into a dried reaction flask, followed by the addition of 20.8 mL anhydrous toluene. The reaction flask was evacuated, sealed, and then placed in 130 °C oil bath for polymerization of 36 h. The obtained reaction mixture was dissolved in 20 mL of dichloromethane (DCM) and precipitated in the mixture of 450 mL petroleum ether (PE) and 50 mL ethanol (EtOH) three times. The obtained precipitation was vacuum dried at room temperature for 48 h to obtain COC, which was characterized by ^1^H NMR (AVANCE NEO 400, CDCl_3_) and size-exclusion chromatography (EcoSEC HLC-8320GPC, Tosoh Bioscience) with THF as the mobile phase (Data collection software: EcoSEC Elite-WS).

### Preparation of COC at NC’Am state

A predetermined amount of COC polymer was dissolved in THF to prepare a 17 wt% precursor solution. The solution was transferred into a 5 mL syringe and allowed to stand to remove trapped air. Using an automated syringe pump, the precursor solution was injected at a rate of 0.5 mL min^−^^1^ through needles with inner diameters of 1 mm or 0.3 mm into an excess of deionised water. The rapid solvent exchange between THF and water induced microphase separation, generating micellar physical crosslinking points and yielding hydrogel fibers in the NC’Am state.

### Transmission Electron Microscopy (TEM) characterization

The micelles and microgel formed by self-assembly of COC were characterized by Transmission Electron Microscopy (TEM), JEM 1400 Plus (JEOL), with an accelerating voltage is 120 kV. Firstly, injecting 100 μL THF solution of COC (0.5 wt% ~2 wt%) into 2 mL deionized water to form a suspension of micelle or microgel, then transferring 5 μL suspension onto a thin pure carbon support film pretreated with an ion sputtering coater, SBC-12 (Beijing Zhongjingkeyi Technology Co., Ltd.). After staining with the Uranyl EM stain, the prepared sample was observed in TEM.

### Scanning Electron Microscope (SEM) characterization

The surface morphology of the fabricated fibers was characterized using field emission scanning electron microscopy (JEOL JSM-7900F). For hydrogel samples (NC’Am, NCAm, and MCAm), cryogenic lyophilization was first performed to preserve their intrinsic microstructures. The freeze-dried samples were then mounted on aluminum stubs using conductive carbon tape and sputter-coated with a gold layer to enhance surface conductivity. Finally, the samples were imaged under high vacuum conditions at an accelerating voltage of 5 kV. Data collection software: JEOL PC-SEM.

### Tensile test

Uniaxial tensile tests were conducted on a Universal Testing Machine (INSTRON, 68TM-30) with a 100 N load cell. The strain rate is 100% min^−1^. The size of fibers was measured using a vernier scale and an upright-inverted integrated motorized fluorescence microscope (Revolve Generation 2). All experiments were repeated independently *n* = 3 times with similar results. Data collection software: Bluehill Universal.

Engineering stress and strain were used throughout the paper and SI unless otherwise stated. The engineering strain *ε*_eng_ was calculated by the following equation:

$${\varepsilon }_{{{{\rm{eng}}}}}=\frac{L-{L}_{0}}{{L}_{0}}=\frac{\varDelta L}{{L}_{0}}$$

Where *L*_0_ is the initial length of testing sample, *L* is the current length of sample. The engineering stress *σ*_eng_ was calculated by the following equation:

$${\sigma }_{{{{\rm{eng}}}}}=\frac{F}{{A}_{0}}$$

Where *F* is the current load, *A*_0_ is the original specimen cross-sectional area.

The Young’s modulus of the fiber samples can be evaluated by linear fitting the initial part of the stress-strain curves using the following equation:

$${\sigma }_{eng}=E{\varepsilon }_{eng}$$

Where *σ*_*eng*_ is the engineering stress, *ε*_*eng*_ is the engineering strain, and *E* is Young’s modulus. The engineering stress at the break point was taken as the strength, and the toughness was calculated by integrating the stress-strain curves.

The true strain *ε*_true_ (Hencky strain) is calculated by the following equation:

$${\varepsilon }_{{{{\rm{true}}}}}=\,{{\mathrm{ln}}}\,\lambda=\,{{\mathrm{ln}}}\,\frac{L}{{L}_{0}}$$

Where *λ* is the elongation ratio. The true stress *σ*_true_ was calculated by the following equation:

$${\sigma }_{{{{\rm{true}}}}}=\frac{F}{A}$$

The current specimen cross-sectional area *A* was recorded using a CCD camera. All mechanical tests were conducted at room temperature (25 ± 1 °C) and ambient relative humidity (65 ± 10% RH).

### Small-angle X-ray scattering (SAXS) characterization

The SAXS tests were performed on Xenocs Xeuss 3.0 HR Small-Angle X-ray Scattering (Liquid Ga-In alloy target, *λ* = 1.34 Å (Ga Kα), focal spot size ≤ 20 μm), or at the BL10U1, BL19U2, and BL16B1 beamline of Shanghai Synchrotron Radiation Facility (SSRF). The wavelength of X-ray is 1.03 Å, the exposure time for each frame was controlled at 10 s. The sample-to-detector distances were 2030 mm. The samples were encapsulated between two polyimide (Kapton) films to prevent dehydration during measurements. Data collection software: XSACT Pro (Xenocs).

The scattering vector *q*, which is defined as:

$$q=\frac{4\pi \,\sin (\theta )}{\lambda }$$

Where *θ* is the scattering angle calibrated by the diffraction pattern of silicon (Si), and *λ* is the X-ray wavelength.

The SAXS plot was fitted using the Percus-Yevick (PY) model to give the architectural configuration of the fiber materials^55^.

### Wide-angle X-ray scattering (WAXS) characterization

#### Instrumentation

The experiments were performed on a two-dimensional wide-angle X-ray scattering system (Rigaku Corporation, HomeLab) (Cu Kα radiation, *λ* = 1.5405 Å) equipped with a HyPix-6000 photon-counting detector (Pixel size: 100 × 100 μm). Beam conditions: 45 kV, 60 mA, collimated to 0.07 mm diameter. The sample-to-detector distance was calibrated using a silicon standard. Data collection software: NANOPIX Guidance.

#### Sample preparation

Dry fiber samples were mounted directly onto the sample holder using high-vacuum-compatible adhesive tabs, with ambient air scattering serving as the background reference for data processing. For hydrated hydrogel fibers, specimens were first encapsulated between two polyimide (Kapton) films to prevent dehydration during measurements, then secured on the holder. Ultrapure water was measured under identical conditions to provide the background subtraction profile for hydrogel samples.

#### Data processing

Raw 2D images were integrated into 1D intensity profiles (*I* ~ *q*) using the Fit2D software (Version 18, ESRF). Background subtraction was performed using an air (dry samples) or water (hydrogels) scan. The crystallinity index was calculated via peak deconvolution (Gaussian fitting) of amorphous and crystalline scattering contributions^47^. For example, the crystallinity of PEO was calculated by the following equation:

$${X}_{{{{\rm{c}}}},{{{\rm{PEO}}}}}=\frac{{A}_{PEO,crystalline}}{{A}_{PEO,crystalline}+{A}_{{{{\rm{PCL}}}},{{{\rm{crystalline}}}}}+{A}_{amorphous}}\frac{1}{{\omega }_{{{{\rm{PEO}}}}}}$$

*ω*_PEO_ indicates the mass fraction of PEO block, for C_400_O_1818_C_400_, *ω*_PEO_ is 46.5 wt%. *A*_PEO_, _crystalline_ and *A*_PCL, crystalline_ represent the integrated area of the PEO and PCL crystalline diffraction peak. *A*_amorphous_ represents the integrated area of the amorphous haloes.

The orientation degree of the PEO and PCL crystal in different fiber samples were quantified by the orientation factor <cos^2^*φ*> and Hermans’ orientation factor *f* using the following equations:

$$\langle {\cos }^{2}\varphi \rangle=\frac{{\int }_{0}^{\pi }I(\varphi ){\cos }^{2}\varphi \,\sin \varphi {{{\rm{d}}}}\varphi }{{\int }_{0}^{\pi }I(\varphi )\sin \varphi {{{\rm{d}}}}\varphi }$$

$$f=\frac{3({\cos }^{2}\varphi )-1}{2}$$

Where *I(φ)* is the scattering intensity by the (hkl) plane as a function of the azimuthal angle *φ* (relative to the reference direction).

The crystalline orientation was quantitatively analyzed through a two-step approach: Firstly, the azimuthal intensity profiles were initially fitted with Lorentz functions, and then the fitted curves were incorporated into Hermans’ orientation factor equation to obtain the *f*-factor, which provides a quantitative measure of the orientation degree of the crystal. Here, −0.1 <*f* < 0, −0.35 <*f* < −0.1 and −0.5 <*f* < −0.35 are defined as non-aligned semi-crystalline, medium-aligned semi-crystalline and highly-aligned semi-crystalline, respectively.

### Reporting summary

Further information on research design is available in the Nature Portfolio Reporting Summary linked to this article.

## Supplementary information

Supplementary Information

Description of Additional Supplementary Files

Supplementary Movie 1

Supplementary Movie 2

Supplementary Movie 3

Supplementary Movie 4

Supplementary Movie 5

Supplementary Movie 6

Supplementary Movie 7

Supplementary Movie 8

Reporting Summary

Transparent Peer Review file

## Source data

Source Data Files

## Data Availability

The source data underlying all figures are provided with this paper as a Source Data file. All data are available from the corresponding author upon request. Source data are provided with this paper.
