# Supplementary material for: Block copolymer multistate material circuit
Source: Nat Commun. 2026 Oct 6;17:11393. doi: 10.1038/s41467-026-78278-2 (PMC13736946; doi:10.1038/s41467-026-78278-2)
Supplement: Supplementary file 2 [file 41467_2026_78278_MOESM12_ESM.pdf]

nature portfolio

Peer Review File

Block copolymer multistate material circuit

Corresponding Author: Professor Chao Lang

This file contains all reviewer reports in order by version, followed by all author rebuttals in order by version.

Version 0:

Reviewer comments:

Reviewer #1

(Remarks to the Author)
The authors proposed the multistate material circuit based on crystalline PCL-PEO-PCL triblock copolymers. Two block domains with distinct crystallization abilities were controlled separately to tune the mechanical properties. The designed mechanical response is able to cover the high toughness and excellent impact resistance. For such complex double-crystalline polymers, an innovative idea is to propose the crystallinity-orientation parameter matrix to detailedly describe their various states. This approach will be useful in development of efficient and sustainable materials. This manuscript is well organized. However, there are some concerns that should be addressed

-It is new to propose 2x2 parameter matrix to quantify the various states of crystalline block copolymers from the structural point of review. What is more important is that various states were correlated with the corresponding mechanical properties covering high toughness/strength and excellent impact resistance. For PCL, the crystallinity was increased by the progressive strain-induced crystallization, as explained in Figure S7 caption. Then, it is important to clearly explain whether these four parameters can be regulated freely or are always coupled with each other. It would be helpful to more clearly explain the specific role of each parameter in the properties explored.

-Fig. 3b clearly shows that microdomain size largely changed from 28.8 nm to 37.1 nm. Firstly, sample names and preparation methods were missing. Secondly, such large change suggests that microdomain size probably also plays an unavoidable role in affecting mechanical response and should be considered as a parameter as well. However, microdomain size was not included in parameter matrix. Thirdly, TEM image show the formed spherical microdomains. It remains unclear which polymer block, PCL or PEO, to form these spherical microdomains. It is wise to briefly explain how to verify the component of dispersed microdomains.

-Fig. 4b presents the stress-strain curves of fine-tuned NCHC states with various necking and strain-hardening behaviors. Notably, the first three samples have similar parameters but quite different stress-strain curves. Does this mean that the mechanical response is very sensitive to a slight difference in A-block crystallinity or orientation? If so, the precise degree of crystallinity should be very important. It was only roughly introduced that crystallinity was analyzed from 1D WAXS plot. In addition, for all samples of Fig. 4b, the sum of PCL and PEO crystallinities is larger than 100%. It seems that the diffraction halo was determined for PCL and PEO separately to calculate individual crystallinity. However, Figs. S7b and S9a show that the characteristic diffraction peaks are located within close scattering vector range. How to determine the PCL and PEO amorphous haloes from the overlapped diffraction signals? The detailed analysis method can be provided.

There are four parameters in the parameter matrix to distinguish different states. It will make more sense to explain the reported properties by these parameters. The authors are suggested to explain the specific role of each parameter in determining properties of interest.

-The axis of Fig. S24 should be elongation ratio, rather than elongation-at-break ratio.

-Fig. 4b inset shows the specimen that integrates states with and without necking. Normally, necking appears and then propagates in two directions within sample. As necking requires the same stress for various positions, propagation apparently shows a constant stress, leading to a constant stress plateau in engineering stress-strain curve. After this propagation of local necking, the necked sample can be further deformed and gives the strain-hardening response. However, it is hard to understand why the so-called constant stress part was observed in the middle of two necking regions, see Fig. 4b. Does this mean that more than one necking processes were trigged within the same sample?

On the other hand, the necking strain that largely increased from 0.7 to 2.7 was emphasized by the authors. What is the physical meaning and cause of various necking strains?

-Fig. 5b and 5d show tunable actuation stroke and stress through parameter control of material states, respectively. When carefully examining these parameters, crystallinity values are quite close, while orientation changes clearly. Is it possible that orientation factor is the crucial parameter to determine actuation behaviors? Based on the results of Fig. 5d, it seems that the actuation stress is determined by block-A orientation factor fA.

-In many mechanical curves (for example Fig. S20), a stress drop appeared at the end of stress plateau but a rapid stress increase appeared before strain hardening. What is this peculiar stress change?

Reviewer #2

(Remarks to the Author)
In this manuscript, the authors report a multistate material circuit (MMC) system based on PCL–PEO–PCL triblock copolymers. The triblock copolymers exhibit eight distinct states depending on preparation conditions such as water content, temperature, and mechanical stress (strain), which serve as controlling parameters, with each state showing different mechanical properties. In addition, switching between these states can be controlled by externally added stimuli, such as humidity, temperature, and stress. The authors conducted extensive experiments and carefully investigated the structural characteristics, mechanical properties, and switching behavior of the MMC system. Furthermore, a number of practical demonstrations that exploit the multimodal and switchable properties of the system are presented.
Considering these points, the manuscript is likely to be of interest to the broad readership of Nature Communications. However, some details of the experimental conditions are missing, which may hinder the reproducibility of results. Therefore, the following points should be addressed.

1) Since the mechanical properties of polymers were highly affected by the water content and temperature, the authors should provide the quantitative experimental conditions, including humidity and room temperature. In addition, it would be important to show the water content of polymers in each state and to clarify the dehydrated state, including the conditions for its preparation.
2) If the dry material transforms into a hydrogel, a significant volume change is expected. Could the authors comment on the extent of the volume change between the hydrated and dehydrated states?
3) It would be helpful for readers to understand the clarification of eight states, if the authors could emphasize the reason why they included the series of materials with medium-aligned crystalline (MCAm, MCMC, and MCHC; f = –0.1 to –0.35) into the circuit as representative key states, because they seemed to be intermediate states during the transitions of NC to HC states.
4) Under what conditions can the AmAm state be obtained?
5) In pages 14–15, numerous applications are demonstrated without sufficient explanation. Even in the Supplementary Material, some experiments are described only in the figure captions, and the experimental conditions are unclear. For example, it is difficult to understand how the “hydration” of the fibers shown in Figs. S36–S38 and S42–S43 was carried out. In addition, the purpose of Fig. S42 is not clear. Where are the experimental results corresponding to Fig. S42e?
6) The demonstration section should be reorganized so that readers can clearly understand the significance of each demonstration, as well as what novel properties of the MMC materials offer advantages over previously reported stimuli-responsive materials.
7) The authors should rearrange the Supplementary Figures in the order they appear in the manuscript.

Reviewer #3

(Remarks to the Author)
I co-reviewed this manuscript with one of the reviewers who provided the listed reports. This is part of the Nature Communications initiative to facilitate training in peer review and to provide appropriate recognition for Early Career Researchers who co-review manuscripts.

Reviewer #4

(Remarks to the Author)
The article by Wei et al. is a thorough analysis of the mechanical properties of a triblock copolymer under various states of hydration and crystallinity. The introduction and results need to do a better job comparing the current materials to the literature. Strain-induced crystallization is an old phenomenon, and the mechanical properties of a material are obviously dependent on its state of hydration. As currently written, it is not clear how these results are surprising or how the purported "multistate materials circuits" are unique relative to what is already known about block copolymer materials. The work is certainly comprehensive, thorough, and worthy of publishing somewhere.

Version 1:

Reviewer comments:

Reviewer #1

(Remarks to the Author)
[Note from the Editor: Reviewer #1 was asked to assess the response to reviewer #4.]

All of my previous concerns have been properly addressed and the manuscript has been revised. The current manuscript could be considered to be published in Nature Communications.

The comments from reviewer 4 primarily focus on the innovation of the article, especially the novelty of the "multistate materials circuits" with respect to the known strain-induced crystallization. In response to these comments, authors provided explanations and revised the manuscript. They pointed that the structural regulation with quantitative parameters, crystallinity and orientation parameters, can facilitate systematic property control. From the material perspective, they emphasized that the structure-performance relationship tapped the application potential of double-crystalline polymers, where modulus, strength, and toughness were tuned over several orders of magnitudes. In my opinion, the responses and revisions are technically reasonable.

Reviewer #2

(Remarks to the Author)
The authors have responded sincerely and thoroughly to the concerns raised by this reviewer.
They have substantially revised the manuscript and provided additional data and discussion to support their conclusions. Therefore, I recommend this manuscript for publication in Nature Communications.

Reviewer #3

(Remarks to the Author)
I co-reviewed this manuscript with one of the reviewers who provided the listed reports. This is part of the Nature Communications initiative to facilitate training in peer review and to provide appropriate recognition for Early Career Researchers who co-review manuscripts.

Open Access This Peer Review File is licensed under a Creative Commons Attribution 4.0 International License, which permits use, sharing, adaptation, distribution and reproduction in any medium or format, as long as you give appropriate credit to the original author(s) and the source, provide a link to the Creative Commons license, and indicate if changes were made.
In cases where reviewers are anonymous, credit should be given to 'Anonymous Referee' and the source.
The images or other third party material in this Peer Review File are included in the article’s Creative Commons license, unless indicated otherwise in a credit line to the material. If material is not included in the article’s Creative Commons license and your intended use is not permitted by statutory regulation or exceeds the permitted use, you will need to obtain permission directly from the copyright holder.
To view a copy of this license, visit https://creativecommons.org/licenses/by/4.0/

Response to Editors and Reviewers' Comments

Block Copolymer Multistate Material Circuit

We sincerely thank the reviewers for the valuable comments and suggestions. To address these comments, we have revised the manuscript and SI systematically. Below we provide a detailed point-by-point response: reviewer comments are shown in *italics*, followed by our responses. Major revisions in the manuscript are highlighted in yellow. Note on figure numbering in SI: As requested by Reviewer #2 (Comment 2-7), we have reordered figures in SI to follow their citation order in the main manuscript (see detailed changes in **Table R1** on **Page R22**). We also refer to SI figures in this response using the following form (original number → new number).

Reviewer #1

The authors proposed the multistate material circuit based on crystalline PCL-PEO-PCL triblock copolymers. Two block domains with distinct crystallization abilities were controlled separately to tune the mechanical properties. The designed mechanical response is able to cover the high toughness and excellent impact resistance. For such complex double-crystalline polymers, an innovative idea is to propose the crystallinity-orientation parameter matrix to detailed describe their various states. This approach will be useful in development of efficient and sustainable materials. This manuscript is well organized. However, there are some concerns that should be addressed.

Comment 1-1: *It is new to propose 2×2 parameter matrix to quantify the various states of crystalline block copolymers from the structural point of review. What is more important is that various states were correlated with the corresponding mechanical properties covering high toughness/strength and excellent impact resistance. For PCL, the crystallinity was increased by the progressive strain-induced crystallization, as explained in Figure S7 caption. Then, it is important to clearly explain whether these four parameters can be regulated freely or are always coupled with each other. It would be helpful to more clearly explain the specific role of each parameter in the properties explored.*

Response: Thank you for raising this important point. The four parameters ($\chi_{c,A}$, $\chi_{c,B}$, f_A, and f_B) are neither fully independent nor strictly coupled, and the relationship between them depends on which trigger is applied. We discuss this in three cases below.

Coupled regulation: Within a given block, crystallinity (χ_c) and orientation factor (f) tend to evolve together when the trigger involves mechanical strain. In trigger 3 (cold strain under water) and trigger 6 (dehydration under strain), increasing strain raises both χ_c and f of the strained block (Fig. S7-S11 → Fig. S2-S6). Coupling also arises between blocks: in trigger 5 (dehydration) from MCAm to MCMC, the pre-oriented A-block (f_A = -0.35) acts as a template during B-block crystallization, producing an oriented B-block (f_B = -0.28) while f_A is largely retained (-0.32). The clearest illustration of inter-block coupling is trigger 8 (cold strain), which drives four states of NCNC, MCMC, NCHC, and MCHC all toward HCHC, simultaneously orienting both blocks.

Selective regulation with minor secondary changes: Trigger 7 (60°C to RT) from NCHC to NCNC selectively relaxes f_B from -0.42 to 0 while f_A remains 0. $\chi_{c,A}$ and $\chi_{c,B}$ change by only a few percentage

points, which we attribute to adjustments in crystallite population during orientation relaxation rather than to direct coupling. f_B can thus be tuned largely on its own when small χ_c variations are acceptable.

Selective regulation. Triggers 1 and 2 between NCAm and NC'Am modify $\chi_{c,A}$ (22.2% and 7.9%) while f_A remains 0; $\chi_{c,B}$ and f_B are not defined here because the B-block is amorphous in both states. These two triggers therefore provide a clean decoupling of $\chi_{c,A}$ from chain orientation, demonstrating that crystallinity and orientation are not intrinsically locked in this regime.

To better clarify this, the following text has been incorporated into the manuscript on **Page 6**, **Line 26**:

"As detailed below, the regulation of the four system parameters ($\chi_{c,A}$, $\chi_{c,B}$, f_A, and f_B) is not fully independent during state transitions—depending on the trigger, they may be coupled or changed separately."

On **Page 7**, **Line 3**, we added

"This is a continuous transition (Table S2, Fig. S7 → Fig. S2) in which $\chi_{c,A}$ and f_A develop jointly and reach 16.6% and -0.35, respectively, indicating that chain orientation and crystallization proceed as a coupled process."

On **Page S7** in SI, we have also added a detailed discussion regarding "selectivity and coupling among the four parameters".

Regarding the specific role of each parameter in properties:

Regarding the specific role of each parameter, the states examined here illustrate three representative cases: hydrogel-state mechanics dominated by A-block parameters, dry-state mechanics involving all four, and actuation governed by chain orientation. Details are elaborated as follows,

(1) **$\chi_{c,A}$ and f_A determine hydrogel-state mechanical properties.** Because $\chi_{c,B}$ = 0 in hydrogel states, only the A-block parameters are active and determines the mechanical properties. Comparison among NC'Am, NCAm, and MCAm reveals different contributions of $\chi_{c,A}$ and f_A: From NC'Am to NCAm (f_A = 0, $\chi_{c,A}$ increasing from 7.9% to 22.2%), all three metrics rise markedly: E increases from 0.1 to 1.3 MPa (~13-fold), σ from 0.2 to 1.1 MPa (~5.5-fold), and U from 0.4 to 1.4 MJ m^{-3} (~3.5-fold), indicating that $\chi_{c,A}$ establishes the fundamental mechanical framework of the hydrogel, with the most pronounced relative impact on Young's modulus. At comparable $\chi_{c,A}$ (NCAm vs. MCAm), varying f_A (0 to -0.35) selectively amplifies strength and toughness, where σ rises from 1.1 to 8.8 MPa (~8-fold) and U from 1.4 to 9.2 MJ m^{-3} (~6.6-fold), while E increases only modestly from 1.3 to 1.8 MPa (~1.4-fold). We have added the highlighted text on **Page S16** of SI to clarify this point.

(2) $\chi_{c,A}$, $\chi_{c,B}$, f_A, f_B **jointly determines dry-state mechanical properties.** In the dry states examined here, the four parameters act in the same direction: higher crystallinity and orientation lead to enhanced mechanical performance (modulus, strength, and toughness). Within this overall trend, the parameters play complementary roles. Matrix crystallization ($\chi_{c,B}$) dominates the hydrogel → dry transition; together with water removal, the increase of $\chi_{c,B}$ from 0 to 68–90% facilitates the modulus jump from MPa to sub-GPa scale (e.g., NCAm → NCNC: 1.3 MPa → 0.19 GPa). f_A and f_B amplify performance via orientation-induced reinforcement. Comparing NCNC and NCHC at comparable $\chi_{c,B}$, the introduction of a negative f_B (-0.42) approximately doubles E (0.19 → 0.35 GPa) and raises U by ~50%; comparing MCHC and HCHC, a more negative f_A further roughly doubles both E and σ, yielding HCHC's 0.91 GPa modulus and 445 MJ m^{-3} toughness. Because the four parameters are often linked by coupled crystallization pathways, one-variable-at-a-time isolation is not always achievable within this system. A systematic decoupling study—via composition variation or orthogonal processing—is a natural direction for future work. We have added the highlighted text on **Page S24** of SI.

(3) f_A **and** f_B **together determine actuation behavior**, with the trigger selecting which is released. Hydration releases the B-block orientation. Heating to 60°C melts both crystalline domains, simultaneously releasing f_A and f_B. See more mechanistic details in our response to Comment 1-7.

Comment 1-2: *Fig. 3b clearly shows that microdomain size largely changed from 28.8 nm to 37.1 nm. Firstly, sample names and preparation methods were missing. Secondly, such large change suggests that microdomain size probably also plays an unavoidable role in affecting mechanical response and should be considered as a parameter as well. However, microdomain size was not included in parameter matrix. Thirdly, TEM image shown the formed spherical microdomains. It remains unclear which polymer block, PCL or PEO, to form these spherical microdomains.*

Response: Thank you for your careful observation. We address each point below:

(1) **Sample names and preparation methods**: We thank the reviewer for noting this omission. The four SAXS profiles correspond to the NC'Am states prepared from $C_{400}O_{1818}C_{400}$ at different precursor solution concentrations (5 wt%, 10 wt%, 15 wt%, and 17 wt%, respectively). We have incorporated these details into the caption of Fig. 3b in the revised manuscript:

"…from $C_{400}O_{1818}C_{400}$ of different concentrations (5 wt%, 10 wt%, 15 wt%, and 17 wt%)"

(2) **Microdomain size as a parameter**: The reviewer raises a valid point. We agree that microdomain size and self-assembled morphology are additional structural parameters that can influence material properties. In Fig. 3b, the size variation arises from using the same polymer $C_{400}O_{1818}C_{400}$ with different precursor

solution concentration (5 wt%, 10 wt%, 15 wt% and 17 wt%), rather than from state transitions. For a given polymer composition (e.g., $C_{400}O_{1818}C_{400}$ at 17 wt%, the model system throughout the study), the microdomain size remains essentially constant across states as confirmed by the SAXS data (Fig. S6b → Fig. S11b). Therefore, within the 2×2 parameter matrix of our current work—focusing specifically on crystallinity and orientation—microdomain size does not constitute an independent variable. That said, we agree that a systematic investigation of how self-assembly structure (including domain size and morphology) modulates material properties would be highly valuable and worth pursuing in future studies. To explain this point, we have added the following text to the manuscript on **Page 9**, **Line 9**:

"…with radius and center-to-center distance of the micelles increase with the molecular weight of hydrophobic block and concentration of precursor polymer solution (Fig. 3b, S4-S6 → Fig. 3b, S9-S11, Table S4). Importantly, for the model system used for the rest of the study ($C_{400}O_{1818}C_{400}$ at 17 wt%), the microdomain size remains essentially constant across the hydrogel and dry states (Fig. S6b → Fig. S11b)."

(3) **TEM image of microdomains**: The spherical microdomains are formed by the PCL blocks. In the TEM image, the light-colored spherical regions correspond to PCL micellar structures resulting from the phase separation of hydrophobic PCL. The imaging was performed using EM stain: the hydrophilic PEO matrix is stained and appears dark, while the hydrophobic PCL cores are unstained and appear light. We added the following text to the manuscript on **Page 9**, **Line 4**:

"In the TEM images, the light-colored spherical domains correspond to the unstained PCL micelle cores, while the hydrophilic PEO matrix appears dark due to negative staining with uranyless EM stain."

Comment 1-3: *Fig. 4b presents the stress-strain curves of fine-tuned NCHC states with various necking and strain-hardening behaviors. Notably, the first three samples have similar parameters but quite different stress-strain curves. Does this mean that the mechanical response is very sensitive to a slight difference in A-block crystallinity or orientation? If so, the precise degree of crystallinity should be very important. It was only roughly introduced that crystallinity was analyzed from 1D WAXS plot. In addition, for all samples of Fig. 4b, the sum of PCL and PEO crystallinities is larger than 100%. It seems that the diffraction halo was determined for PCL and PEO separately to calculate individual crystallinity. However, Figs. S7b and S9a show that the characteristic diffraction peaks are located within close scattering vector range. How to determine the PCL and PEO amorphous haloes from the overlapped diffraction signals? The detailed analysis method can be provided.*

Response: Thank you for these insightful comments regarding Fig. 4b**.** The stress–strain curves shown in this figure correspond to NCHC prepared from NC'Am by applying trigger 6 (dehydration under strain) at different strain levels (strain = 0, 0.2, 0.4, 0.6, 0.75). In NC'Am (hydrogel state), the PEO chains are in a

highly hydrated and mobile state. Even under very small strain, dehydration can induce highly oriented crystallization. For example, the first stress–strain curve corresponds to the sample dehydrated at strain = 0 (original length). During drying, volume shrinkage generates internal stress, leading to highly oriented PEO crystallization (f_B = -0.38).

This phenomenon points to a subtle aspect of the system: while the four-parameter matrix ($\chi_{c,A}$, $\chi_{c,B}$, f_A, f_B) describes the crystalline architecture, chain orientation in the amorphous regions contributes additional structural variation across the NCHC series. Within these low strain ranges, the slight increase in applied strain during dehydration has limited effect on crystalline orientation ($f_B \approx$ -0.38 to -0.42, close to the theoretical limit of -0.5) but produces a progressive change in chain orientation in the amorphous regions, as detected by polarized FTIR (Fig. S25). As a result, samples with comparable matrix values can still exhibit substantially different stress-strain responses.

The following Fig. S25 was added to SI on **Page S29**:

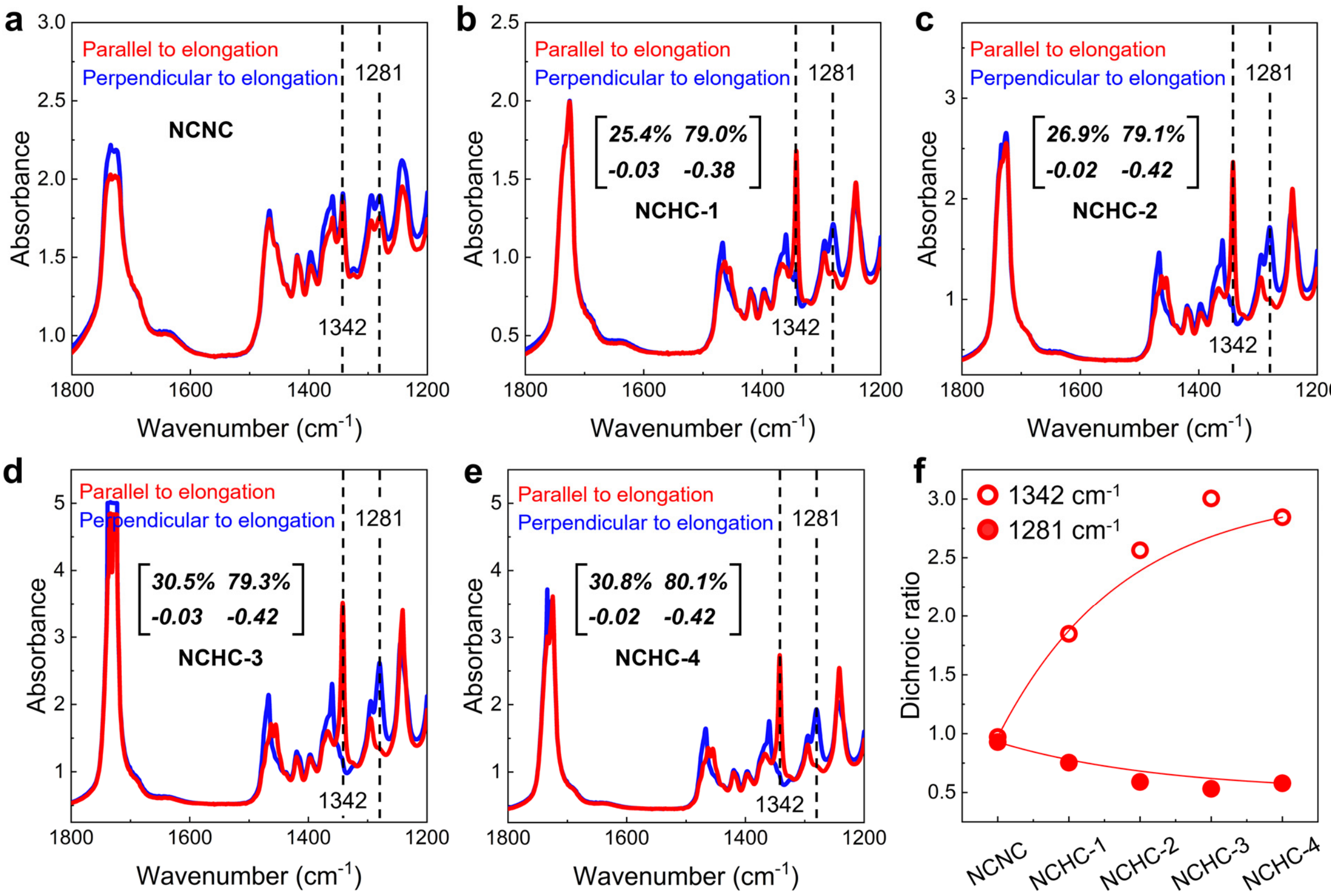

Fig. S25. Polarized FT-IR spectra for **(a)** NCNC and **(b-e)** NCHC. The red and blue lines represent electric vectors parallel and perpendicular to elongation direction. For isotropic state, band intensities of PEO at 1342 cm^{-1} and 1281 cm^{-1} are similar in both polarizations. **(f)** Chain orientation (Dichroic ratio) measured

by polarized FT-IR spectroscopy. Dichroic ratios of PEO bands at 1342 cm^{-1} and 1281 cm^{-1} (parallel band) and elongation varied significantly while the four parameters in matrix are similar.

The following text has been incorporated into manuscript on **Page 12, Line 12** and **Line 16**:

"By applying trigger 6 (dehydration under strain) to NC'Am at varying strain levels, a series of stable and continuous microstates can be achieved (e.g., NCHC and MCHC), leading to a tunable strain range of necking window based on the chain orientation across crystalline and amorphous regions (Fig. S25)…Notably, the four-parameter matrix ($\chi_{c,A}$, $\chi_{c,B}$, f_A, and f_B) describes the crystalline component of the network and does not fully capture chain orientation in the amorphous regions. When samples share comparable values across these four parameters, differences in amorphous chain orientation can still produce substantially different stress–strain responses, as seen across the curves in Fig. 4b."

Regarding the sum of crystallinity exceeding 100% in Fig. 4b, we apologize for the confusion. Here, the crystallinity values of PCL and PEO are weight-fraction crystallinities relative to their respective block masses, not the total polymer mass. Specifically, $\chi_{c,A}$ is the crystalline fraction of the PCL block only, and $\chi_{c,B}$ is the crystalline fraction of the PEO block only. When expressed relative to total polymer mass, their sum does not exceed 100%. The assignment of crystalline peaks in the 1D WAXS data is shown in Fig. S2a (→ Fig. S7a).

The following calculation method was added to the SI on **Page S52** to clarify this point:

For example, the crystallinity (%) of PEO was calculated by followed equation:

$$X_{c,PEO} = \frac{A_{PEO,crystalline}}{A_{PEO,crystalline} + A_{PCL,crystalline} + A_{amorphous}} \frac{1}{\omega_{PEO}}$$

ω_{PEO} indicates the mass fraction of PEO block, for $C_{400}O_{1818}C_{400}$, ω_{PEO} is 46.5%. $A_{PEO,\ crystalline}$ and $A_{PCL,\ crystalline}$ represent the integrated area of the PEO and PCL crystalline diffraction peak. $A_{amorphous}$ represents the integrated area of the amorphous haloes.

Comment 1-4: *There are four parameters in the parameter matrix to distinguish different states. It will make more sense to explain the reported properties by these parameters. The authors are suggested to explain the specific role of each parameter in determining properties of interest.*

Response: We appreciate the reviewer's suggestion. Please refer to our response to Comment 1-1, where we discuss the specific role of each parameter ($\chi_{c,A}$, $\chi_{c,B}$, f_A, f_B) in determining the material properties.

Comment 1-5: *The axis of Fig. S24 should be elongation ratio, rather than elongation-at-break ratio.*

Response: We are sorry for this error. The x-axis label has been corrected to "Elongation ratio ($\lambda = L/L_0$)" in the revised version (Fig. S24 → Fig. S27).

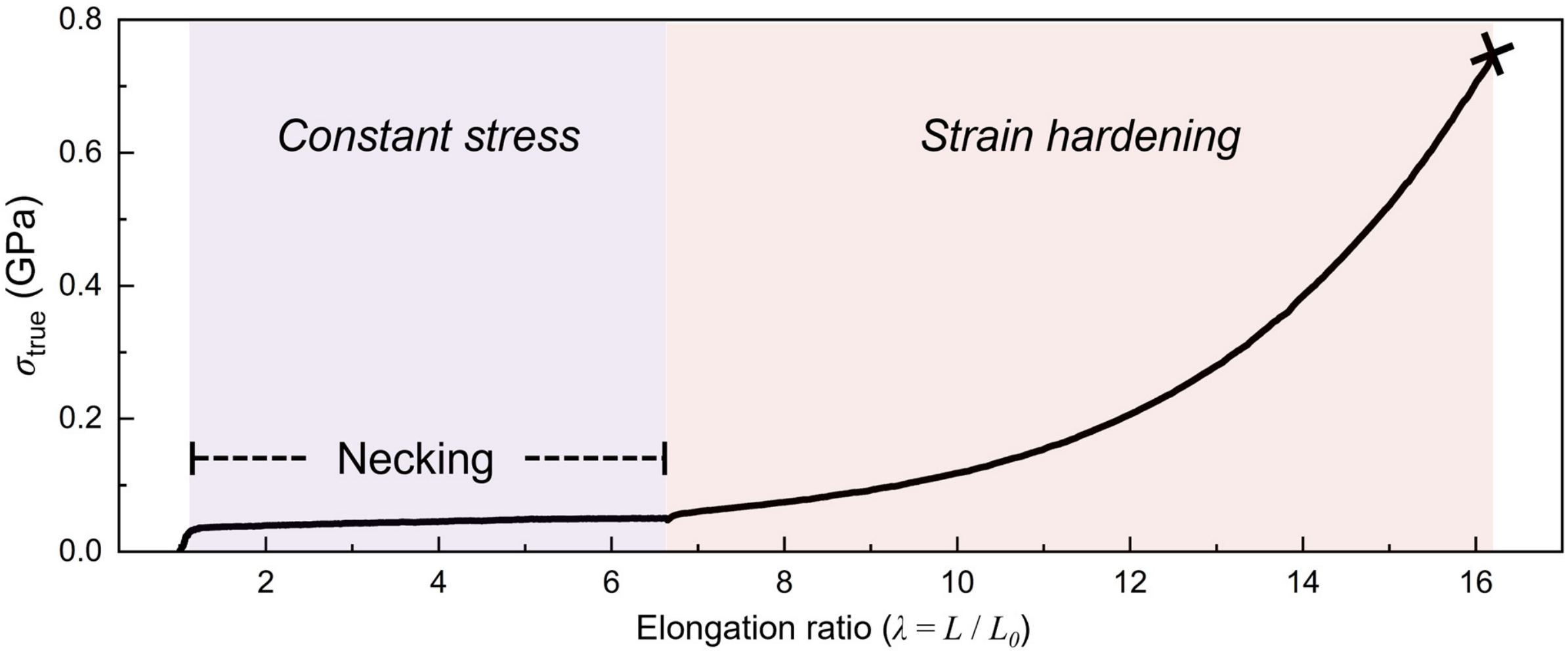

Fig. S24 (→ Fig. S27). True stress (σ_{true}) vs elongation ratio ($\lambda = L / L_0$) curves of NCNC state ($C_{400}O_{1818}C_{400}$).

Comment 1-6: *Fig. 4b inset shows the specimen that integrates states with and without necking behavior. Normally, necking appears and then propagates in two directions within sample. As necking requires the same stress for various positions, propagation apparently shows a constant stress, leading to a constant stress plateau in engineering stress-strain curve. After this propagation of local necking, the necked sample can be further deformed and gives the strain-hardening response. However, it is hard to understand why the so-called constant stress part was observed in the middle of two necking regions, see Fig. 4b. Does this mean that more than one necking process were trigged within the same sample? On the other hand, the necking strain that largely increased from 0.7 to 2.7 was emphasized by the authors. What is the physical meaning and cause of various necking strains?*

Response: The two questions regarding the necking mechanism were addressed below,

(1) On the constant-stress plateau between two necking regions in Fig. 4b

As described by the reviewer, we observed classic necking–propagation–hardening sequence in our system. In the specific case of Fig. 4b, the specimen shown in the inset is **an integrated sample to show that NCHC and HCHC can be incorporated into one fiber**: we applied Trigger 8 (cold strain) to only a portion of the NCNC sample, converting the strained region into HCHC while leaving the remainder as

NCNC.

We additionally note that, even in homogeneous NCNC specimens, we have occasionally observed the emergence of two or multiple necking sites within the same sample. This was attributed to material non-uniformity (local variations in microstructure, defects, density, or orientation), which can cause different positions to reach the necking criterion at slightly different strain levels. Because the propagation of these local necks occurs at comparable stress levels, they typically merge into a single continuous plateau on the macroscopic engineering stress–strain curve, rather than producing fully separated multiple plateaus.

We have included the following description in the caption of Fig. 4b on **Page 11**:

"Inserted left: a single fiber containing both NCNC and HCHC regions, prepared by applying trigger 8 (cold strain) to a portion of an NCNC sample; the strained region converts to HCHC, while the remainder stays as NCNC."

(2) **On the physical meaning and origin of varying necking strains**

The necking strain (ε_N) is defined as the total engineering strain accommodated during neck propagation before strain-hardening begins. Physically, it reflects the extent of structural reorganization (fibrillation, chain unfolding, and recrystallization) that occurs during necking. In our system, ε_N is controlled by chain orientation of the A- and B-block: lower chain orientation (closer to NCNC) provides more "structural room" for reorganization, yielding larger ε_N. Conversely, higher initial crystallinity and alignment (chain orientation) mean less reorganization is needed, resulting in smaller ε_N. This tunability of ε_N underlies the tunable impact energy absorption capacity, as demonstrated in Fig. 4g.

Comment 1-7: *Fig. 5b and 5d show tunable actuation stroke and velocity through parameter control of material states, respectively. When carefully examining these parameters, crystallinity values are quite close, while orientation changes clearly. Is it possible that orientation factor is the crucial parameter to determine actuation behaviors? Based on the results of Fig. 5d, it seems that the actuation stress is determined by block-A orientation factor* f_A.

Response: We appreciate the reviewer's careful observation. The orientation factors are indeed the primary determinants of actuation behavior, but the roles of f_A and f_B depend on the chosen stimulus.

Under heating, both blocks melt, disorient, and contribute to actuation. Under hydration (Fig. 5d), only block B (PEO) responds to water. Block A (PCL) is hydrophobic and not directly affected. Hydration dissolves the oriented PEO crystallites and relaxes the oriented PEO chains in the amorphous regions, and this loss of PEO orientation provides the driving force for contraction. Block B is therefore the actuating

component under hydration, even though f_A in Fig. 5d shows a larger numerical variation. As described in our response to Comment 1-3, the four-parameter matrix describes crystalline orientation but not chain orientation in the amorphous regions. Applying trigger 6 (dehydration under strain) to MCAm, f_B saturates even at low applied strain while chain orientation in the amorphous PEO regions continues to develop with increasing strain. The larger variation of f_A relative to f_B therefore reflects this saturation of f_B rather than a dominant role of block A.

To clarify this mechanism, we added the following text in the revised manuscript on **Page 15**, **Line 17**:

"Across the parameter matrix, actuation is governed primarily by block-B orientation under hydration and by both blocks under heating. Under hydration, the driving force is the loss of PEO orientation. Although f_B is nearly saturated, chain orientation in the amorphous PEO regions continues to develop with strain and contributes to the actuation response."

Comment 1-8: *In many mechanical curves (for example Fig. S20), a stress drop appeared at the end of stress plateau but a rapid stress increase appeared before strain hardening. What is this peculiar stress change?*

Response: Thank you for your careful observation. A stress drop is indeed present at the transition from the necking plateau to strain hardening in samples with a large necking strain (e.g. $\varepsilon_N > 1$), while it becomes minor or undetectable in samples with short or absent plateaus (e.g., NCHC-4 with $\varepsilon_N \approx 0.5$, NCHC-5 and MCHC, where necking is not clearly resolved, Fig. S22 → Fig. S24).

While similar transient features have been reported in cold drawing of other semicrystalline polymers (*Macromolecules* **2007,** *40,* 9664), they are typically much less pronounced, and the underlying mechanism remains unsettled. A plausible interpretation is that, during the plateau, the dry-state crystalline framework of the NCHC material is progressively broken down as drawing proceeds toward the HCHC configuration. At the transition to strain hardening, this original framework has been largely disrupted while the load-bearing aligned-chain network of the fully drawn state has not yet been fully built up. This brief structural gap may allow stress to relax momentarily before the new network forms and engages. A systematic study using in situ structural characterization across the transition would be needed in future work to clarify the molecular mechanism.

Reviewer #2

In this manuscript, the authors report a multistate material circuit (MMC) system based on PCL–PEO–PCL triblock copolymers. The triblock copolymers exhibit eight distinct states depending on preparation conditions such as water content, temperature, and mechanical stress (strain), which serve as controlling parameters, with each state showing different mechanical properties. In addition, switching between these states can be controlled by externally added stimuli, such as humidity, temperature, and stress. The authors conducted extensive experiments and carefully investigated the structural characteristics, mechanical properties, and switching behavior of the MMC system. Furthermore, a number of practical demonstrations that exploit the multimodal and switchable properties of the system are presented. Considering these points, the manuscript is likely to be of interest to the broad readership of Nature Communications. However, some details of the experimental conditions are missing, which may hinder the reproducibility of results. Therefore, the following points should be addressed.

Comment 2-1: *Since the mechanical properties of polymers were highly affected by the water content and temperature, the authors should provide the quantitative experimental conditions, including humidity and room temperature. In addition, it would be important to show the water content of polymers in each state and to clarify the dehydrated state, including the conditions for its preparation.*

Response: We agree that providing precise environmental conditions is important for reproducibility. Following your suggestion, we have added TGA measurements, and revised manuscript and SI accordingly:

(1) On **Page S52**, we added in the method section "All mechanical tests were conducted at room temperature (25 ± 1°C) and ambient relative humidity (65 ± 10% RH)."

(2) Water content for each hydrogel state is provided in Fig. S13 (NC'Am, NCAm, and MCAm), which is around 80 wt% to 90 wt%. For dry states, the dehydration was performed by air-drying at room temperature (25 ± 1°C) until constant weight was achieved (typically 8-10 min for fiber samples), and the residual water content of different samples were measured by thermogravimetric analysis (TGA). We have included the related results in the SI on **Page S23**:

Since water content significantly affects the mechanical properties, we determined the residual water content of samples in the dry state by TGA using the following equation:

$$\text{Water content} = \frac{Mass_{30°C} - Mass_{150°C}}{Mass_{30°C}} \times 100\%$$

$Mass_{30°C}$ indicates the mass fraction of dry state material at 30°C. $Mass_{150°C}$ indicates the mass fraction of dry state material when the temperature reaches 150°C.

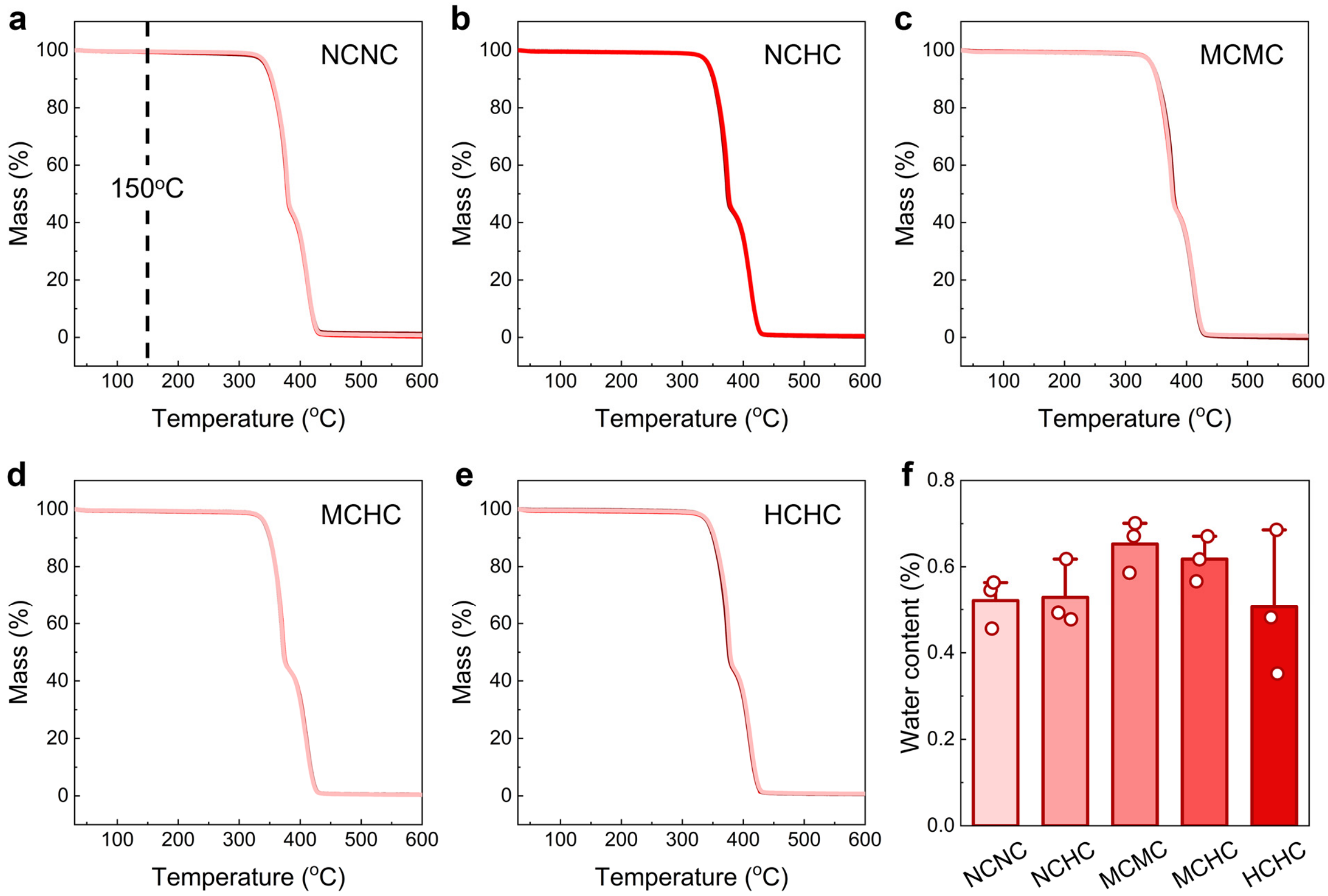

Fig. S18. Water content of dry states material. Thermogravimetric analysis (TGA) curve of NCNC **(a)**, NCHC **(b)**, MCMC **(c)**, MCHC **(d)** and HCHC **(e)**. Mass loss was recorded under N_2 at a heating rate of 10 °C·min^{-1} from 30°C to 600°C. **(f)** Water content of NCNC, NCHC, MCMC, MCHC and HCHC, determined from TGA curves by accounting for mass loss due to water removal in the 30–150°C temperature range.

Comment 2-2: *If the dry material transforms into a hydrogel, a significant volume change is expected. Could the authors comment on the extent of the volume change between the hydrated and dehydrated states?*

Response: Thank you for your insightful question. We have measured the volumetric change accompanying the dry-to-hydrogel transition by tracking the same fiber across states. The cross-sectional area A was measured by optical microscopy and the length L by direct imaging, giving the fiber volume $V_e = A \cdot L$ in each state. Across the compositions tested (NCNC, NCHC, MCMC, MCHC, HCHC), ε_V falls in

the range of ~170% to 800%, with the specific value depending on initial dry state and the trigger pathway used.

Trigger 2 involves heating the dry fiber to 60°C in water—above the melting temperature of the PCL crystalline domains—followed by cooling to room temperature while immersed; this transiently releases the crystalline physical crosslinks, allowing the network to swell extensively before the crosslinks reform on cooling, locking in a high-water content. Trigger 4 consists of direct hydration at room temperature, during which the crystalline crosslinks remain intact and constrain swelling. Consequently, fibers reaching the hydrogel state via Trigger 2 (NC′Am) exhibit substantially larger ε_V (~560%–800%) than those reaching it via Trigger 4 (NCAm, MCAm; ~170%–330%), as shown in Fig. S17.

We have included the related results and discussion in the SI on **Page S22**:

To determine the volumetric change between hydrogel state and dry state, the same fiber was brought into different states using the trigger protocol shown in Fig. 2 of the manuscript. The cross-sectional area A of the fiber in each state was measured by optical microscopy, and the fiber length L was recorded; from these measurements the volume V for each state was calculated by followed equation:

$$V_{\text{material state}} = A \times L$$

The volumetric strain associated with the transition between states was calculated using the following equation:

$$\varepsilon_V = \frac{V_{\text{hydrogel state}} - V_{\text{dry state}}}{V_{\text{dry state}}} \times 100\%$$

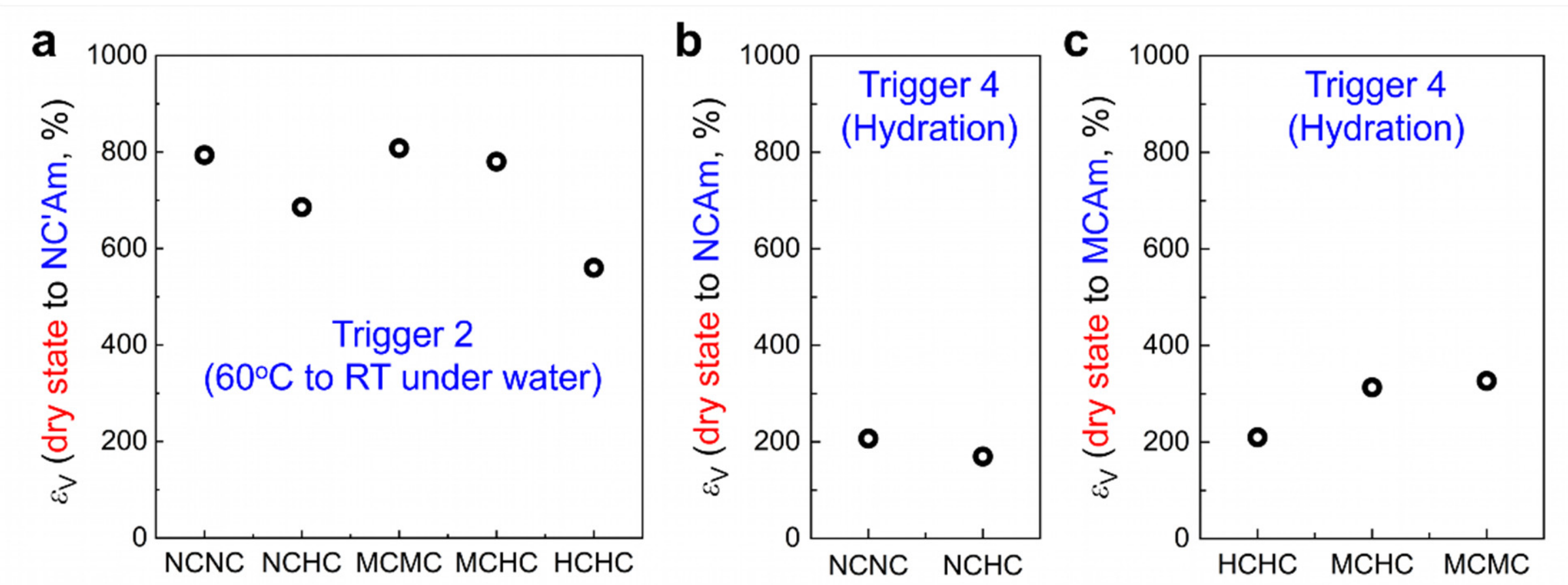

Fig. S17. Volumetric strain when dry state transforms into hydrogel state. (a) NCNC, NCHC, MCMC,

MCHC and HCHC transform into NC’Am through 60°C to RT under water (Trigger 2). **(b)** NCNC and NCHC transform into NCAm through hydration (Trigger 4). **(c)** HCHC, MCHC and MCMC transform into MCAm through hydration (Trigger 4).

ε_V depends strongly on the trigger pathway. Fibers transformed via Trigger 2 (heating to 60°C in water, followed by cooling to room temperature under water) reach the NC′Am state with ε_V in the range of ~560%–800%, whereas the same compositions transformed via Trigger 4 (direct hydration at room temperature) reach the NCAm or MCAm state with ε_V of ~170%–330%. This pathway-dependence reflects the state of the crystalline physical crosslinks during hydration. In Trigger 2, heating above the melting temperature of PCL crystalline domains transiently releases these crosslinks, allowing the network to swell more extensively in water before the crosslinks reform upon cooling. In Trigger 4, the crystalline crosslinks remain intact throughout hydration and constrain the extent of swelling. The hydrogel states reached by the two pathways (NC′Am vs NCAm/MCAm) therefore correspond to distinct network configurations.

Comment 2-3: *It would be helpful for readers to understand the clarification of eight states, if the authors could emphasize the reason why they included the series of materials with medium-aligned crystalline (MCAm, MCMC, and MCHC; f = -0.1 to -0.35) into the circuit as representative key states, because they seemed to be intermediate states during the transitions of NC to HC states.*

Response: Thank you for the question. The MC state is not a simple transient intermediate along the NC and HC transition, but rather the maximum PCL crystalline alignment attainable in the hydrogel state, set by the geometric constraint of PCL crystallites within the swollen PEO matrix. This upper bound is supported by two independent observations: (i) when NCAm is cold strained under water (Trigger 3), the orientation parameter saturates at $f \approx -0.35$; (ii) when highly aligned dry states such as HCHC are returned to the hydrogel state by Trigger 4, hydration at room temperature (preserving PCL alignment without thermal melting), the orientation relaxes to the same value. Because $f \approx -0.35$ is approached from both the NC and HC sides, we think MC is a structurally important node of the circuit rather than a kinetic stop.

To clarify this point, the following text was added to the manuscript on **Page 7**, **Line 5**:

“This f value, which defines the MC state in our system, is the upper bound of PCL alignment attainable in the hydrogel state, set by the constrained mobility of PCL crystallites within the swollen PEO matrix.”

Comment 2-4: *Under what conditions can the AmAm state be obtained?*

Response: The AmAm state is obtained by heating the sample above ~60°C, either in water or in air. Under this condition, the PCL block melts and the PEO block become molten or dissolved, so both blocks reach

the amorphous (Am) state. AmAm functions as an intermediate state that resets the system: once the heating trigger is removed, it spontaneously evolves into one of the eight stable states depending on the surrounding medium (e.g., NCNC in air, NC′Am in water), as shown in Fig. 2.

Comment 2-5: *In pages 14–15, numerous applications are demonstrated without sufficient explanation. Even in the Supplementary Material, some experiments are described only in the figure captions, and the experimental conditions are unclear. For example, it is difficult to understand how the "hydration" of the fibers shown in Figs. S36–S38 and S42–S43 was carried out. In addition, the purpose of Fig. S42 is not clear. Where are the experimental results corresponding to Fig. S42e?*

Response: We apologize for not providing enough details regarding experimental conditions. Significance and advantages of the demonstrations on **Page 14-15** were added (see more details in our response to Comment 2-6). The following explanations on experimental conditions were also added,

Hydration protocol: In all cases the wetting medium is DI water at room temperature; the only differences across experiments are how the water is delivered, which is dictated by the test geometry.

- **Bath immersion** is used when one fiber end must remain free to record displacement (actuation stroke / velocity, Fig. S36-S38, S45 → Fig. S39-S41, S48).
- **Mist spray** is used when both ends are clamped on a load frame and immersion is not possible (isometric force, Fig. 5d, S42d → Fig. 5d, S45d).
- **Contact with a pre-wetted surface** is used in device-level demonstrations where the fiber or fabric must operate in conformal contact with a wet substrate (Fig. 5f, S43 → Fig. 5f, S46).

The relevant figure captions have been updated to indicate which configuration was used.

For hydration-driven actuation of fibers (Figs. S36-S38 → Figs. S39-S41), we added the following text:

"The oriented fiber was placed vertically, with the upper end clamped to an iron stand and the lower end attached to a needle marked with a reference point. A graduated cylinder filled with deionized water was then raised from below so that the entire fiber was submerged within ~1 s (Supplementary Video 6). Upon hydration, dissolution of the oriented PEO segments drives contraction. The process was video-recorded, and the motion was analyzed using Tracker 5.1.x (The Open-Source Physics Project) to extract the actuation stroke and velocity. The oriented fiber was clamped at both ends in a load frame, and deionized water was then applied until the fiber was fully wetted. The force generated upon hydration (applied through mist spray) was recorded, and the actuation stress was obtained by dividing this force by the initial cross-sectional area of the fiber."

Tension-reducing tapes (Fig. S42 → Fig. S45): In Fig. S42 (→ Fig. S45), we demonstrate an MMC-based intelligent tension-relief patch designed for non-invasive wound closure. The underlying mechanism is similar to that of the intelligent surgical suture shown in Fig. S41 (→ Fig. S44), where blood exudate stimulates the fibers to generate contractile force and close the wound. Unlike sutures, however, this patch enables non-invasive closure.

The preparation process is as follows: Five NCNC fibers (each 1 cm in length) were fixed at both ends onto two adhesive patches using glue, as shown in Fig. S42b (→ Fig. S45b). During cold drawing, NCNC fibers exhibit tunable high extensibility (Fig. S42c → Fig. S45c), which allows flexible adjustment according to wound size. Depending on the requirement, part or all of the NCNC fibers can be processed into HCHC fibers. Upon hydration, five HCHC fibers can generate a contractile force of 0.22 N (Fig. S42d → Fig. S45d). By adjusting the number of fibers, the generated force can be tailored to match the modulus of the surrounding tissue, thereby achieving an ideal closure effect.

Regarding Fig. S42e (→ Fig. S45e): Fig. S42e (→ Fig. S45e) is a mechanistic schematic of the three-stage closure process; it is not a separate experiment. The experimental evidence supporting each stage is provided: Stage 1, high modulus for protection (Fig. 2, 3h, S15 → Fig. 2, 3h, S19); Stage 2, hydration-triggered contraction (Fig. 2 HCHC to MCAm via trigger 4, Fig. S42d → Fig. S45d), Stage 3, dehydration-driven re-stiffening (Fig. 2 MCAm to MCMC via trigger 5). We have clarified this in the revised caption of Fig. S42e (→ Fig. S45e).

For **moisture-responsive fabric** (Fig. S43 → Fig. S46), we added the following text,

"As a proof-of-concept, we demonstrate an MMC-based smart fabric in which HCHC fibers were sewn onto a commodity textile. To mimic perspiration, water was sprayed onto a flat surface to form a thin liquid layer, and the fabric was then placed in contact with the wetted surface. Upon hydration, the HCHC fibers contracted and lifted the textile locally, transforming the initially flat fabric into a corrugated structure with five regularly spaced ridges aligned with the embedded fibers (Fig. S43b, c → Fig. S46b, c). Open channels formed in the valleys between adjacent ridges, and contact with the wetted surface was confined to the ridge tops, reducing the fabric–surface contact area. The resulting ventilation channels between the fabric and the skin would be expected to enhance local airflow and heat dissipation."

We also revised captions for Fig. S43 (→ Fig. S46),

Fig. S43 (→ Fig. S46). Proof-of-concept demonstration of moisture-responsive fabric. (a) Schematic illustration: in the dry state the fabric lies flat against the body, while upon wetting (e.g., by perspiration) the fabric forms localized ridges, opening ventilation channels between the fabric and the skin (*14*). **(b)**

Photographs of the fabric with sewn-in HCHC fibers in the dry state (top) and after hydration (bottom), showing the formation of a corrugated structure. **(c)** Time-lapse images of the fabric placed on a wetted surface: (1) initial flat state, (2) after surface wetting, (3) onset of ridge formation, (4) fully developed corrugation with five ridges aligned with the embedded HCHC fibers. Contact with a pre-wetted surface was employed as the hydration method.

These clarifications above have been added to the revised supplementary information to ensure that the purpose and mechanism of Figs. S36-S38 (→ Figs. S39-S41) and S42-S43 (→ S45-S46) are clearly understood.

Comment 2-6: *The demonstration section should be reorganized so that readers can clearly understand the significance of each demonstration, as well as what novel properties of the MMC materials offer advantages over previously reported stimuli-responsive materials.*

Response: As suggested, we have reorganized the demonstration section. We split the section into two paragraphs: biomedical applications (sutures and tension-reducing tapes) and non-biomedical applications (sweat-triggered textile and rotational engine). We also added explanations to clarify the significance of each demonstration. The major additions are as follows:

In the opening of the section (**Page 15**): "The MMC platform supports applications — biomedical devices, smart fabrics, and micro-engines — that conventional stimuli-responsive materials typically handle with separate systems."

In the suture and tape paragraph (**Page 15**): "The yarn modulus is tunable across the soft-tissue range, so contraction transfers less strain to the wound margin (Fig. S42 → Fig. S45)."

In the textile paragraph (**Page 16**): "Actuation is driven directly by the perspiration that signals the need for cooling, providing on-demand ventilation without external power."

To emphasize advantages over previously reported materials, we added the following summary at the end of the section (**Page 16**): "Across these demonstrations, the actuating element is a single, unfilled material without chemical crosslinking, simplifying fabrication and end-of-life processing relative to composite or crosslinked materials."

Comment 2-7*: The authors should rearrange the Supplementary Figures in the order they appear in the manuscript.*

Response: As suggested, we have rearranged the Supplementary Figures to follow the order in which they

are cited in the main manuscript.

Reviewer #3

We acknowledge Reviewer #3's participation as a co-reviewer in the Nature Communications Early Career Researcher initiative. No specific comments were raised by Reviewer #3.

Reviewer #4

The article by Wei et al. is a thorough analysis of the mechanical properties of a triblock copolymer under various states of hydration and crystallinity. The introduction and results need to do a better job comparing the current materials to the literature. Strain-induced crystallization is an old phenomenon, and the mechanical properties of a material are obviously dependent on its state of hydration. As currently written, it is not clear how these results are surprising or how the purported "multistate materials circuits" are unique relative to what is already known about block copolymer materials. The work is certainly comprehensive, thorough, and worthy of publishing somewhere.

Response: Thank you for acknowledging the thoroughness of the work and for raising the question of novelty directly. We agree that strain-induced crystallization and hydration effect are individually well-known phenomena. The contribution of this work, however, lies not in either phenomenon alone, but in their integration into a framework with quantitatively defined states and explicit transition pathways.

Although double crystalline block copolymers have been extensively studied across various aspects, a unified framework connecting these phenomena within a single parameter space has not been reported. Here, we organize these states into a 2×2 parameter space ($\chi_{c,A}$, $\chi_{c,B}$, f_A, f_B) with defined transition pathways. This lets us read structure–property relationships off the framework rather than trying to rationalize them case by case.

Several questions raised in this review process — for example, on parameter coupling — were resolved by reading them within the MMC framework. The reviewer asked whether χ and f can be tuned independently or are inherently coupled (Comment 1-1). The 2×2 organization reveals that some triggers decouple parameters cleanly, others couple them within or across blocks, and still others fall in between—regimes that are easily overlooked without a structured state space. By labeling each trigger according to which

parameters it moves, the framework replaces the either/or answer with a trigger-by-trigger description of how parameters couple.

Beyond conceptual organization, the framework also expands accessible mechanical performance range for this polymer family. PCL-PEO-PCL is valued for biocompatibility but has generally shown limited mechanical performance on its own (*Macromolecules, 2005, 38, 5260; Macromolecules, 2011, 44, 6629; European Polymer Journal, 2023, 201, 112526*). Without chemical modification or additives, we processed this single composition into states covering a modulus range of 0.1 MPa to 0.91 GPa, with a toughness of 445 MJ/m³, a true strength of 738 MPa, and 94% impact force damping. We are not aware of a previous block copolymer system that reaches this combined range. Existing modulus-switching materials (Table S10, Fig. S21 → Fig. S16) achieve comparable or larger switching ratios but at substantially lower mechanical performance.

In addition to revisions addressing the comments and questions listed above, we further modified the introduction to emphasize the novelty of the work and the benefits of the MMC framework.

On **Page 2**, we added the following sentence to emphasize that crystalline block copolymers are an established route for state-tunable materials, and positions our system within this established line of work,

"…creating single-material systems with multiple states that can be used stably at room temperature remains a significant challenge(*15, 16*). One promising route lies in crystalline block copolymers, where microphase-separated crystalline domains can be tuned in crystallinity and orientation as structural handles."

In response to your point that strain-induced crystallization and hydration effects are individually well-known, and your question about how the purported "multistate materials circuits" are unique, we added the following sentence on **Page 2**:

"While the crystallization behaviors and mechanisms of such systems, including double crystalline block copolymers, have been extensively studied, their states and transitions have not been systematically organized within a unified parameter space."

On **Page 3**, we added,

"…enabling versatile functionalities like dramatic modulus change reminiscent of sea cucumbers, muscle-like programmable actuation, and highly effective impact damping. The combination of continuous transitions and state quantification within a unified MMC framework allows on-demand control over material structure and properties."

In response to your question about how these results are surprising, on **Page 3** we added,

“We show that the MMC system provides on-demand control over a broad spectrum of material properties and functionalities, which can be achieved using the same polymer to reduce chemical complexity. Structure control alone — without chemical modification or additives — provided access to mechanical behaviors ranging from soft and deformable to strong and tough from a single composition.”

Table R1. Supplementary figure numbers before and after reordering.

Before reordered	After reordered		Before reordered	After reordered
Fig. S1	Fig. S1		Fig. S27	Fig. S30
Fig. S2	Fig. S7		Fig. S28	Fig. S31
Fig. S3	Fig. S8		Fig. S29	Fig. S32
Fig. S4	Fig. S9		Fig. S30	Fig. S33
Fig. S5	Fig. S10		Fig. S31	Fig. S34
Fig. S6	Fig. S11		Fig. S32	Fig. S35
Fig. S7	Fig. S2		Fig. S33	Fig. S36
Fig. S8	Fig. S3		Fig. S34	Fig. S37
Fig. S9	Fig. S4		Fig. S35	Fig. S38
Fig. S10	Fig. S5		Fig. S36	Fig. S39
Fig. S11	Fig. S6		Fig. S37	Fig. S40
Fig. S12	Fig. S12		Fig. S38	Fig. S41
Fig. S13	Fig. S13		Fig. S39	Fig. S42
Fig. S14	Fig. S14		Fig. S40	Fig. S43
Fig. S15	Fig. S19		Fig. S41	Fig. S44
Fig. S16	Fig. S20		Fig. S42	Fig. S45

Fig. S17	Fig. S21		Fig. S43	Fig. S46
Fig. S18	Fig. S22		Fig. S44	Fig. S47
Fig. S19	Fig. S23		Fig. S45	Fig. S48
Fig. S20	Fig. S15		Fig. S46	Fig. S49
Fig. S21	Fig. S16		Fig. S47	Fig. S50
Fig. S22	Fig. S24		Fig. S48	Fig. S51
Fig. S23	Fig. S26		Fig. S49	Fig. S52
Fig. S24	Fig. S27		Newly added	Fig. S17
Fig. S25	Fig. S28		Newly added	Fig. S18
Fig. S26	Fig. S29		Newly added	Fig. S25

Response to Reviewers' Comments

Reviewer #1

All of my previous concerns have been properly addressed and the manuscript has been revised. The current manuscript could be considered to be published in Nature Communications.

Reviewer #2

The authors have responded sincerely and thoroughly to the concerns raised by this reviewer. They have substantially revised the manuscript and provided additional data and discussion to support their conclusions. Therefore, I recommend this manuscript for publication in Nature Communications.

Reviewer #3

I co-reviewed this manuscript with one of the reviewers who provided the listed reports. This is part of the Nature Communications initiative to facilitate training in peer review and to provide appropriate recognition for Early Career Researchers who co-review manuscripts.

Response: We sincerely thank all the reviewers for their careful evaluation of our revised manuscript and for their positive assessments. We are grateful that the reviewers consider their previous concerns to have been adequately addressed and support the publication of our manuscript in Nature Communications.
