# Supplementary material for: Block copolymer multistate material circuit
Source: Nat Commun. 2026 Oct 6;17:11393. doi: 10.1038/s41467-026-78278-2 (PMC13736946; doi:10.1038/s41467-026-78278-2)
Supplement: Supplementary file 3 [file 41467_2026_78278_MOESM1_ESM.pdf]

Supplementary Information

Block copolymer multistate material circuit

Hongxin Wei[1, 2], Lingmin Lan[1, 2], Li Luo[1, 2], Ziqi Liu[1, 2], Changxing Ji[1, 2], Diyuan Zheng[1, 2], Jie Chen[1, 2], Robert J. Hickey[3, 4], Chao Lang[1, 2]*

1. South China Advanced Institute for Soft Matter Science and Technology, State Key Laboratory of Advanced Papermaking and Paper-based Materials, School of Emergent Soft Matter, South China University of Technology, Guangzhou 510640, China.

2. Guangdong Provincial Key Laboratory of Functional and Intelligent Hybrid Materials and Devices, Guangdong Basic Research Center of Excellence for Energy and Information Polymer Materials, South China University of Technology, Guangzhou 510640, China.

3. Materials Science and Engineering, The Pennsylvania State University, University Park, PA, USA

4. Materials Research Institute, The Pennsylvania State University, University Park, PA, USA

*Corresponding author: langchao@scut.edu.cn

Table of Contents

1. Synthesis of block copolymers 3
2. Triggers and transitions of MMC 4
    Selectivity and coupling among the four order parameters 7
    Microstates between NC'Am and MCAm 8
    Microstates between NCHC and HCHC 8
    Microstates between MCMC and HCHC 10
3. Key states and microstates of MMC 12
    Structure analysis of the eight key states 12
4. Structure and mechanical properties of hydrogel states 14
    Structure analysis of NC'Am 14
    Mechanical properties of hydrogel states 15
5. Switch between hydrogel and dry states 18
6. Mechanical properties of dry states 23
7 Necking and impact damping characterizations 28
    Fine-tuning necking behaviors 28
    POM and SEM characterization of necking deformation 32
    Impact damping performance of MMC 33
8. Actuation behaviors of MMC 37
    Thermo-responsive behaviors of PEO and PCL crystal 37
    Tunable actuation triggered by heat 38
    Tunable actuation triggered by hydration 39
9. Applications of MMC-based actuators 42
    Self-closure surgical suture 42
    Blood-triggered tension-reduce tape 43
    Moisture-responsive fabrics 44
    Rotational microengine 45
10. Recyclability of MMC 47
11. Biocompatibility of MMC 47
    Hemolytic activity characterization 49
    Cytotoxicity characterization 49

1. Synthesis of block copolymers

Triblock copolymer poly(ε-caprolactone)-b-poly(ethylene oxide)-b-poly(ε-caprolactone), COC, was synthesized via ring-opening polymerization (ROP).

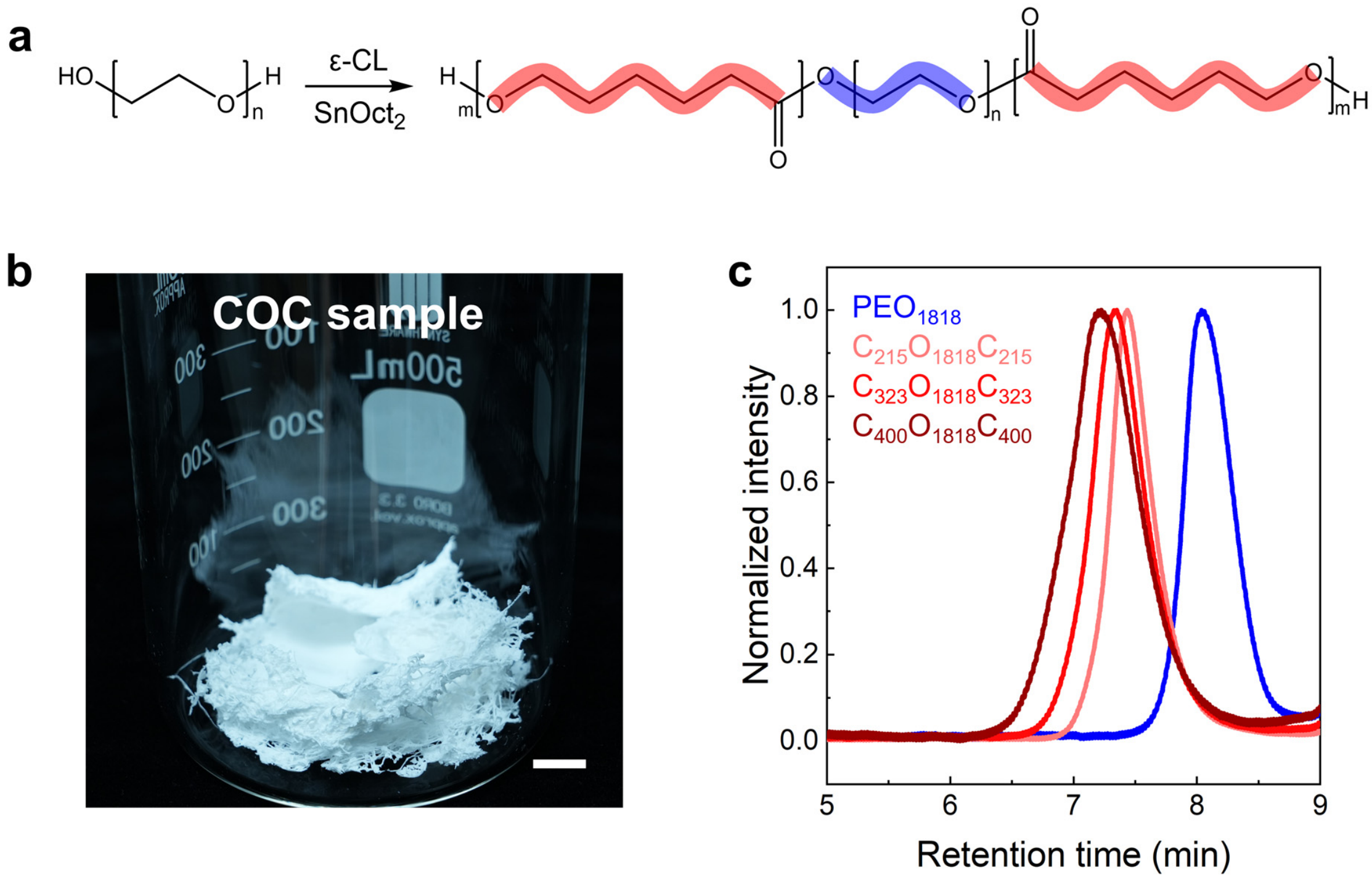

Fig. S1. (a) Synthetic scheme of COC. **(b)** Sample of COC polymer. **(c)** SEC traces of PEO and COC.

Table S1. Polymer molecular weight, dispersity, and block ratios of COC.

COC block copolymer[a]	M_n, total[b] (kg mol^{-1})	$Đ$[c]	ω_c[d] (wt%)	f_c[d] (%)
$C_{215}O_{1818}C_{215}$	129	1.18	38.0	38.2
$C_{323}O_{1818}C_{323}$	154	1.23	48.0	47.7
$C_{400}O_{1818}C_{400}$	171	1.34	53.2	53.2

[a] Number sequence of $C_mO_nC_m$ stands for COC with degree of polymerization of each block is m, n, and m kg mol^{-1}, respectively. [b] Total number-average molecular weight was determined by ^{1}H NMR. [c] Dispersity index (M_w / M_n) was determined using SEC. [d] Weight fraction and volume fraction of PCL block were calculated using the ^{1}H NMR data. Polymer density values were obtained from Sigma-Aldrich with PEO as 1.13 g mL^{-1} and PCL as 1.145 g mL^{-1} at 25°C.

2. Triggers and transitions of MMC

Table S2. 31 different transition pathways based on 8 triggering methods

Reversible transitions					
**State #1**	**State #2**	**Direction**	**Transition type[1]**	**Transition time[2]**	**Trigger method[3]**
NC'Am	NCAm	**#1 → #2**	Discrete	5 min	-4°C to RT under water
		**#2 → #1**	Discrete	2 s	60°C to RT under water
	MCAm	**#1 → #2**	**Continuous**	2 min	Cold strain under water
		**#2 → #1**	Discrete	2 s	60°C to RT under water
	NCNC	**#1 → #2**	Discrete	8 min	Dehydration
		**#2 → #1**	Discrete	2 s	60°C to RT under water
	NCHC	**#1 → #2**	**Continuous**	5 min	Dehydration under strain
		**#2 → #1**	Discrete	2 s	60°C to RT under water
	MCHC	**#1 → #2**	**Continuous**	5 min	Dehydration under strain
		**#2 → #1**	Discrete	2 s	60°C to RT under water
	HCHC	**#1 → #2**	**Continuous**	2 min	Dehydration under strain
		**#2 → #1**	Discrete	2 s	60°C to RT under water
NCAm	NCNC	**#1 → #2**	Discrete	5 min	Dehydration
		**#2 → #1**	Discrete	5 s	Hydration

NCAm	NCHC	**#1 → #2**	**Continuous**	4.5 min	Dehydration under strain
		**#2 → #1**	Discrete	2 s	Hydration
MCAm	MCMC	**#1 → #2**	Discrete	2 min	Dehydration
		**#2 → #1**	Discrete	4 s	Hydration
	MCHC	**#1 → #2**	**Continuous**	3 min	Dehydration under strain
		**#2 → #1**	Discrete	2 s	Hydration
	HCHC	**#1 → #2**	**Continuous**	2 min	Dehydration under strain
		**#2 → #1**	Discrete	2 s	Hydration
NCNC	HCHC	**#1 → #2**	Discrete	2 s	Cold strain
		**#2 → #1**	Discrete	2 s	60ºC to RT

Irreversible transitions

**Initial State**	**End State**	**Transition type[1]**	**Transition time**	**Trigger method[3]**
NCHC	NCNC	Discrete	2 s	60ºC to RT
	HCHC	Discrete	2 s	Cold strain
MCMC	NC’Am	Discrete	2 s	60°C to RT under water
	NCNC	Discrete	2 s	60ºC to RT
	HCHC	Discrete	2 s	Cold strain
MCHC	NCNC	Discrete	2 s	60ºC to RT
	HCHC	Discrete	2 s	Cold strain

[1]Discrete transition indicates a single initial state will transform into one end state under trigger. continuous transition involves a series of intermediate microstates by tuning parameters of the triggering methods. NC'Am was the initial state of the system, which was prepared using the rapid-injection method.(*1*) A typical procedure is to inject 2.5 mL 17 wt% THF solution of COC into 500 mL deionized water (speed of 0.5 mL/min), followed by dialysis against water to remove residual THF. [2]Transition time for each pathway was determined from samples prepared from NC'Am fiber with the lateral dimension of 120 μm×260 μm and a length of 1 cm. [3]Detailes of the eight triggering methods are as follows:

Trigger 1 (-4°C to RT under water): NC'Am was cooled down to freeze and then brought back to room temperature to afford NCAm.

Trigger 2 (60°C to RT under water): The material was heated to 60°C under water, followed by cooling to room temperature. Seven different transition pathways exist: NCAm to NC'Am, MCAm to NC'Am, NCNC to NC'Am, NCHC to NC'Am, MCMC to NC'Am, MCHC to NC'Am, HCHC to NC'Am.

Trigger 3 (Cold strain under water): NC'Am was strained under water at room temperature with a strain rate of 1/min to afford MCAm.

Trigger 4 (Hydration): Dry states were hydrated at room temperature to afford hydrogel states. Five transition pathways were achieved: NCNC to NCAm, NCHC to NCAm, HCHC to MCAm, MCHC to MCAm, MCMC to MCAm.

Trigger 5 (Dehydration): Hydrogel states were dehydrated without applied strain at room temperature to afford dry states. Three transition pathways can be achieved: NCAm to NCNC, NC'Am to NCNC, MCAm to MCMC.

Trigger 6 (Dehydration under strain): Hydrogel states were dehydrated under applied strain, which will lead to different dry states. Six transition pathways were achieved: NC'Am to NCHC, NC'Am to MCHC, NC'Am to HCHC, NCAm to NCHC, MCAm to MCHC, MCAm to HCHC.

Trigger 7 (60°C to RT): The material was heated to 60°C and then brought back to room temperature. Four transition pathways can be achieved: NCHC to NCNC, MCMC to NCNC, MCHC to NCNC, HCHC to NCNC.

Trigger 8 (Cold strain): The material was cold drawn at a strain rate of 1/min at room temperature. Four transition pathways can be achieved: NCNC to HCHC, NCHC to HCHC, MCMC to HCHC, MCHC to HCHC.

Selectivity and coupling among the four order parameters

The four structural parameters defining the MMC states—$\chi_{c,A}$, $\chi_{c,B}$, f_A, f_B—are not simultaneously tunable as fully independent variables. Whether a given trigger acts on a single parameter or on several at once depends on both the trigger mechanism and the initial state. The eight triggers fall into three regimes.

(i) Strictly selective control. Triggers 1 and 2, applied between NCAm and NC'Am, change $\chi_{c,A}$ (between 22.2% and 7.9%) while f_A stays at 0. The B-block is amorphous in both states, so $\chi_{c,B}$ and f_B are not defined. These two triggers offer the cleanest single-parameter control in the MMC and demonstrate that crystallinity and orientation are not intrinsically coupled within the A-block.

(ii) Predominant control with minor secondary changes. Trigger 7 (NCHC to NCNC) primarily relaxes f_B (-0.42 to 0) without affecting f_A. $\chi_{c,A}$ and $\chi_{c,B}$ vary by ~3%, which we attribute to small adjustments in crystallite population accompanying orientation relaxation. Within this tolerance, f_B can be addressed nearly in isolation.

(iii) Coupled responses. Triggers 3, 6, and 8 produce simultaneous changes in multiple structural parameters and cannot be used to address a single one. For example, trigger 8 (NCHC to HCHC) raises f_A from 0 to -0.45 while also increasing $\chi_{c,B}$ from 79.3% to 89.8%. The coupling reflects two physical mechanisms: strain-induced crystallization within each block, and templating between blocks where an oriented block promotes oriented crystallization in the other. Although these triggers sacrifice selectivity, they typically produce large concurrent gains in multiple properties (e.g., HCHC reaches σ = 0.39 GPa and U = 405 MJ/m^3), so the coupling is functionally useful.

Microstates between NC'Am and MCAm

In this section, WAXS characterization was employed to demonstrate the continuous transition from NC'Am to MCAm. By applying trigger 3 (cold strain under water) to NC'Am and carefully adjusting the strain level, the crystallinity and crystalline orientation of PCL could be finely tuned, thereby enabling the continuous transformation from NC'Am to MCAm.

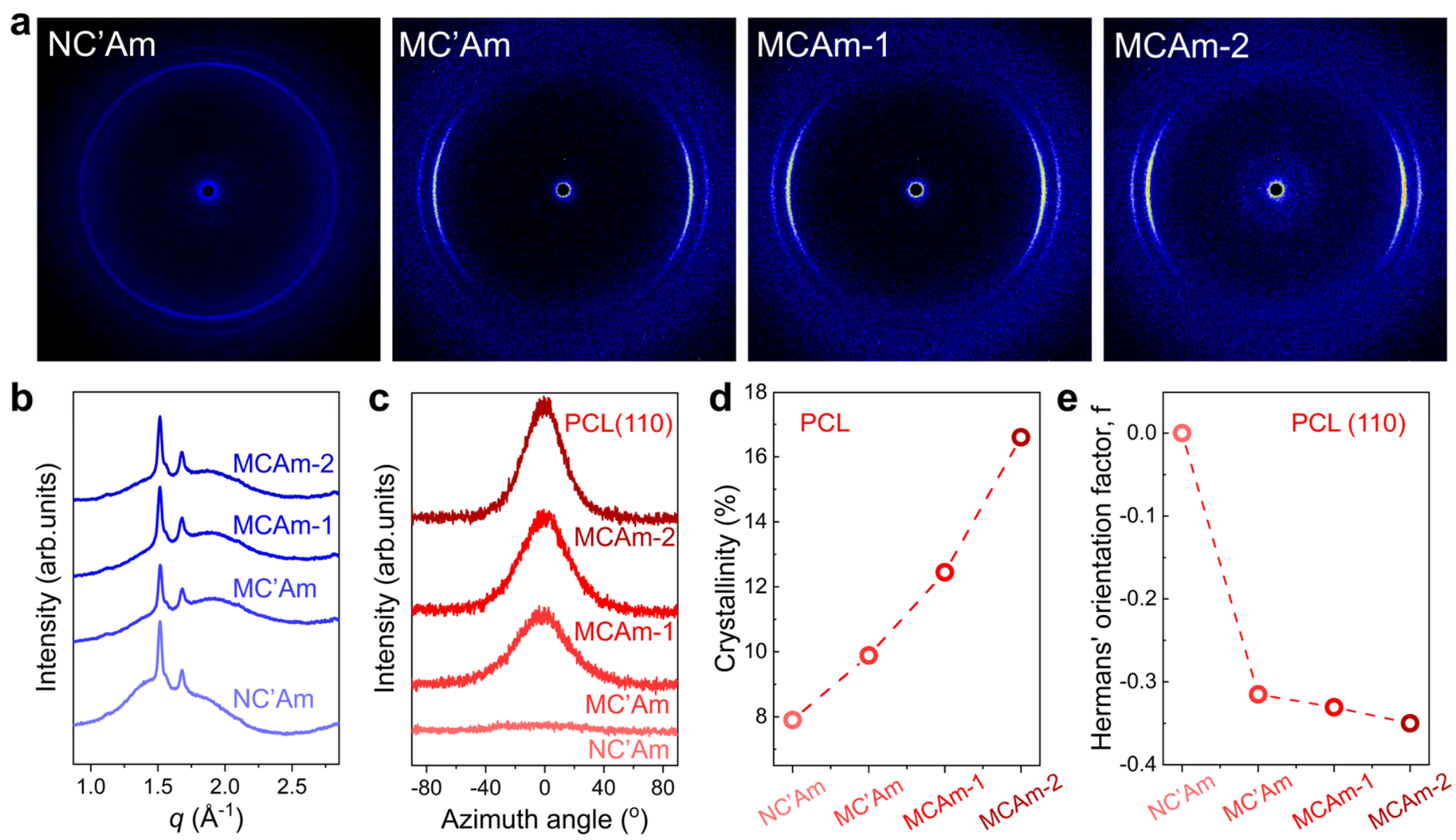

Fig. S2. WAXS characterization of microstates between NC'Am and MCAm. **(a)** Two-dimensional WAXS plots of microstates between NC'Am and MCAm. **(b)** One-dimensional WAXS scattering of microstates between NC'Am and MCAm. **(c)** One-dimensional scattering profiles of PCL (110) crystal plane from azimuthal angle integration of the 2D WAXS data. **(d)** Crystallinity of PCL calculated from the one-dimensional WAXS scattering plots. PCL exhibit increasing crystallinity with progressive strain-induced crystallization. **(e)** Hermans' orientation factor, f_A, determined from PCL (110) crystal plane.

Microstates between NCHC and HCHC

In this section, WAXS characterization was employed to demonstrate the continuous transition from NCHC to HCHC. By applying trigger 6 (dehydration under strain) to NC'Am and carefully adjusting the strain level, the crystallinity and crystalline orientation of both PCL and PEO could be finely tuned, thereby enabling the continuous transformation from NCHC to HCHC.

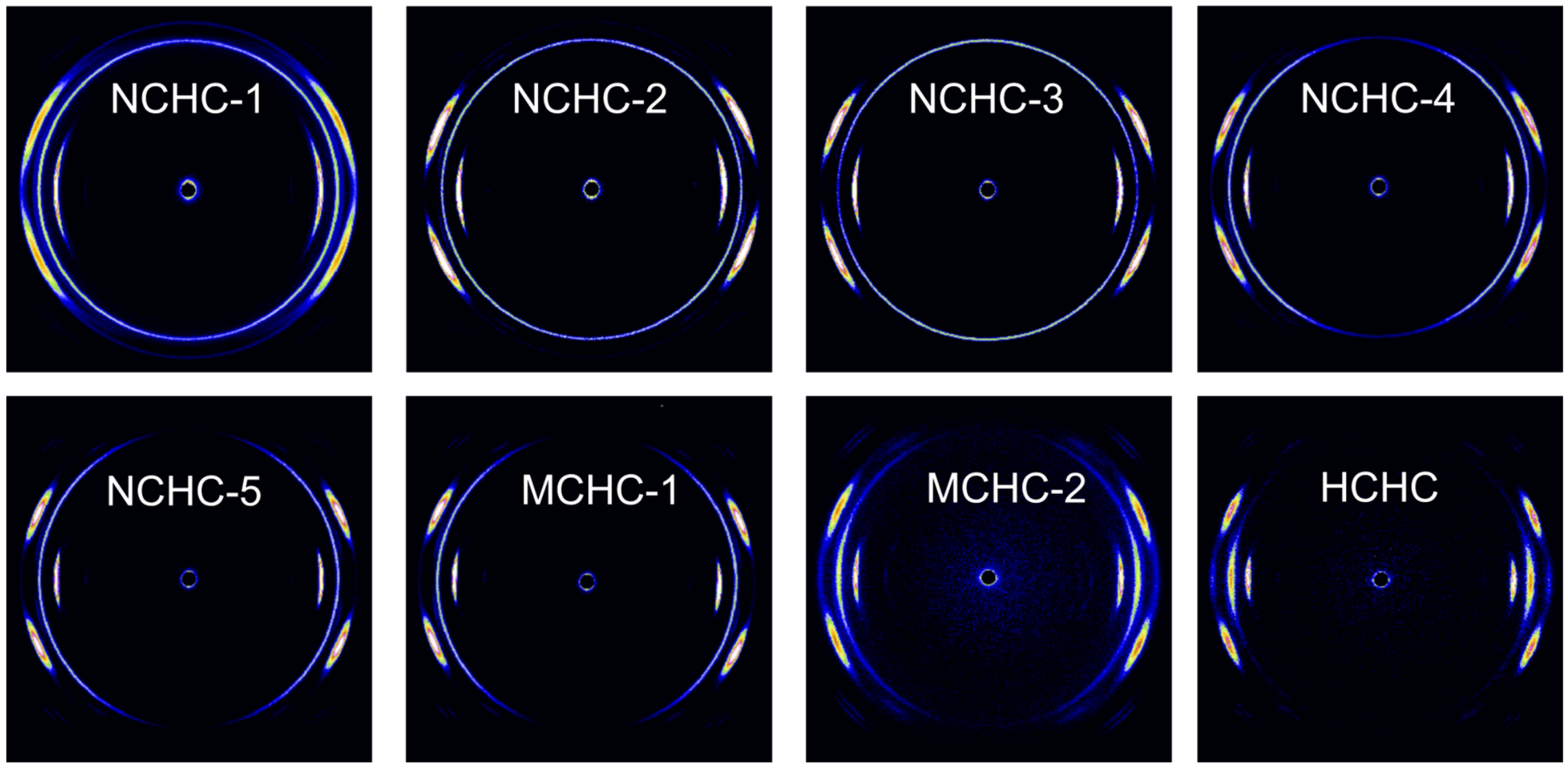

Fig. S3. Two-dimensional WAXS data of microstates between NCHC and HCHC.

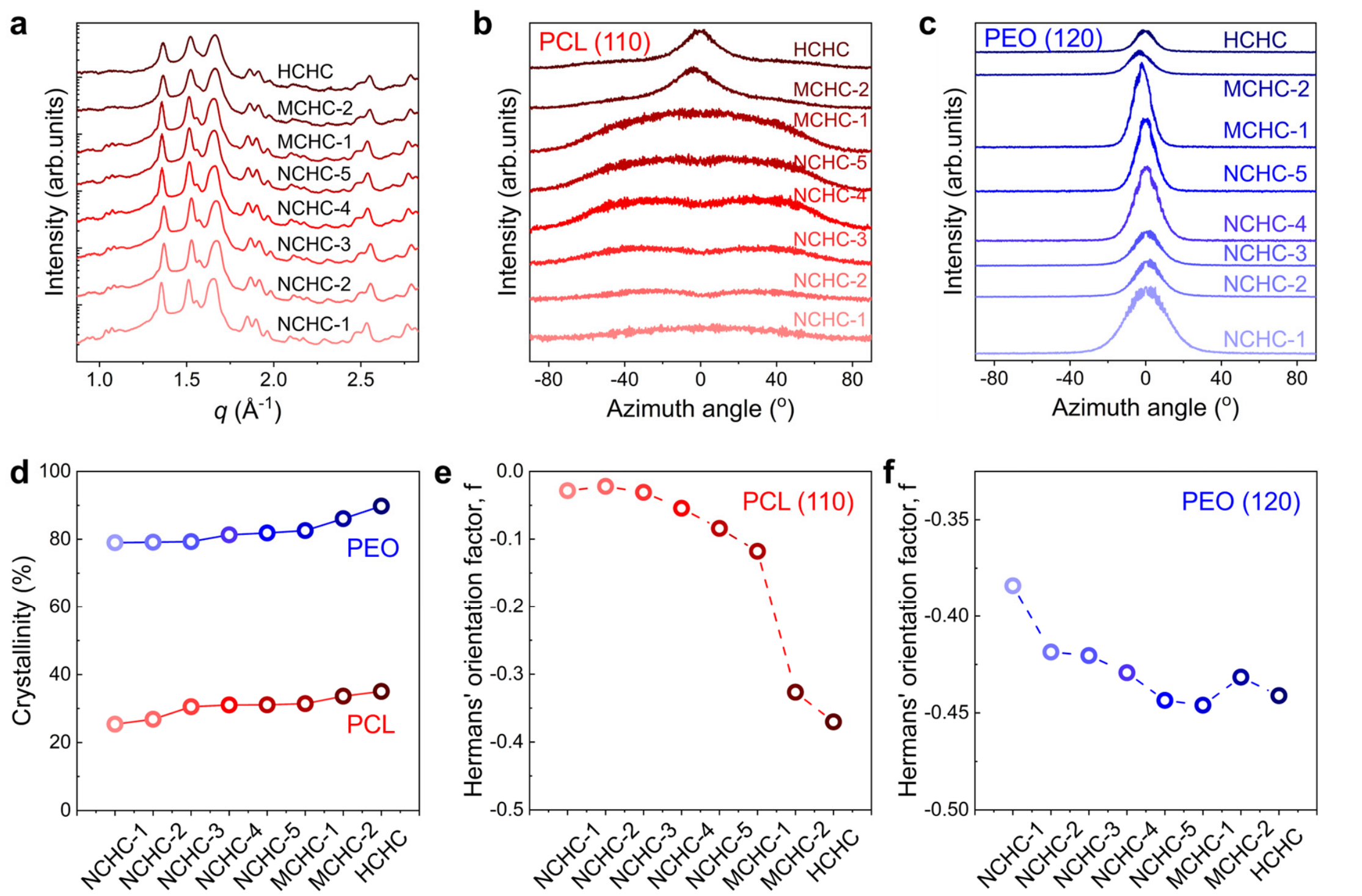

Fig. S4. (a) One-dimensional WAXS scattering of microstates between NCHC and HCHC. **(b, c)** One-dimensional scattering profiles of PCL (110) and PEO (120) crystal plane from azimuthal angle integration of the 2D WAXS data. **(d)** Crystallinity of PCL and PEO of microstates between NCHC and HCHC calculated from the one-dimensional WAXS scattering plot. **(e, f)** Hermans' orientation factor, f_A and f_B,

determined from PCL (110) and PEO (120) crystal planes.

Microstates between MCMC and HCHC

In this section, WAXS characterization was employed to demonstrate the continuous transition from MCMC to HCHC. By applying trigger 6 (Dehydration under strain) to MCAm and carefully adjusting the strain level, the crystallinity and crystalline orientation of both PCL and PEO could be simultaneously tuned, thereby enabling the continuous transformation from MCMC to HCHC.

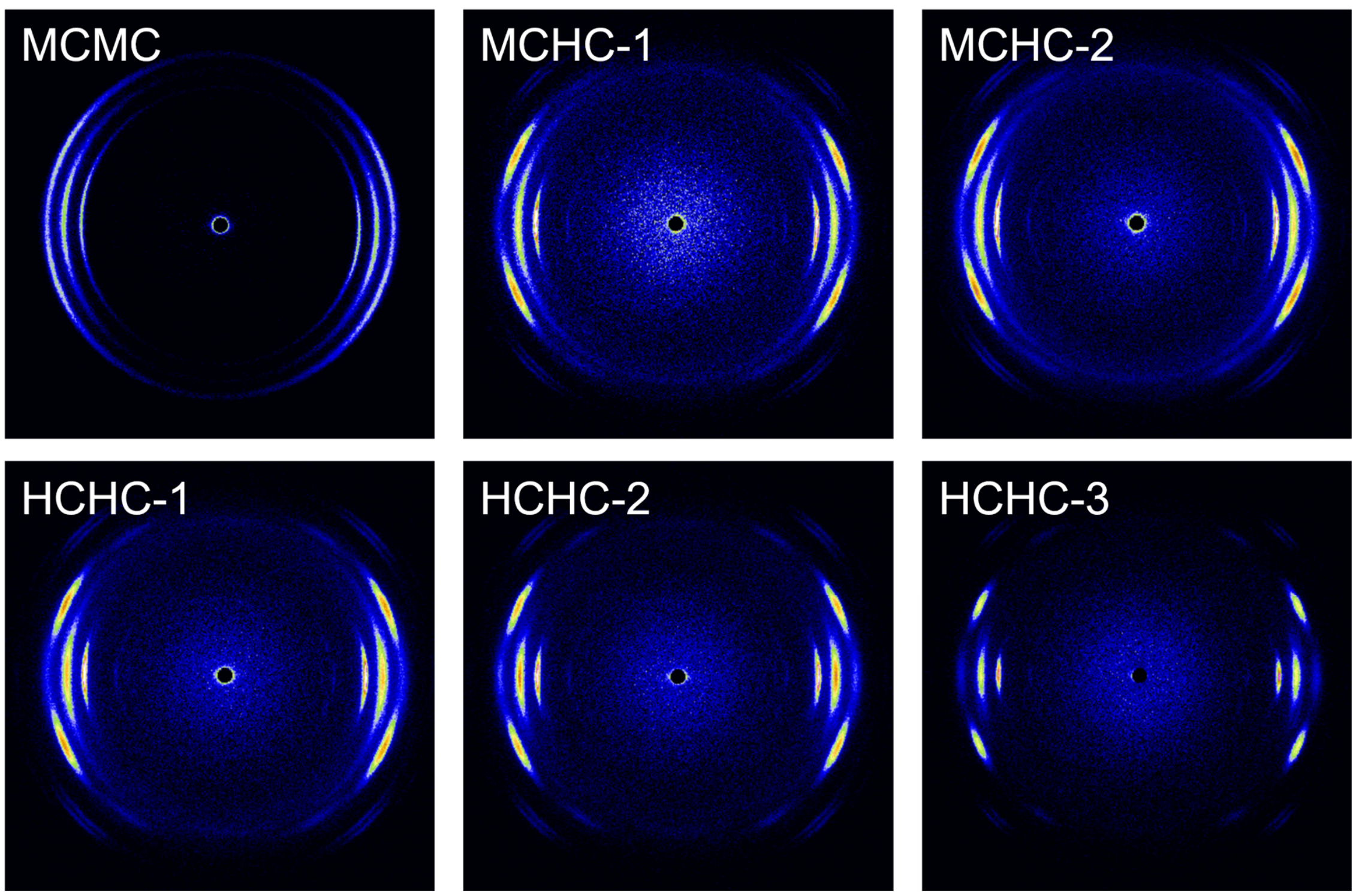

Fig. S5. Two-dimensional WAXS data of microstates between MCMC and HCHC.

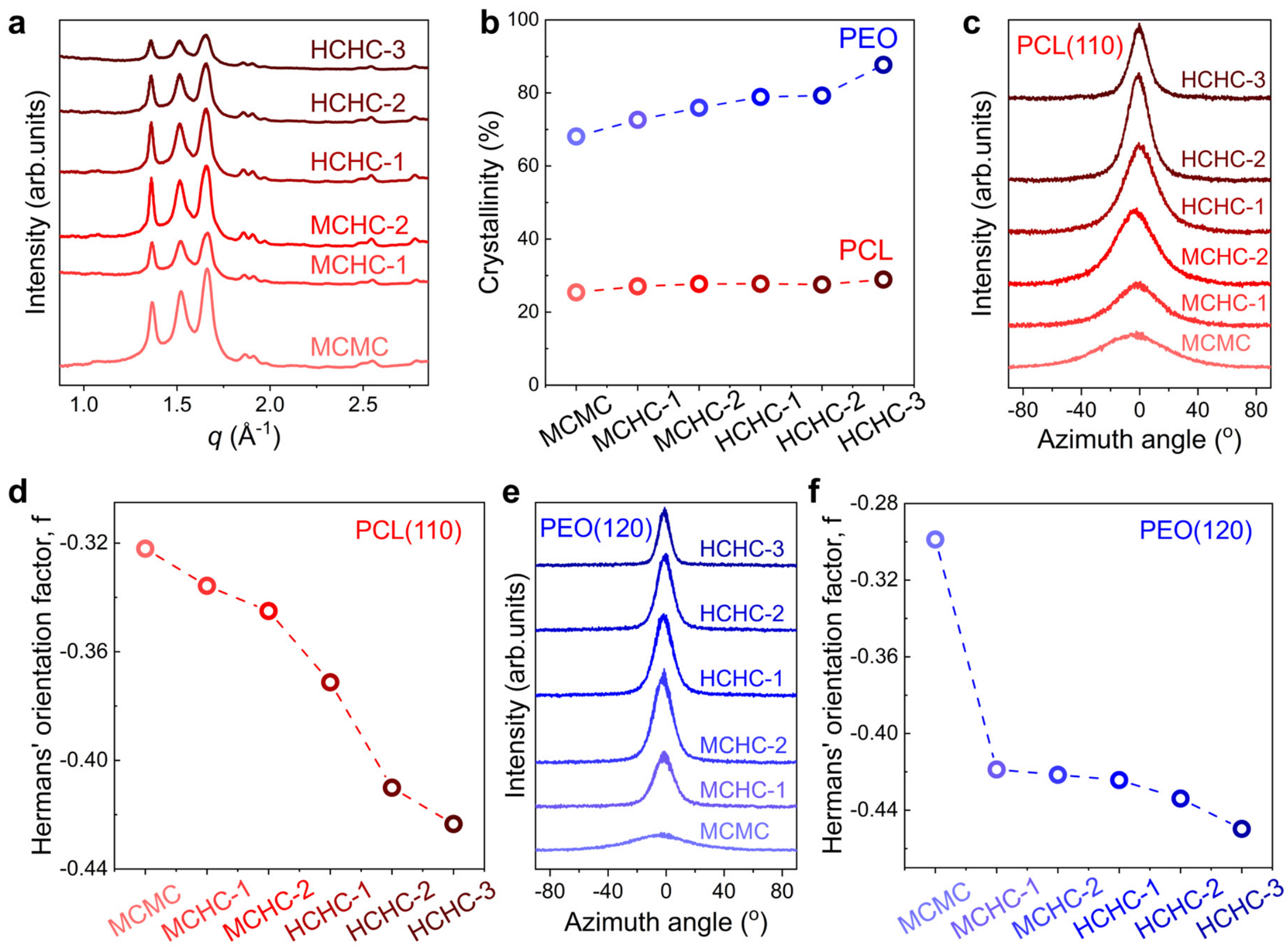

Fig. S6. (a) One-dimensional WAXS scattering of microstates between MCMC and HCHC. **(b)** Crystallinity of PCL and PEO of microstates between MCMC and HCHC calculated from the one-dimensional WAXS scattering plot. **(c)** One-dimensional scattering profiles of PCL (110) crystal plane from azimuthal angle integration of the 2D WAXS data. **(d)** Hermans' orientation factor f_A determined from PCL (110). **(e)** One-dimensional scattering profiles of PEO (120) crystal plane from azimuthal angle integration of the 2D WAXS data. **(f)** Hermans' orientation factor f_B determined from PEO (120) crystal planes.

3. Key states and microstates of MMC

Structure analysis of the eight key states

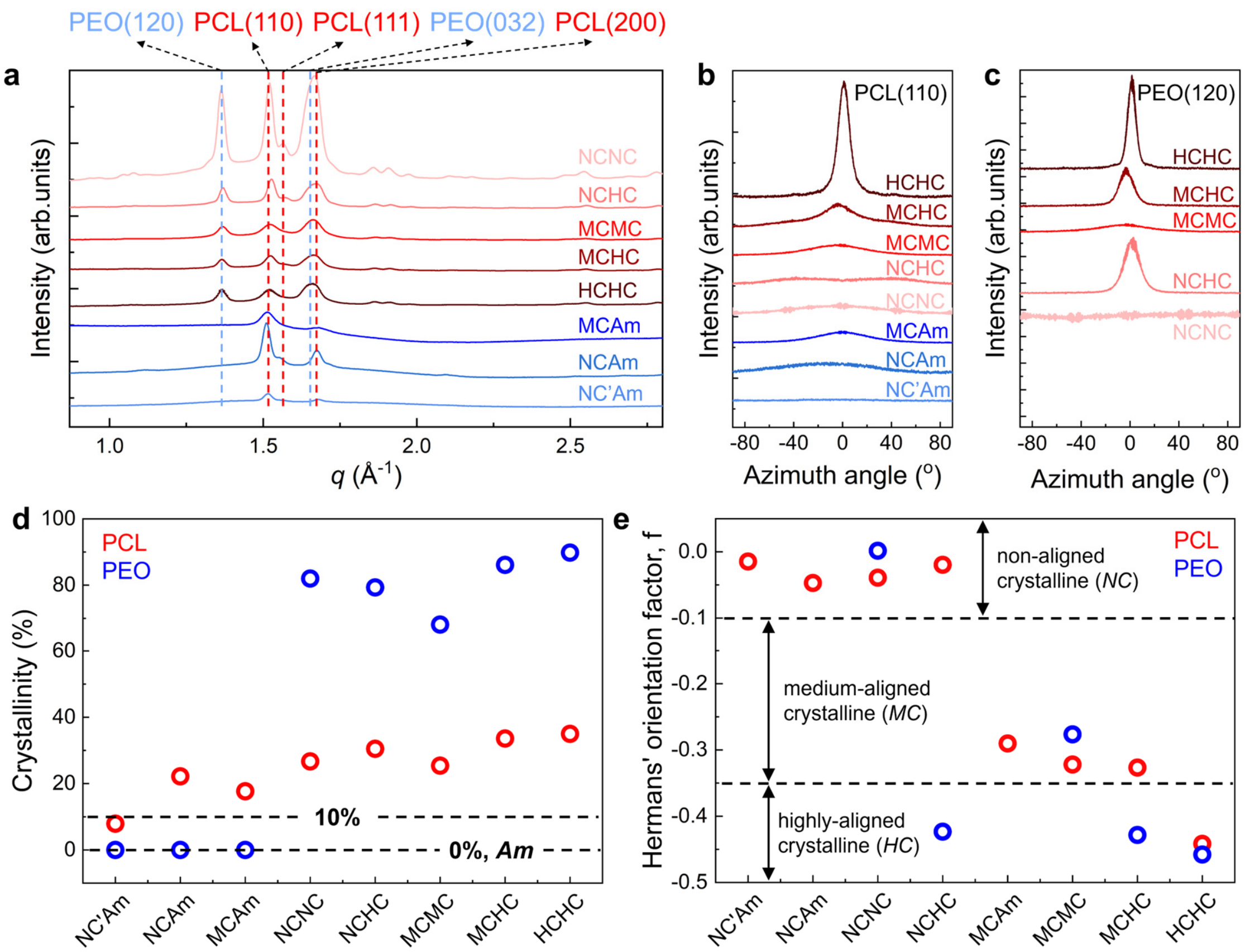

Fig. S7. WAXS analysis of the eight key states. (a) One-dimensional WAXS scattering peaks. **(b, c)** One-dimensional scattering profiles of PCL (110) crystal plane and PEO (120) crystal plane from azimuthal angle integration of the 2D WAXS data. **(d)** Crystallinity of PCL and PEO for eight key states. **(e)** Hermans' orientation factor, f_A and f_B, of the eight key states determined from PCL (110) and PEO (120) crystal planes.

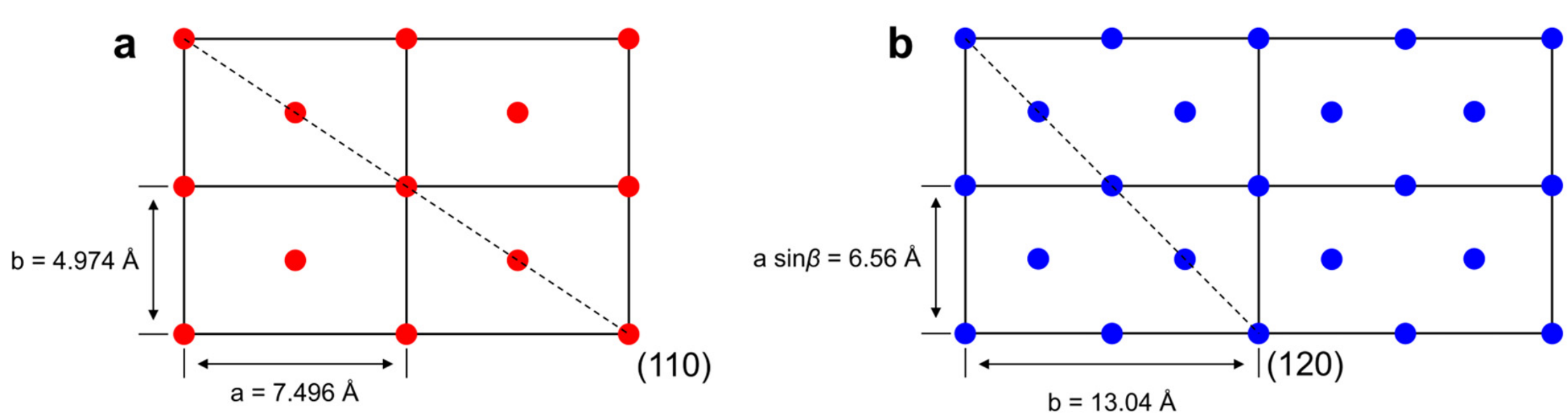

Fig. S8. Crystal structure of PCL and PEO. **(a)** Orthorhombic crystal system of PCL with the (110) lattice plane. **(b)** Monoclinic crystal system of PEO with the (120) lattice plane.

Table S3. Crystal system and lattice parameters of PCL and PEO.

Polymer	Crystal Systems	***a*** **(Å)**	***b*** **(Å)**	***c*** **(Å)**	***α*** **(º)**	***β*** **(º)**	***γ*** **(º)**
PCL	Orthorhombic	7.50	4.97	17.30	90	90	90
PEO	Monoclinic	8.05	13.04	19.48	90	125.4	90

4. Structure and mechanical properties of hydrogel states

Structure analysis of NC'Am

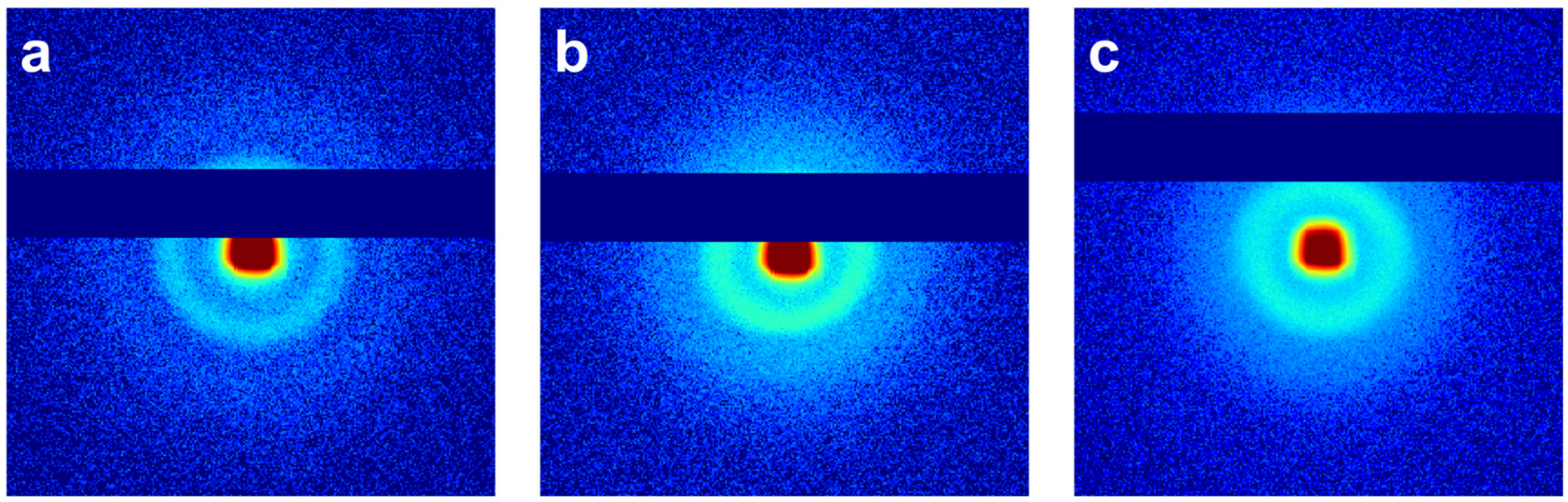

Fig. S9. 2D SAXS patterns of NC'Am with different hydrophobic block ratios. **(a)** $C_{215}O_{1818}C_{215}$, **(b)** $C_{323}O_{1818}C_{323}$, **(c)** $C_{400}O_{1818}C_{400}$. The precursor solution concentration is 10 wt%.

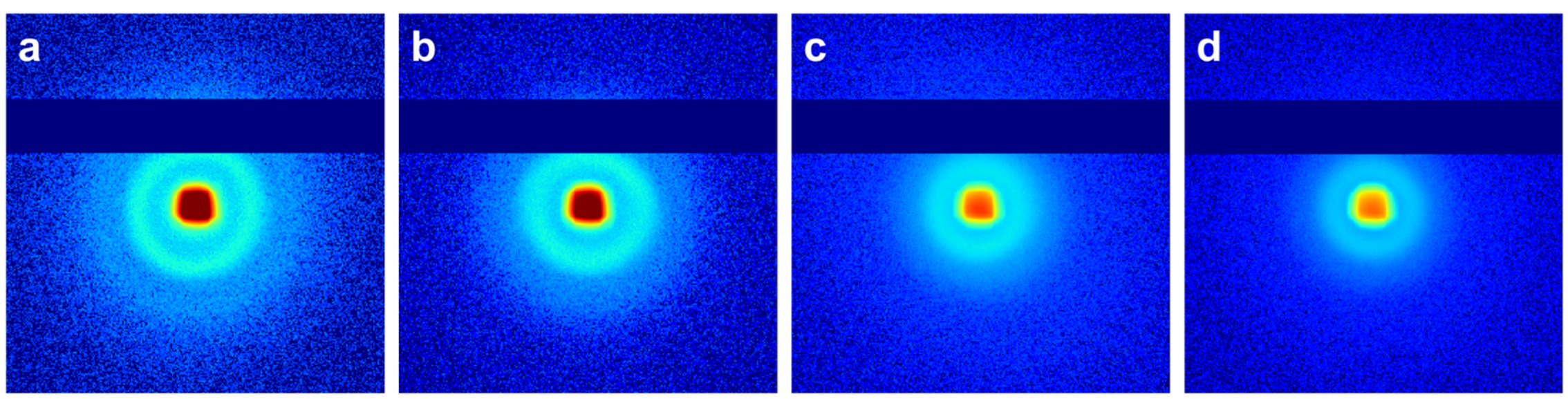

Fig. S10. 2D SAXS patterns of NC'Am with different precursor solution concentration. **(a)** 5 wt%, **(b)** 10 wt%, **(c)** 15 wt%, **(d)** 17 wt%. The polymer used was $C_{400}O_{1818}C_{400}$.

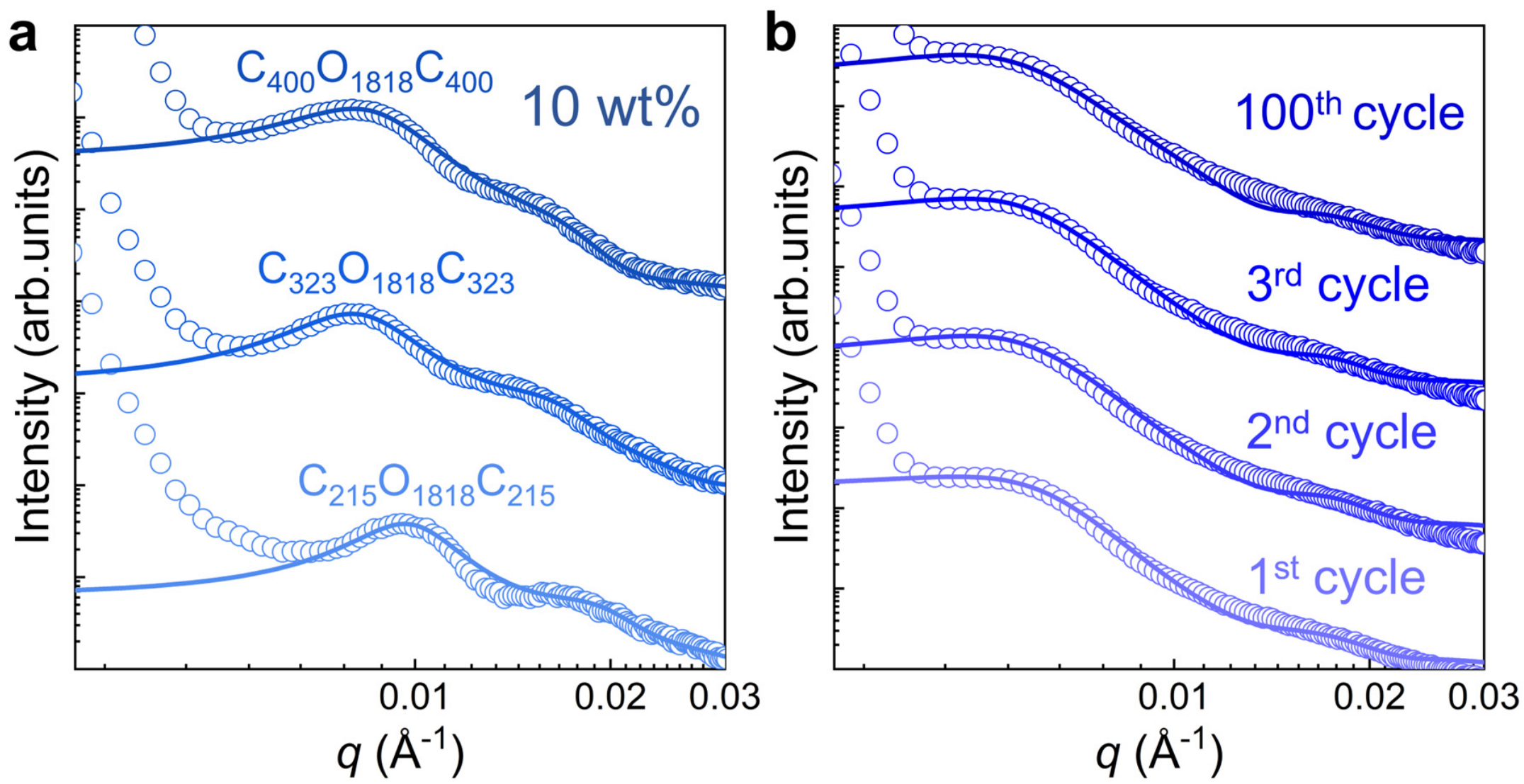

Fig. S11. (a) Experimental and modeled SAXS data of NC'Am with different hydrophobic block ratios. **(b)** Experimental and modeled SAXS data of the system reset to NC'Am after 100 cycles of modulus switching. The modeled data was based on a spherical form factor and a Percus–Yevick structure factor.

Table S4. Parameters for modelling SAXS scattering patterns of NC'Am state with different hydrophobic block ratios and precursor solution concentrations.

**Precursor solution concentration**	R_c**(nm)**	σ_c**(nm)**	D_{hs}**(nm)**	φ_{hs}
$C_{215}O_{1818}C_{215}$ (10 wt%)	26.75	16.15	64.16	34.07
$C_{323}O_{1818}C_{323}$ (10 wt%)	27.75	14.85	75.15	30.40
$C_{400}O_{1818}C_{400}$ (10 wt%)	29.75	13.41	71.15	27.57
$C_{400}O_{1818}C_{400}$ (17 wt%)	37.08	13.11	94.14	23.74
$C_{400}O_{1818}C_{400}$ (15 wt%)	33.75	11.86	90.14	22.91
$C_{400}O_{1818}C_{400}$ (10 wt%)	29.75	13.41	71.15	27.57
$C_{400}O_{1818}C_{400}$ (5 wt%)	28.75	15.80	68.15	31.07

R_c is the mean value of the hydrophobic micelle core radius and σ_c is the standard deviation. D_{hs} is the mean value of the center-to-center distance, and φ_{hs} is the hard sphere volume faction.

Mechanical properties of hydrogel states

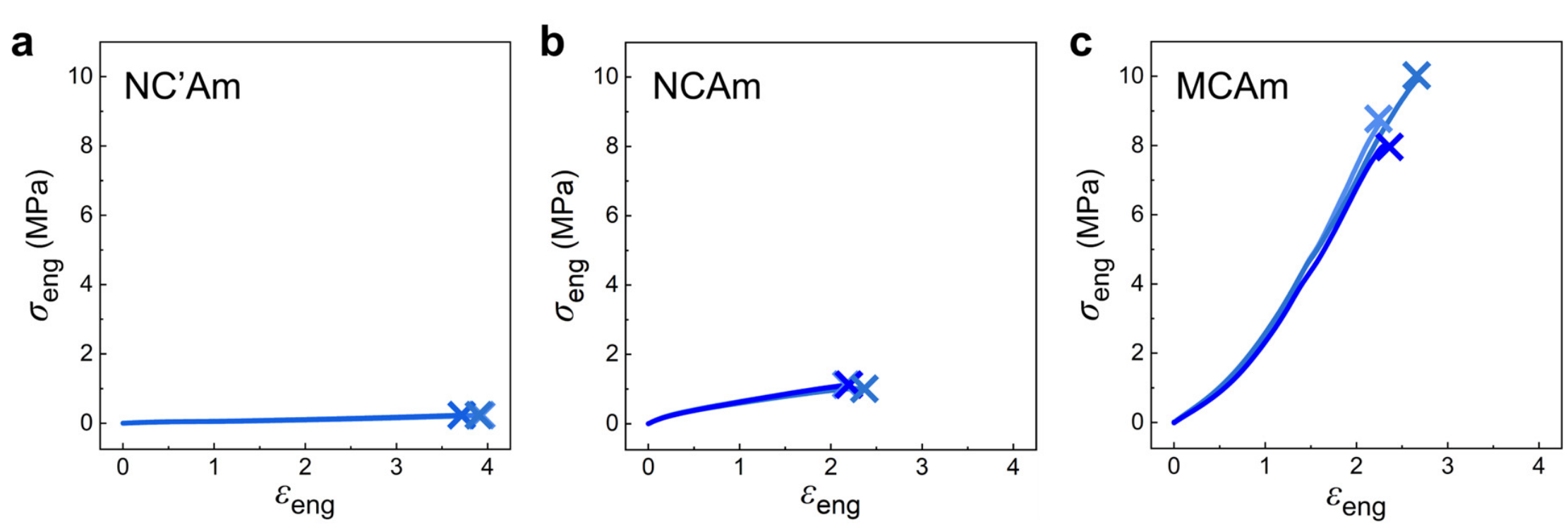

Fig. S12. Tensile stress–strain curves of different hydrogel states ($C_{400}O_{1818}C_{400}$). **(a)** NC'Am, **(b)** NCAm, and **(c)** MCAm. The strain rate was 1 min^{-1}.

Table S5. Mechanical properties of hydrogel state NC'Am, NCAm, and MCAm ($C_{400}O_{1818}C_{400}$).

Hydrogel state	Elongation at break	Tensile Strength (MPa)	Young's modulus (MPa)	Toughness (MJ m^{-3})
NC'Am	3.78±0.12	0.2±0.0	0.1±0.0	0.4±0.0
NCAm	2.2±0.1	1.1±0.1	1.3±0.0	1.4±0.1
MCAm	2.4±0.2	8.8±1.0	1.8±0.1	9.2±2.2

Comparison among NC'Am, NCAm, and MCAm reveals different contributions of $\chi_{c,A}$ and f_A: From NC'Am to NCAm ($f_A = 0$, $\chi_{c,A}$ increasing from 7.9% to 22.2%), all three metrics rise markedly: E increases from 0.1 to 1.3 MPa (~13-fold), σ from 0.2 to 1.1 MPa (~5.5-fold), and U from 0.4 to 1.4 MJ m^{-3} (~3.5-fold), indicating that $\chi_{c,A}$ establishes the fundamental mechanical framework of the hydrogel, with the most pronounced relative impact on Young's modulus. At comparable $\chi_{c,A}$ (NCAm vs MCAm), varying f_A (0 to -0.35) selectively amplifies strength and toughness, where σ rises from 1.1 to 8.8 MPa (~8-fold) and U from 1.4 to 9.2 MJ m^{-3} (~6.6-fold), while E increases only modestly from 1.3 to 1.8 MPa (~1.4-fold).

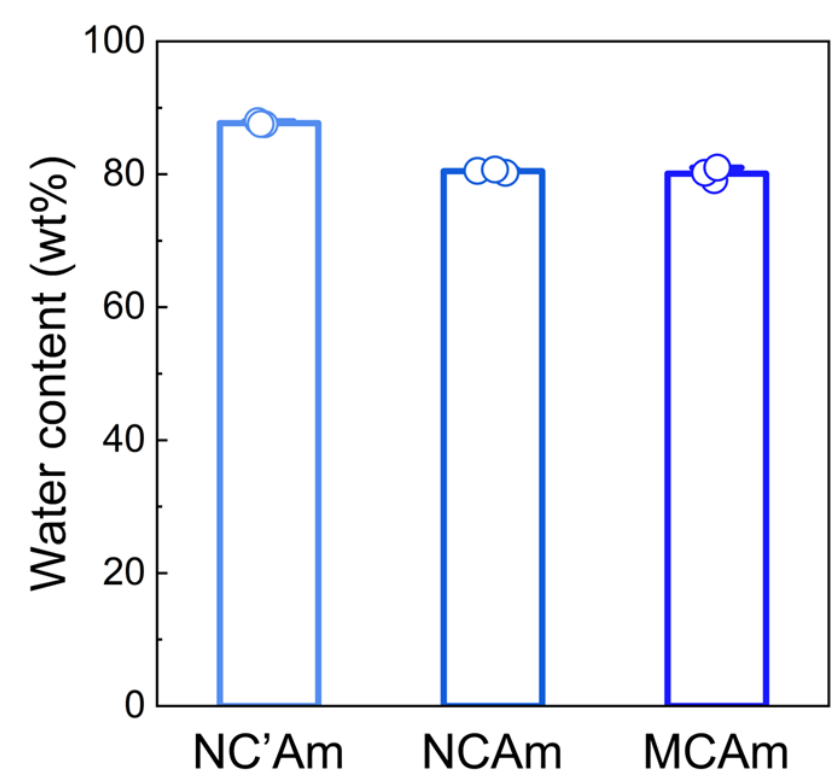

Fig. S13. Water content of the hydrogel states NC'Am, NCAm, and MCAm ($C_{400}O_{1818}C_{400}$). Bars show mean values, error bars represent s.d., circles indicate individual measurements (n = 3 independent measurements).

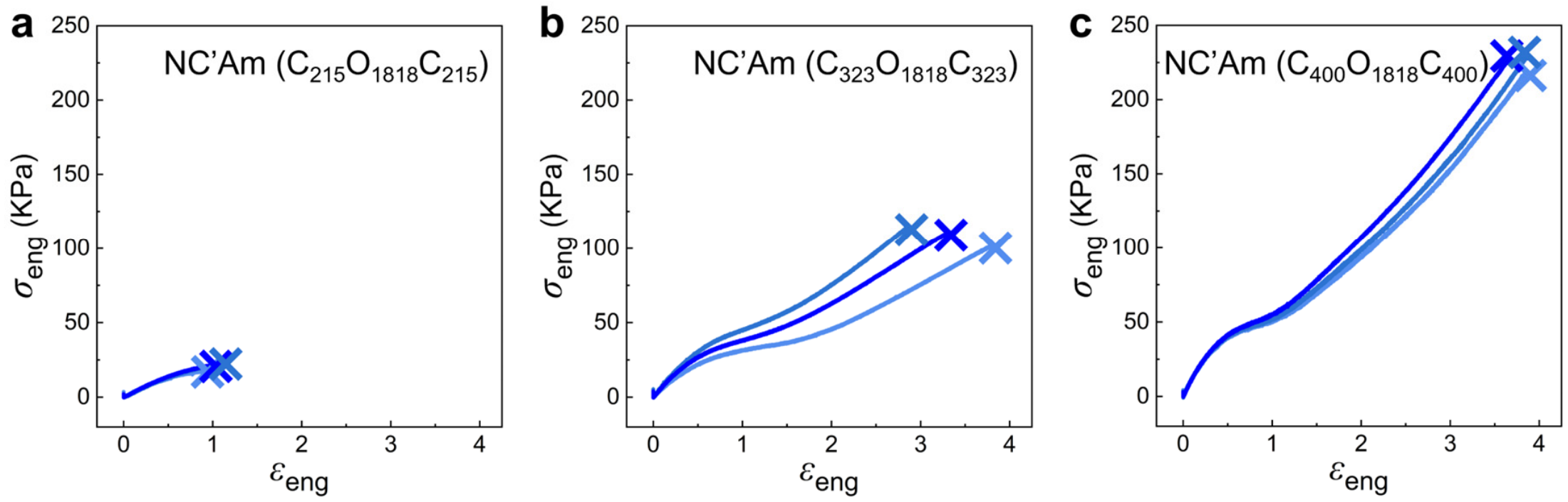

Fig. S14. Tensile stress–strain curves of NC'Am with different hydrophobic block ratios. **(a)** $C_{215}O_{1818}C_{215}$, **(b)** $C_{323}O_{1818}C_{323}$, **(c)** $C_{400}O_{1818}C_{400}$. The strain rate was 1 min^{-1}.

Table S6. Mechanical performances of NC'Am state with different hydrophobic block ratios.

**Samples**	**Elongation at break**	**Tensile strength (KPa)**	**Young's modulus (KPa)**	**Toughness (KJ m^{-3})**
$C_{215}O_{1818}C_{215}$	1.0±0.1	20.0±2.6	30.5±1.5	11.6±2.4
$C_{323}O_{1818}C_{323}$	3.3±0.5	108.0±6.2	69.5±10.2	177.2±11.3
$C_{400}O_{1818}C_{400}$	3.8±0.1	224.5±7.1	109.3±2.4	389.5±13.1

5. Switch between hydrogel and dry states

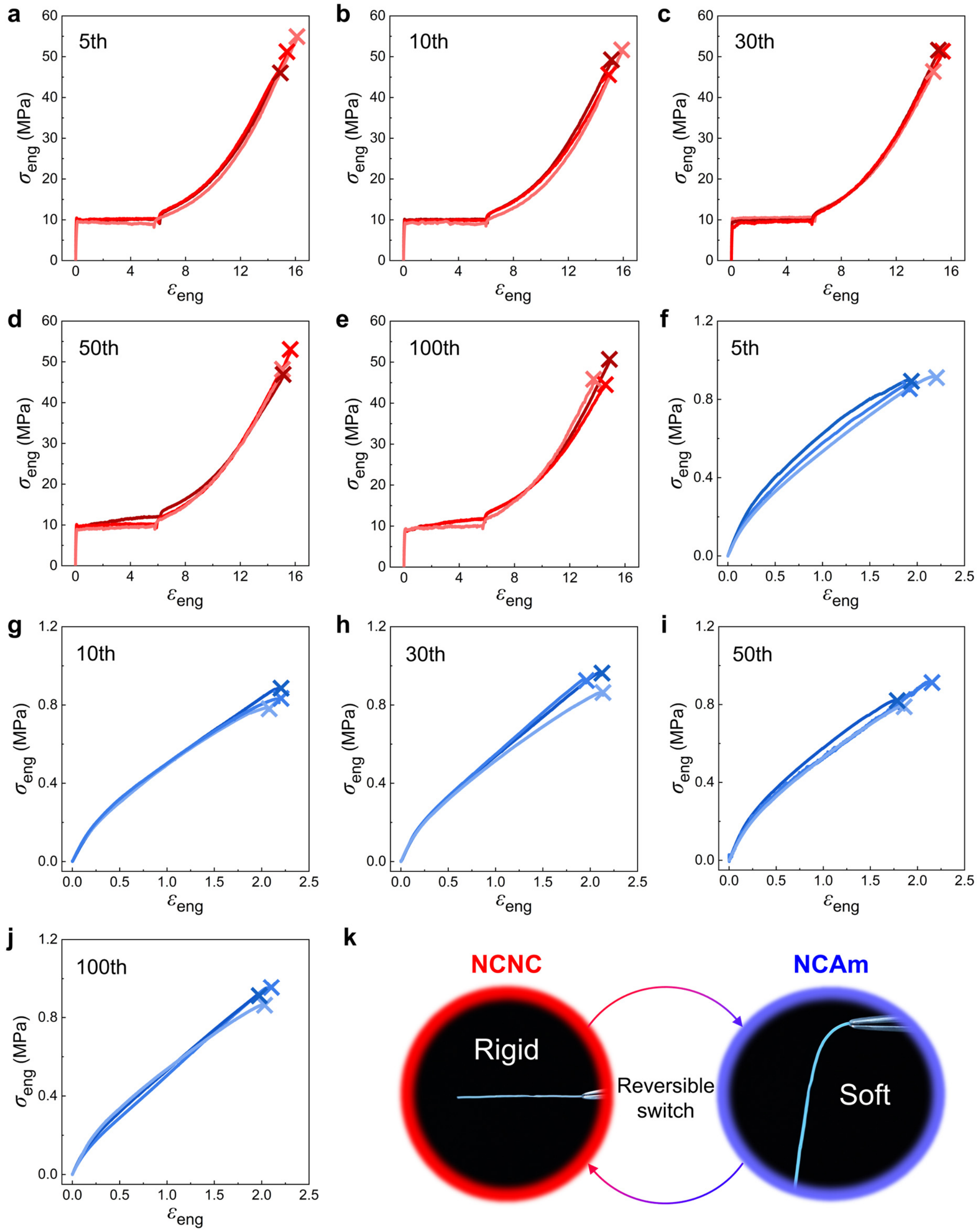

Fig. S15. Mechanical performance of dry (NCNC) and hydrogel (NCAm) state over 100 hydration–dehydration cycles ($C_{400}O_{1818}C_{400}$). **(a–e)** Tensile stress–strain curves of NCNC after 5th, 10th, 30th, 50th and 100th hydration–dehydration cycles. **(f–j)** Tensile stress–strain curves of NCAm after undergoing 5th,

10th, 30th, 50th and 100th hydration–dehydration cycles. **(k)** Pictures demonstrate reversible modulus switch.

Table S7. Toughness, Young's modulus, strain at break and strength of NCNC state over 100 hydration–dehydration cycles.

**NCNC**	**Toughness ($MJ\ m^{-3}$)**	**Young's modulus (GPa)**	**Elongation at break**	**Tensile strength (MPa)**
5th	302.3±24.0	0.3±0.1	15.5±0.6	50.0±4.3
10th	287.9±13.0	0.3±0.1	15.4±0.5	48.0±3.1
30th	293.0±16.7	0.3±0.08	15.0±0.4	49.1±2.8
50th	302.3±17.6	0.3±0.05	15.3±0.3	48.5±3.2
100th	278.3±25.3	0.3±0.04	14.4±0.5	46.3±3.2

Table S8. Toughness, Young's modulus, strain at break and strength of NCAm state over 100 dehydration–hydration cycles.

**NCAm**	**Toughness ($MJ\ m^{-3}$)**	**Young's modulus (MPa)**	**Elongation at break**	**Tensile strength (MPa)**
5th	1.11±0.10	1.15±0.13	2.0±0.2	0.90±0.02
10th	1.08±0.07	0.97±0.07	2.1±0.1	0.83±0.05
30th	1.10±0.06	1.06±0.04	2.1±0.1	0.92±0.05
50th	0.99±0.14	1.06±0.10	1.9±0.2	0.84±0.06
100th	1.04±0.03	1.06±0.06	2.0±0.1	0.91±0.03

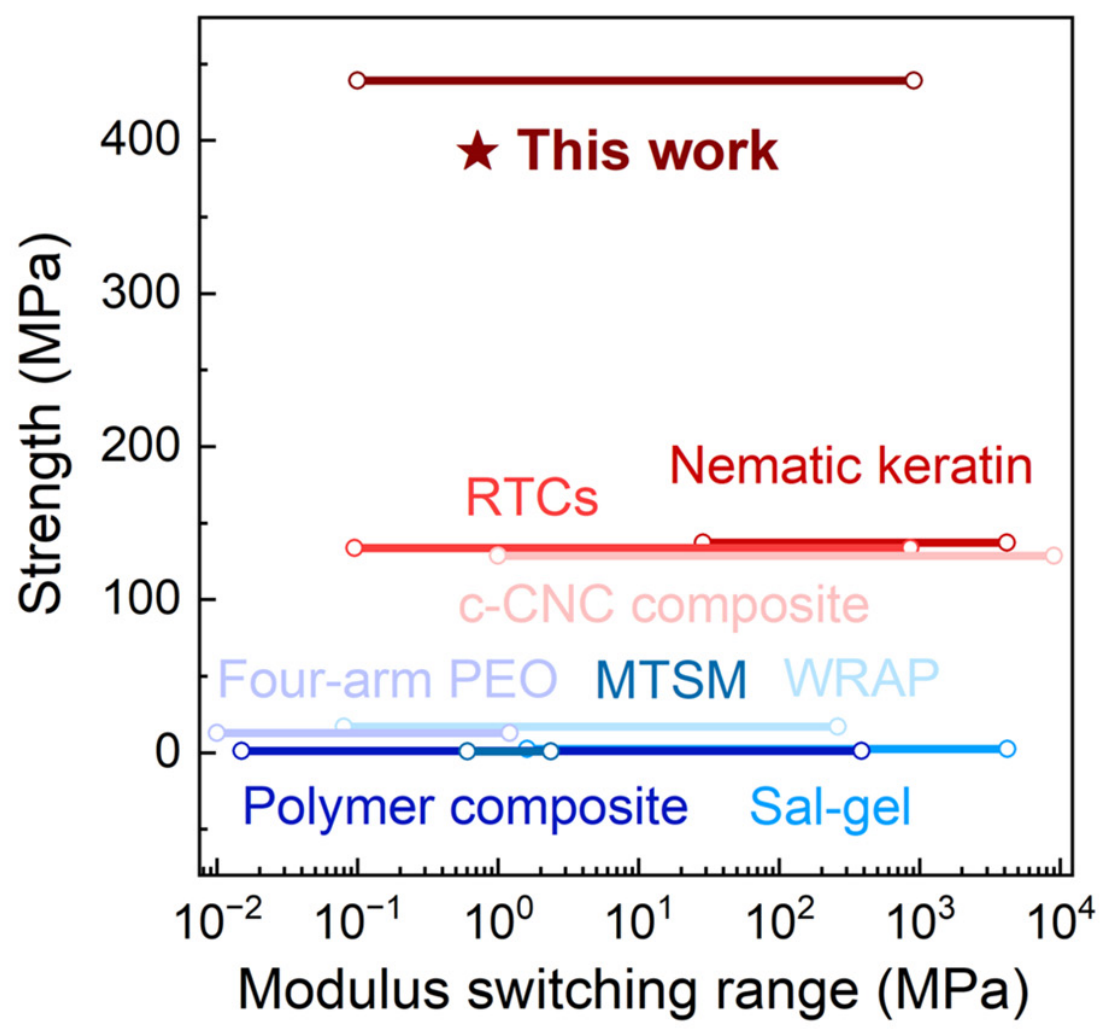

Fig. S16. Modulus switching range of this work compared to previously reported system with respect to material strength and toughness.

Table S9. Property comparison of different modulus-switching materials from literatures.

Material	Modulus (MPa)		Modulus switch ratio	Elongation at break	Strength (MPa)	Toughness (MJ m^{-3})
	Soft	Rigid				
**This work**	0.1	880	8800	1.79	388	405
WRAP(*2*)	0.1	260	3250	4	17	55.8
Polymer Nanocomposites(*3*)	1.6	4200	2625	6	2.3	8.7
Nematic keratin-Anisotropic(*4*)	28.7	4180	145.6	1	137.2	89.7
Nematic keratin-Isotropic(*4*)	0.3	30.9	100.3	0.01	3.3	0.02
Four-arm PEO hydrogel(*5*)	0.01	1.21	121	6.2	13	64.9
Sal-gel(*6*)	0.02	385	25667	0.1	1	0.04
Nanocomposites (c-CNCs)(*7*)	1	9000	9000	0.01	128.6	1.12
Nanocomposites (t-CNCs)(*7*)	160	13700	85.6	0.01	140.4	0.95
APS elastomers(*8*)	4.6	540	118	0.06	16.4	0.65
PCM microcomposites(*9*)	1.8	6.1	3.4	2.9	0.4	0.6
Rigidity tunable composites (RTCs)(*10*)	0.1	860	9052	0.14	134	9.4
MTSM(*11*)	0.6	2.4	3.9	9.8	0.9	7.0

To determine the volumetric change of the sample during switching between hydrogel state and dry state, the same fiber was brought into different states using the trigger protocol shown in Fig. 2 of the manuscript. The cross-sectional area A of the fiber in each state was measured by optical microscopy, and the fiber length L was recorded; from these measurements the volume V for each state was calculated by followed equation:

$$V_{\text{material state}} = A \times L$$

The volumetric strain associated with the transition between states was calculated using the following equation:

$$\varepsilon_V = \frac{V_{\text{hydrogel state}} - V_{\text{dry state}}}{V_{\text{dry state}}} \times 100\%$$

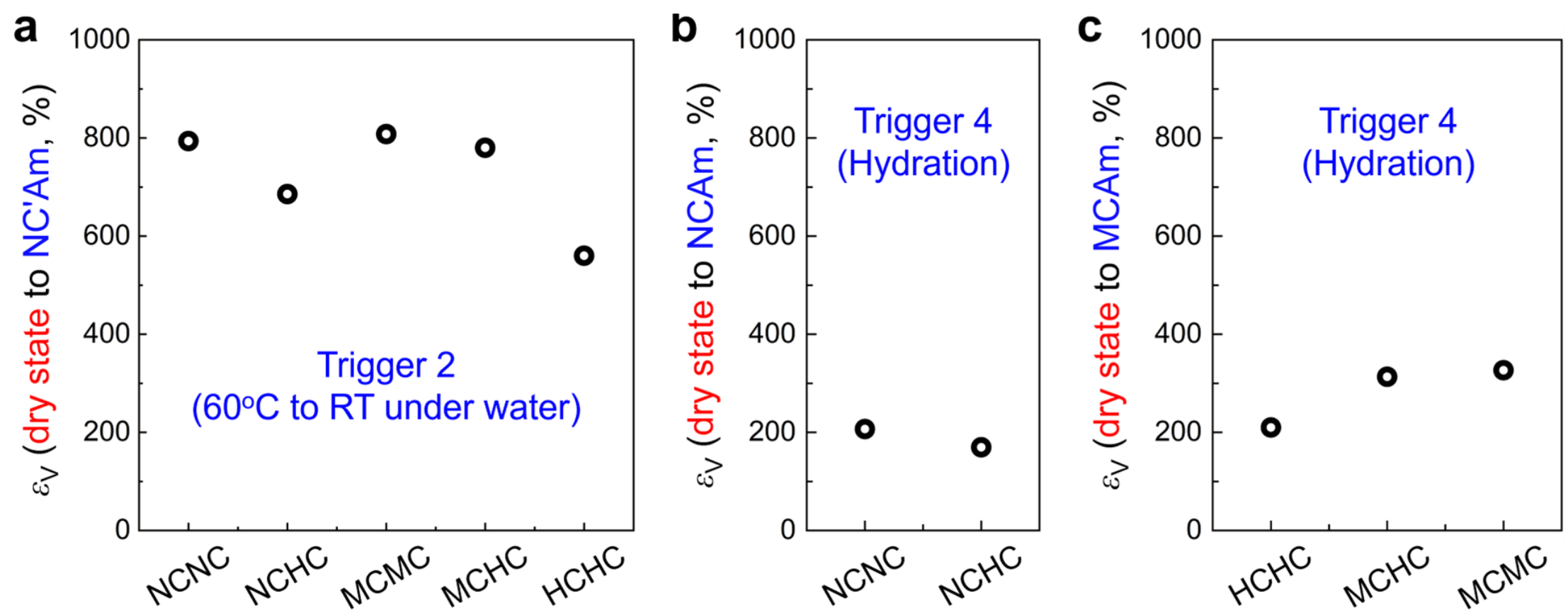

Fig. S17. Volumetric change when dry state transforms into hydrogel state. (a) NCNC, NCHC, MCMC, MCHC and HCHC transform into NC'Am through 60°C to RT under water (Trigger 2). **(b)** NCNC and NCHC transform into NCAm through hydration (Trigger 4). **(c)** HCHC, MCHC and MCMC transform into MCAm through hydration (Trigger 4).

ε_V depends strongly on the trigger pathway. Fibers transformed via Trigger 2 (heating to 60 °C in water, followed by cooling to room temperature under water) reach the NC'Am state with ε_V in the range of ~560%–800%, whereas the same compositions transformed via Trigger 4 (direct hydration at room temperature) reach the NCAm or MCAm state with ε_V of ~170%–330%. This pathway-dependence reflects the state of the crystalline physical crosslinks during hydration. In Trigger 2, heating above the melting temperature of PCL crystalline domains transiently releases these crosslinks, allowing the network to swell more extensively in water before the crosslinks reform upon cooling. In Trigger 4, the crystalline crosslinks remain intact throughout hydration and constrain the extent of swelling. The hydrogel states reached by the two pathways (NC'Am vs NCAm/MCAm) therefore correspond to distinct network configurations.

6. Mechanical properties of dry states

Since water content significantly affects the mechanical properties, we determined the residual water content of samples in the dry state by TGA using the following equation:

$$\text{Water content} = \frac{Mass_{30^{\circ}C} - Mass_{150^{\circ}C}}{Mass_{30^{\circ}C}} \times 100\%$$

$Mass_{30^{\circ}C}$ indicate the mass fraction of dry state material at 30ºC. $Mass_{150^{\circ}C}$ indicate the mass fraction of dry state material when the temperature reaches 150ºC.

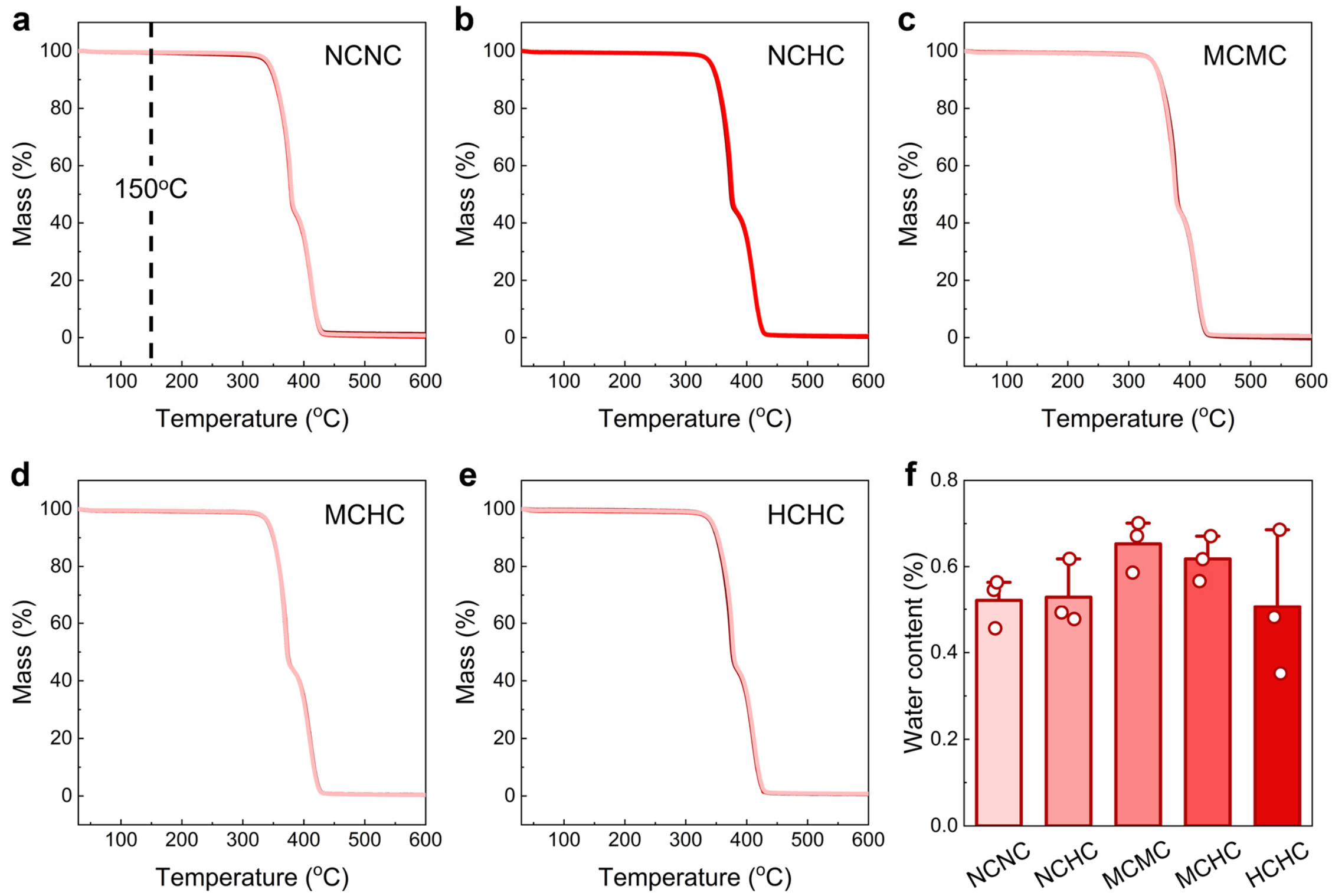

Fig. S18. Water contents of dry states material. Thermogravimetric analysis (TGA) curve of NCNC **(a)**, NCHC **(b)**, MCMC **(c)**, MCHC **(d)** and HCHC **(e)**. Mass loss was recorded under N_2 at a heating rate of 10 ºC·min⁻¹ from 30ºC to 600ºC. **(f)** Water content of NCNC, NCHC, MCMC, MCHC and HCHC, determined from TGA curves by accounting for mass loss due to water removal in the 30–150ºC temperature range. Bars show mean values, error bars represent s.d., circles indicate individual measurements (n = 3 independent measurements).

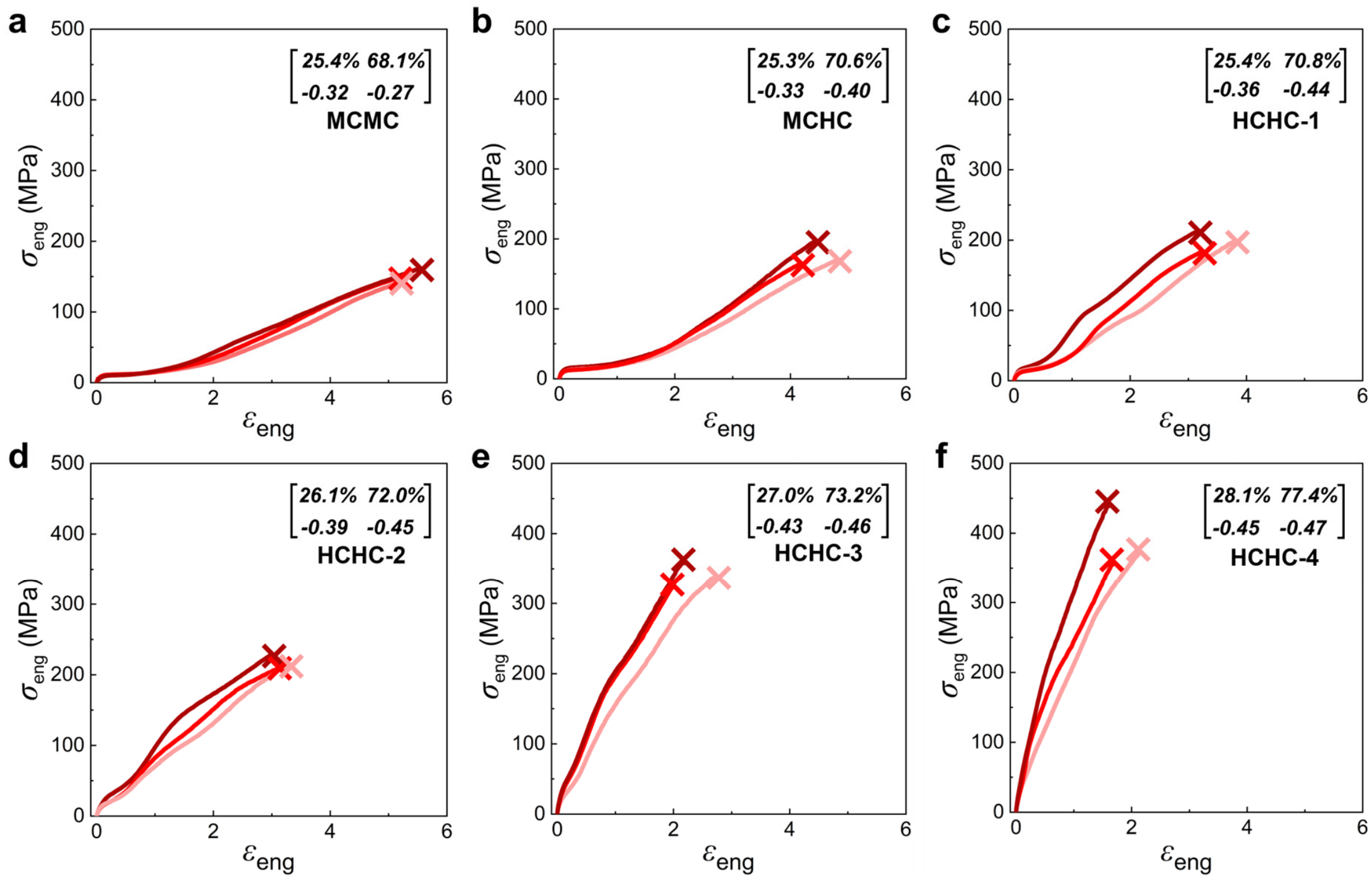

Fig. S19. Mechanical performance of microstates between MCMC and HCHC ($C_{400}O_{1818}C_{400}$). Tensile stress-strain curves of **(a)** MCMC, **(b)** MCHC, and **(c–f)** HCHC with different matrix parameters. Tensile stress-strain curves were obtained at a strain rate of 1 min^{-1}.

Dry-state mechanical properties are determined jointly by the four structural parameters $\chi_{c,A}$, $\chi_{c,B}$, f_A, f_B. In general. In the dry states examined here, the four parameters act in the same direction: higher crystallinity and orientation lead to enhanced mechanical performance (modulus, strength, and toughness). Within this overall trend, the parameters play complementary roles. Matrix crystallization ($\chi_{c,B}$) dominates the hydrogel → dry state transition; together with water removal, the increase of $\chi_{c,B}$ from 0 to 68–90% facilitates the modulus jump from MPa to sub-GPa scale (e.g., NCAm → NCNC: 1.3 MPa → 0.23 GPa). f_A and f_B amplify performance via orientation-induced reinforcement. Comparing NCNC and NCHC at comparable $\chi_{c,B}$, the introduction of a negative f_B (-0.42) approximately doubles E (0.23 → 0.30 GPa) and raises U by ~32%; comparing MCHC and HCHC, a more negative f_A further roughly doubles both E and σ, yielding HCHC's 0.88 GPa modulus and 405 MJ m^{-3} toughness.

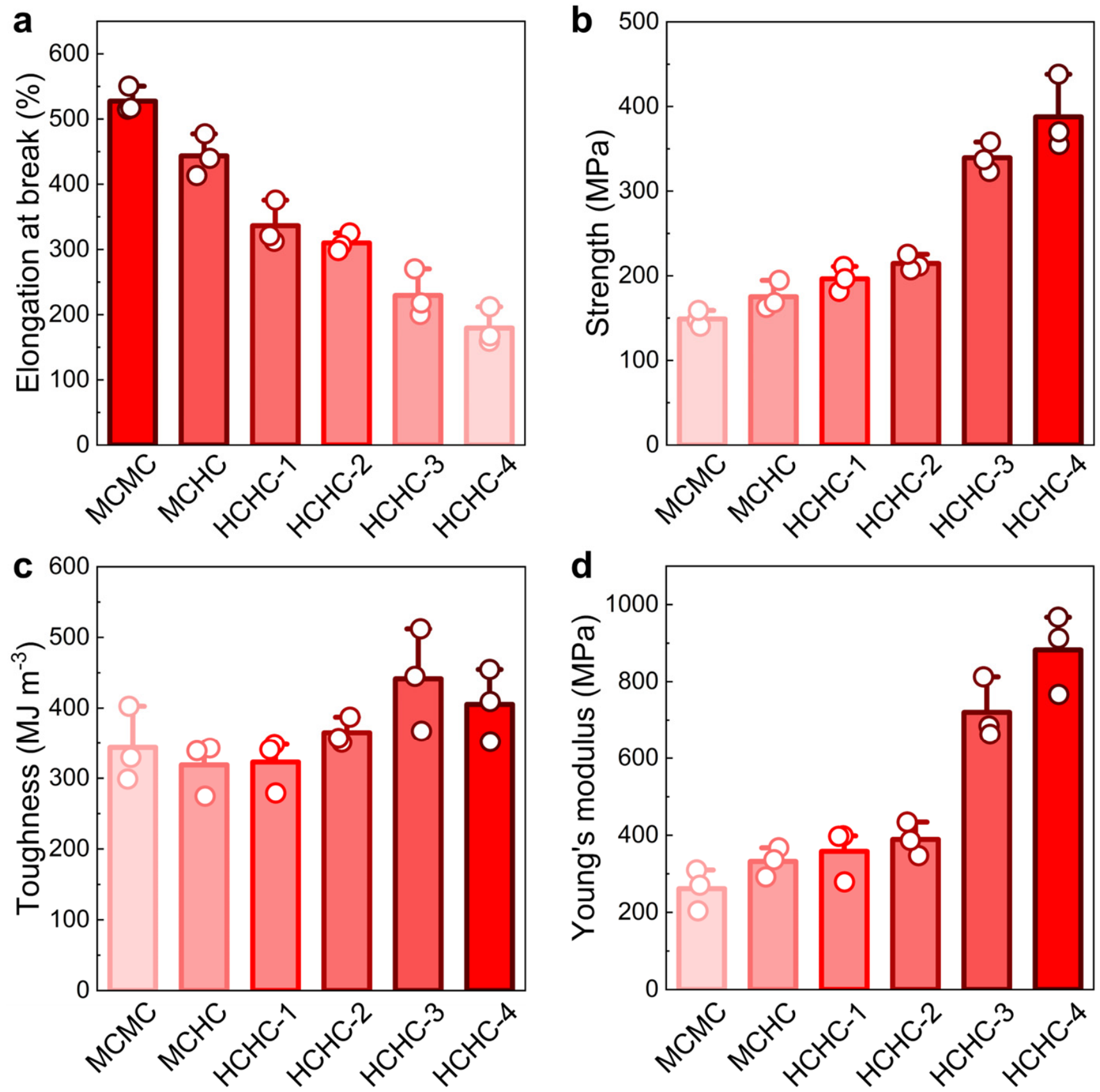

Fig. S20. Elongation at break **(a)**, Tensile strength **(b)**, Toughness **(c)** and Young's modulus **(d)** of microstates between MCMC and HCHC. The polymer used was $C_{400}O_{1818}C_{400}$. Bars show mean values, error bars represent s.d., circles indicate individual measurements (n = 3 independent measurements).

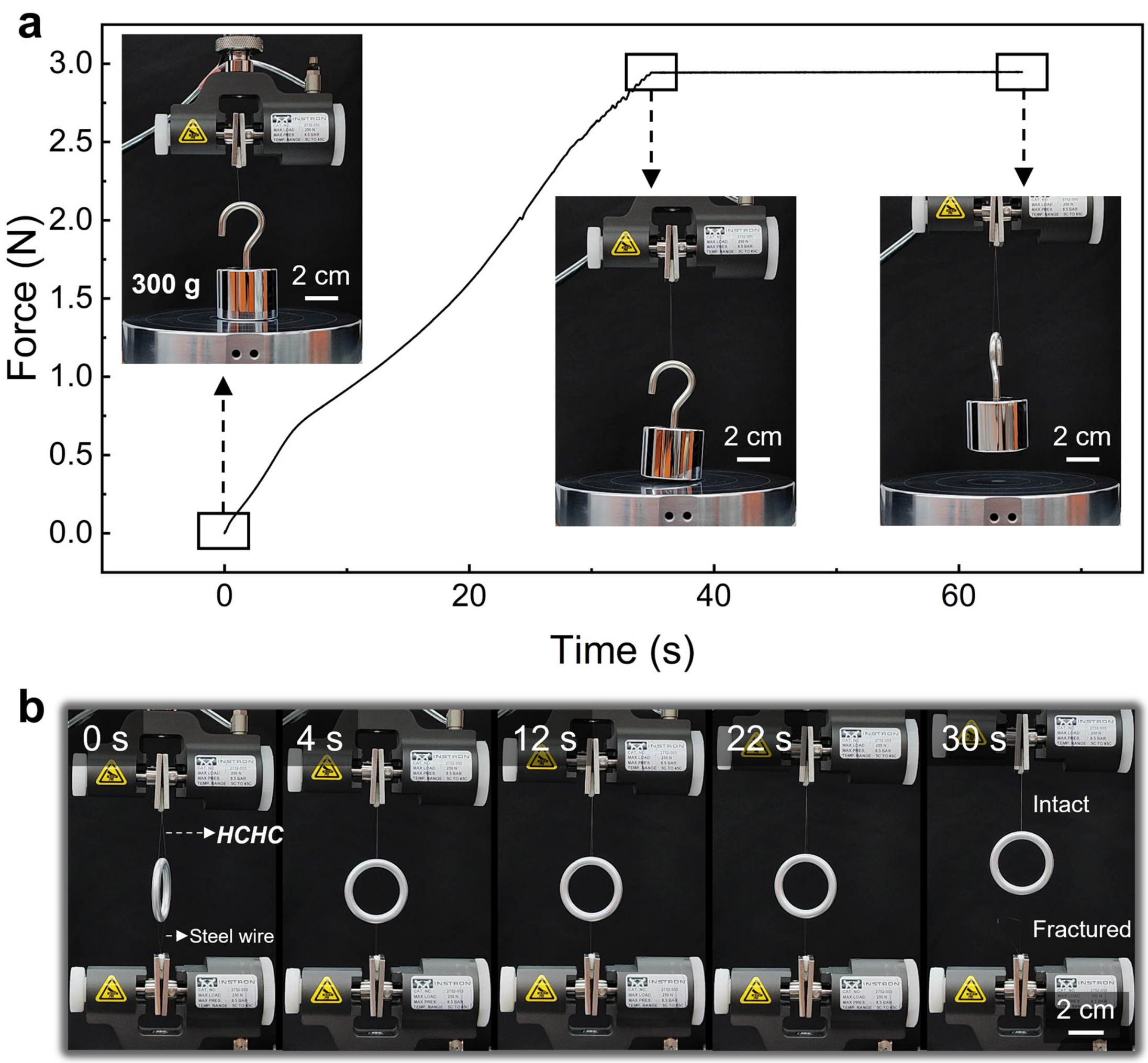

Fig. S21. (a) 0.3 mg of HCHC with diameter of 76 μm can lift a weight of 300 g, which is over one million times of the fiber's own mass. **(b)** Pictures of HCHC fractured stainless steel wire in a direct tensile test against each other. The polymer used was $C_{400}O_{1818}C_{400}$. HCHC and stainless-steel fiber were both 10 cm in length and 0.3 mg in weight.

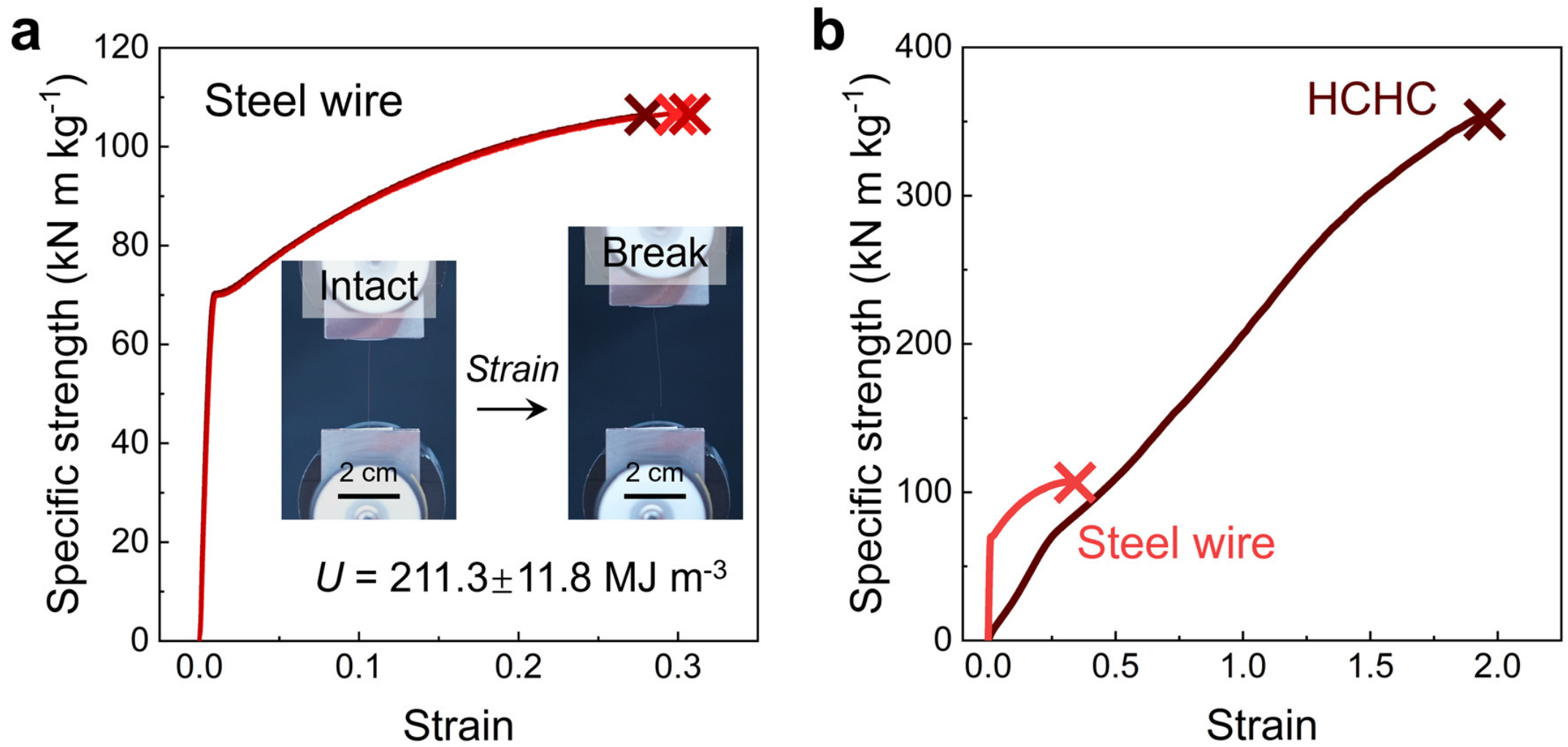

Fig. S22. (a) Specific strength of SUS304 stainless steel microwire. **(b)** Comparison of specific strength for SUS304 stainless steel microwire with HCHC ($C_{400}O_{1818}C_{400}$). Density for SUS304 stainless steel microwire is 7.94 g cm^{-3}, and for HCHC is 1.14 g cm^{-3}.

7 Necking and impact damping characterizations

Fine-tuning necking behaviors

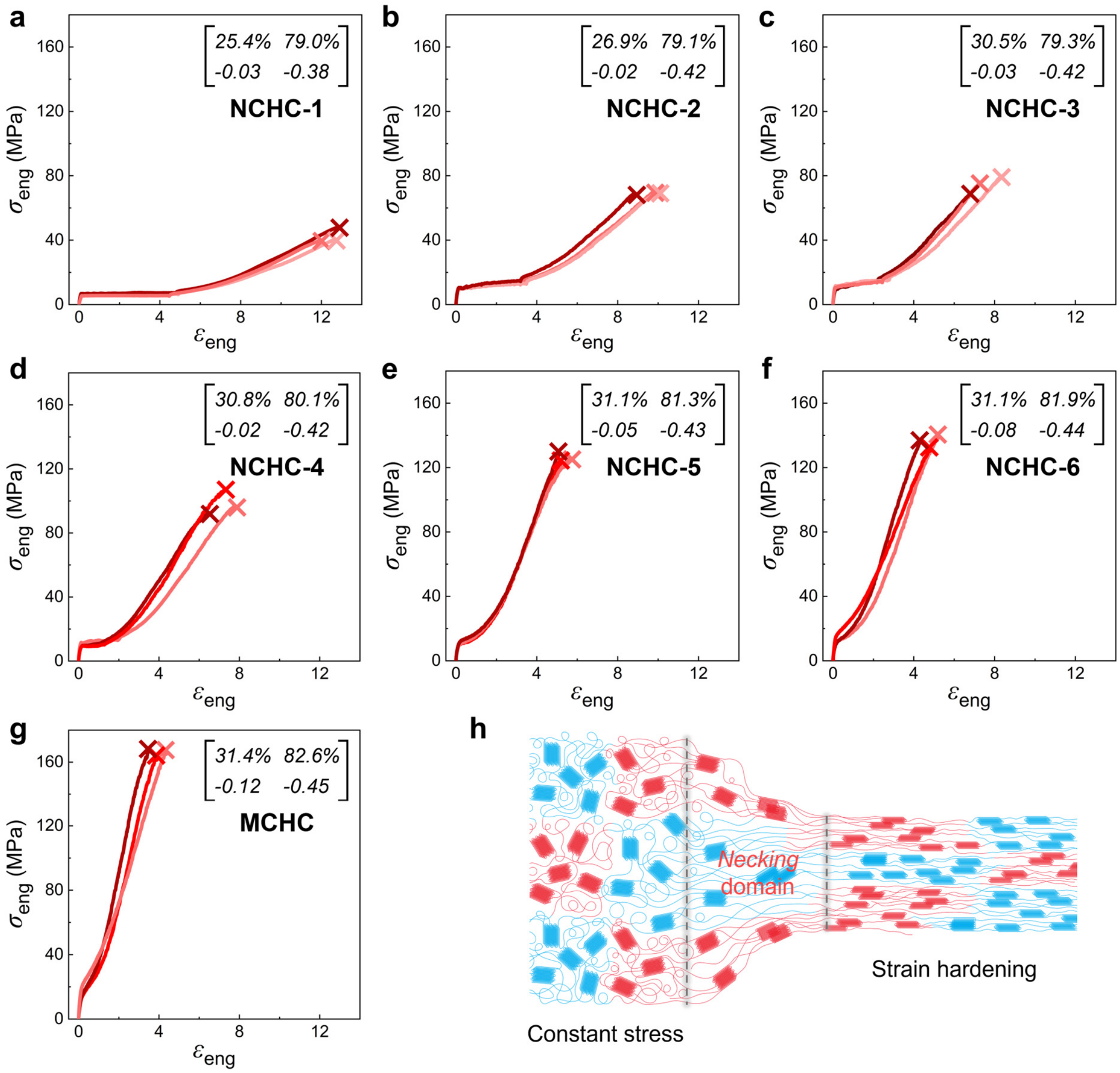

Fig. S23. (a–g) Necking behaviors of microstates between NCHC and MCHC ($C_{400}O_{1818}C_{400}$). Strain rate is 1 min^{-1}. **(h)** The schematic of necking behavior.

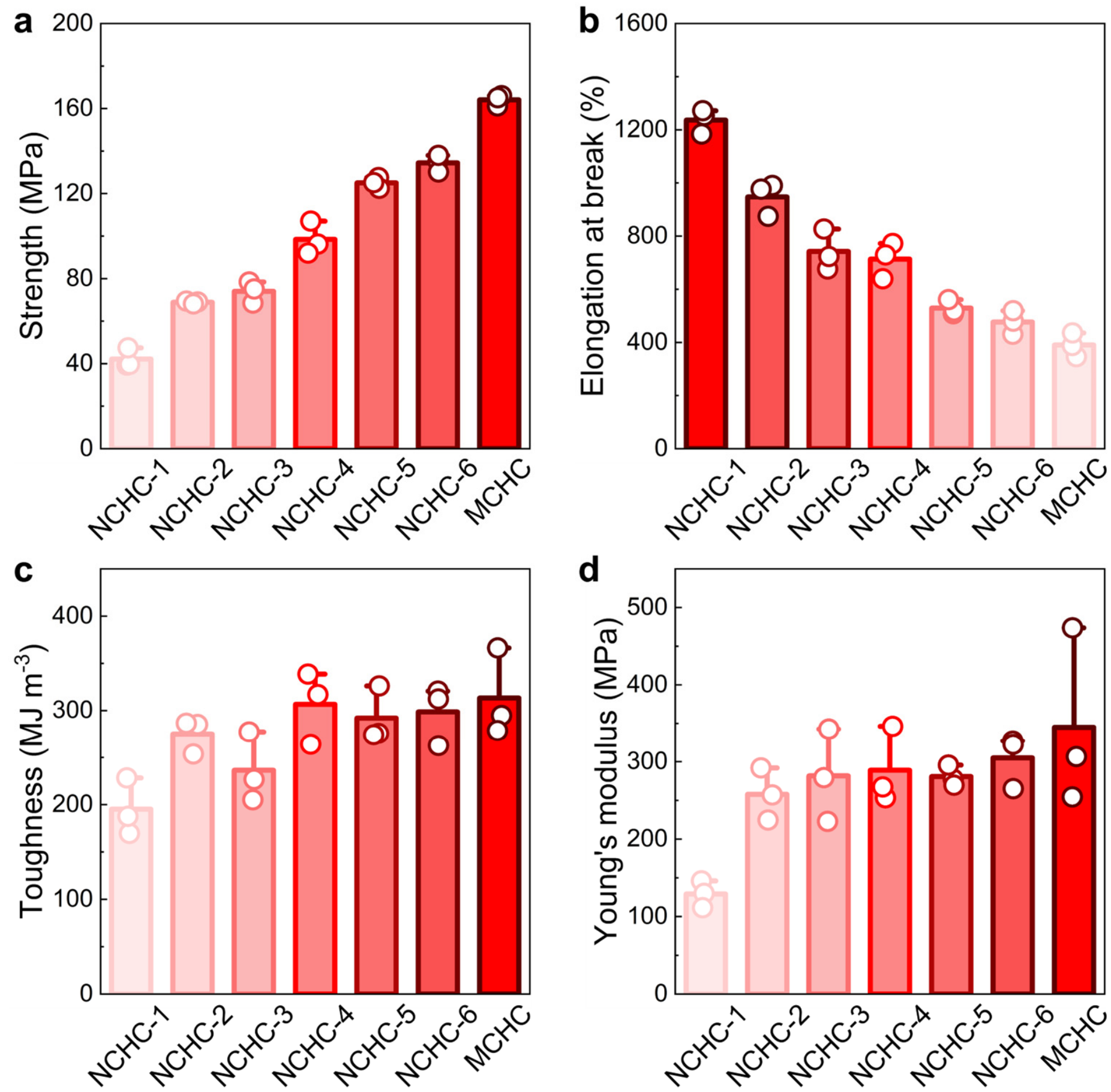

Fig. S24. Tensile strength **(a)**, elongation at break **(b)**, toughness **(c)** and Young's modulus **(d)** of microstates between NCHC and MCHC. The polymer used was $C_{400}O_{1818}C_{400}$. Bars show mean values, error bars represent s.d., circles indicate individual measurements (n = 3 independent measurements).

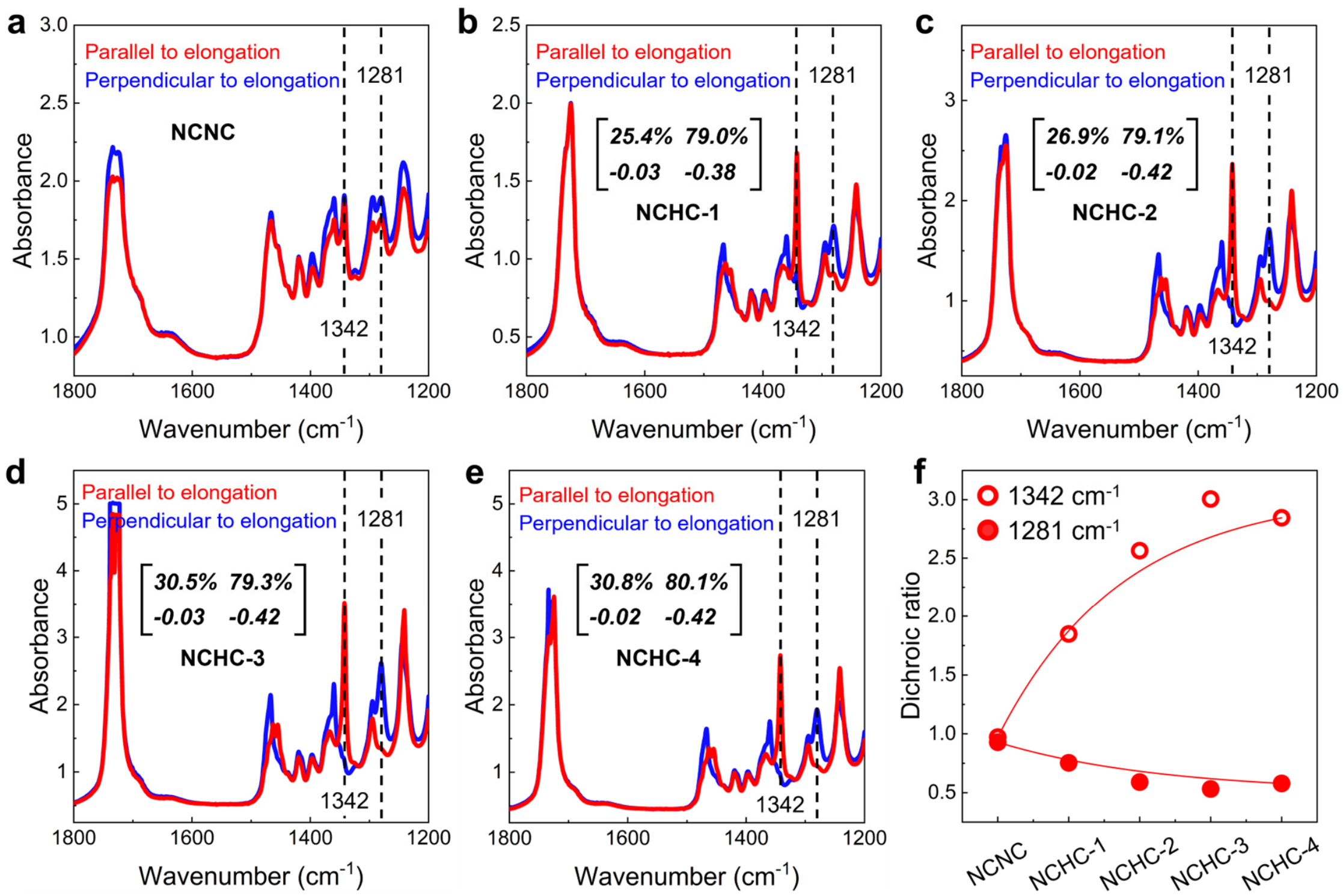

Fig. S25. Polarized FT-IR spectra for NCNC **(a)** and NCHC **(b–e)**. The red and blue lines represent electric vectors parallel and perpendicular to elongation direction. For isotropic state, band intensities of PEO at 1,342 cm^{-1} 1,281 cm^{-1} are similar in both polarizations. **(f)** Chain orientation (Dichroic ratio) measured by polarized FT-IR spectroscopy. Dichroic ratios of PEO bands at 1,342 cm^{-1} (parallel band) 1,281 cm^{-1} (perpendicular band) increased and decreased, respectively, with elongation, while the four parameters in matrix are similar.

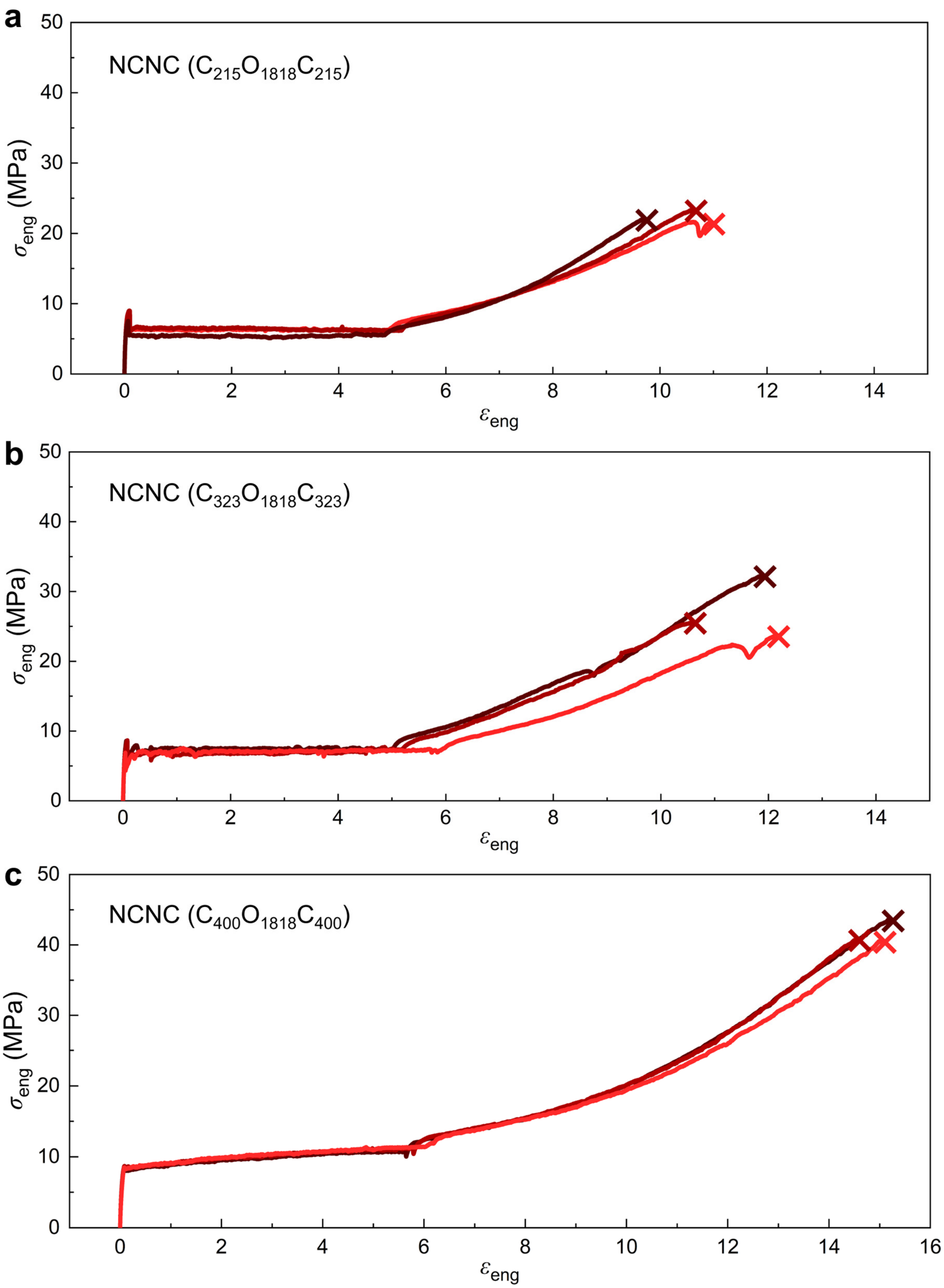

Fig. S26. (a–c) Tensile stress–strain curves of NCNC with necking behaviors using polymers with different hydrophobic block ratios.

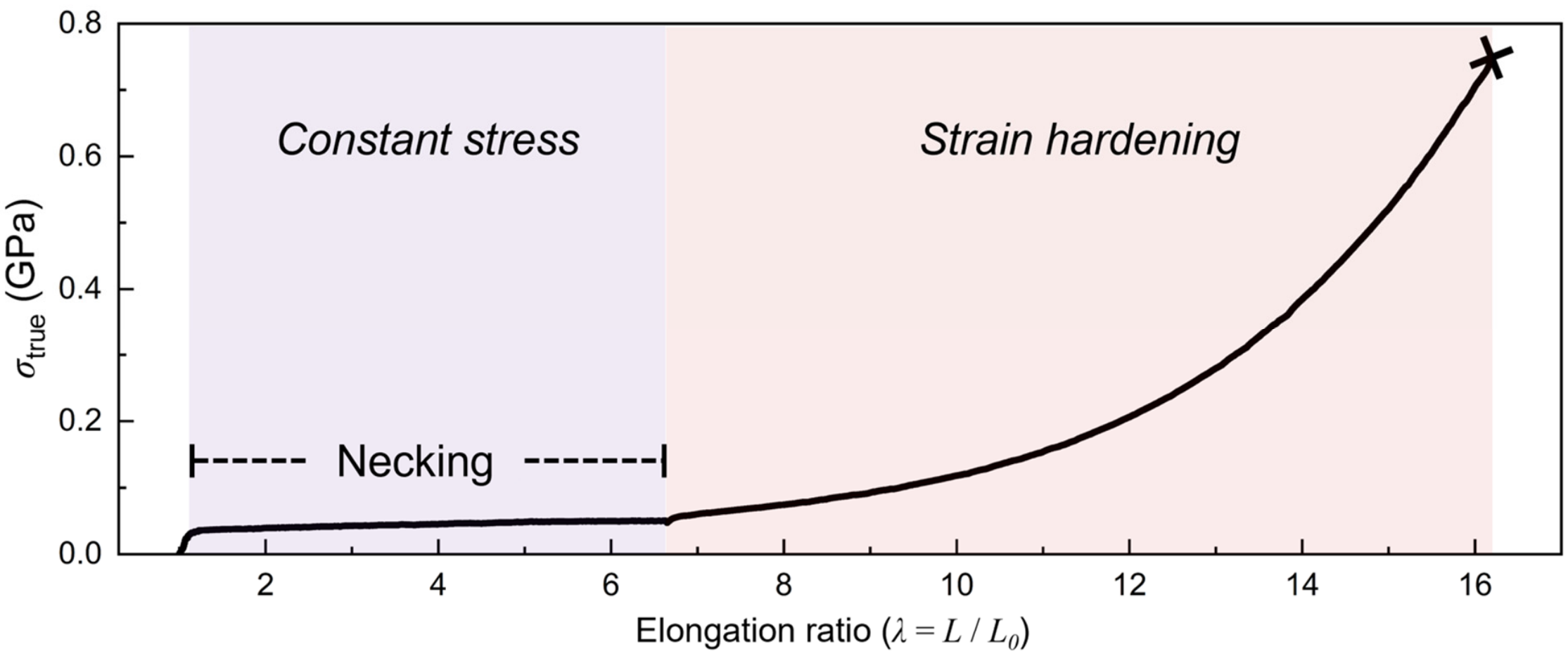

Fig. S27. True stress (σ_{true}) vs elongation ratio ($\lambda = L / L_0$) curves of NCNC state ($C_{400}O_{1818}C_{400}$).

POM and SEM characterization of necking deformation

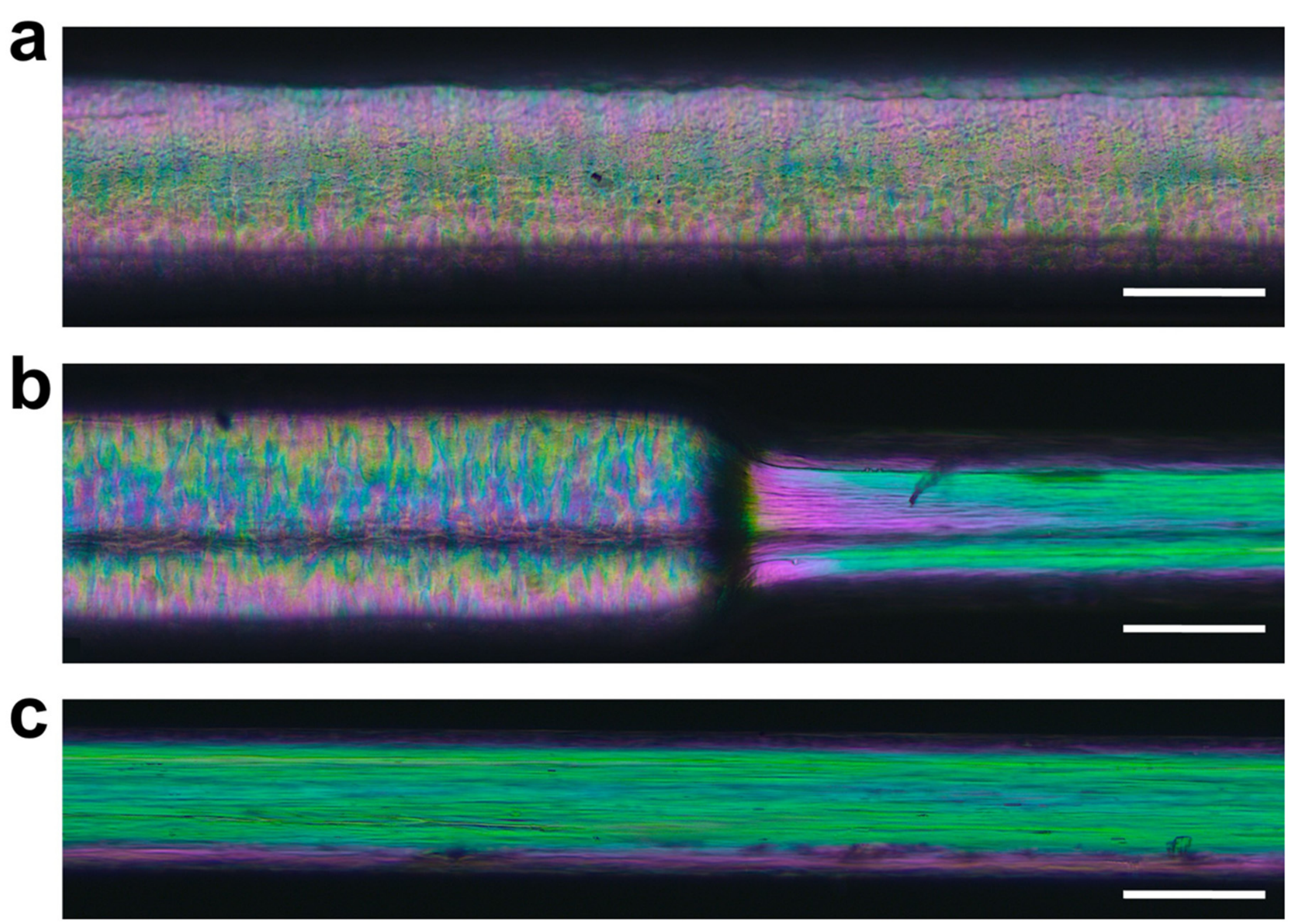

Fig. S28. Polarizing microscope images of NCNC **(a)** before necking, **(b)** partially necking, and **(c)** after necking. The scale bar is 100 μm. Representative POM images from n = 3 independent measurements showing similar results.

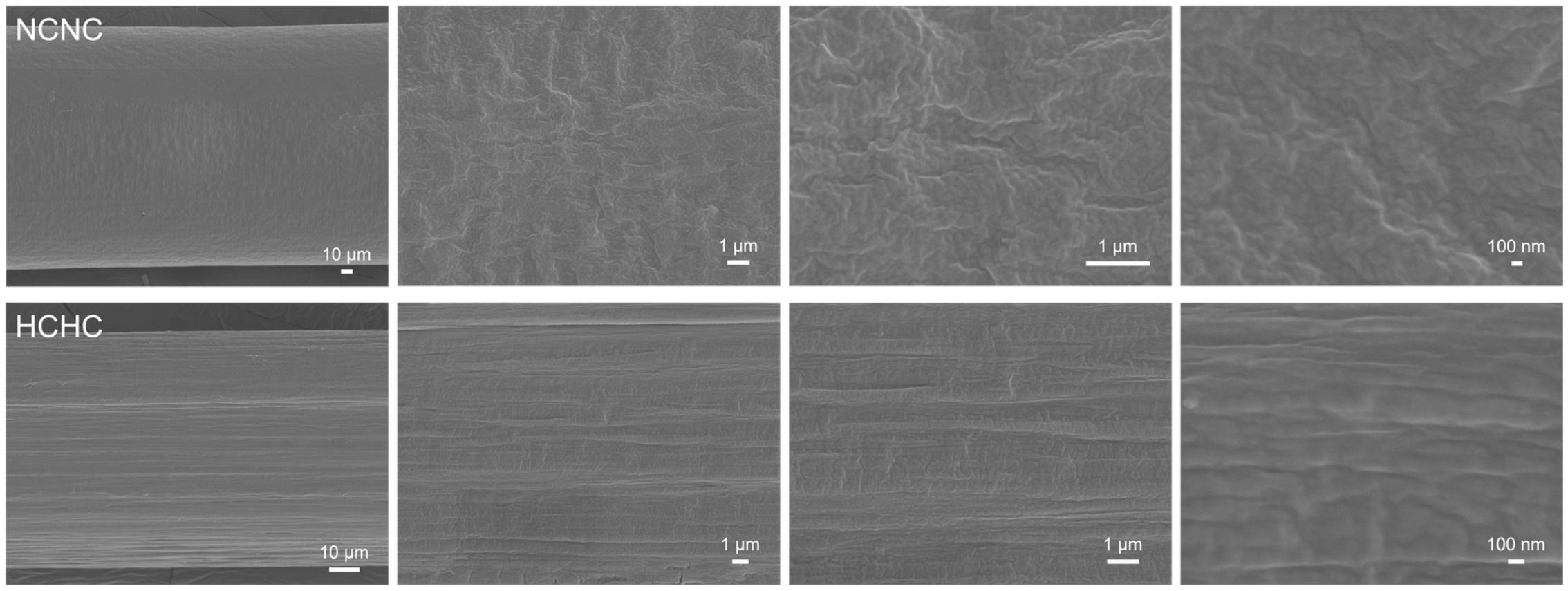

Fig. S29. SEM images of NCNC and HCHC. The surface texture of NCNC exhibits isotropic characteristics, whereas that of HCHC demonstrates a highly oriented fibril-like structure along the elongation direction. Representative SEM images from n = 3 independent measurements showing similar trends.

Impact damping performance of MMC

To test impact damping performance, one end of the fiber was fixed to the force sensor of a load frame. A certain weight was attached to the other end of the fiber and dropped from the height of testing. The force response was recorded by the sensor to evaluate the force damping performance. The damping ratio was calculated using the following equation:

$$D = \frac{F_{\mathrm{ref}} - F_{\mathrm{sample}}}{F_{\mathrm{ref}} - F_{\mathrm{g}}} \times 100\%$$

where F_{ref} and F_{sample} are the maximum force recorded by the sensor during the testing process, F_{g} is static gravity of weight, which was calculated using $F_{\mathrm{g}} = m\mathrm{g}$, with m being the mass of the dropping weight and g being acceleration due to gravity (9.81 m/s^2). In a typical test, a weight of 25 g and a dropping height of 5 cm or 15 cm were used. HDPE was selected as reference sample.

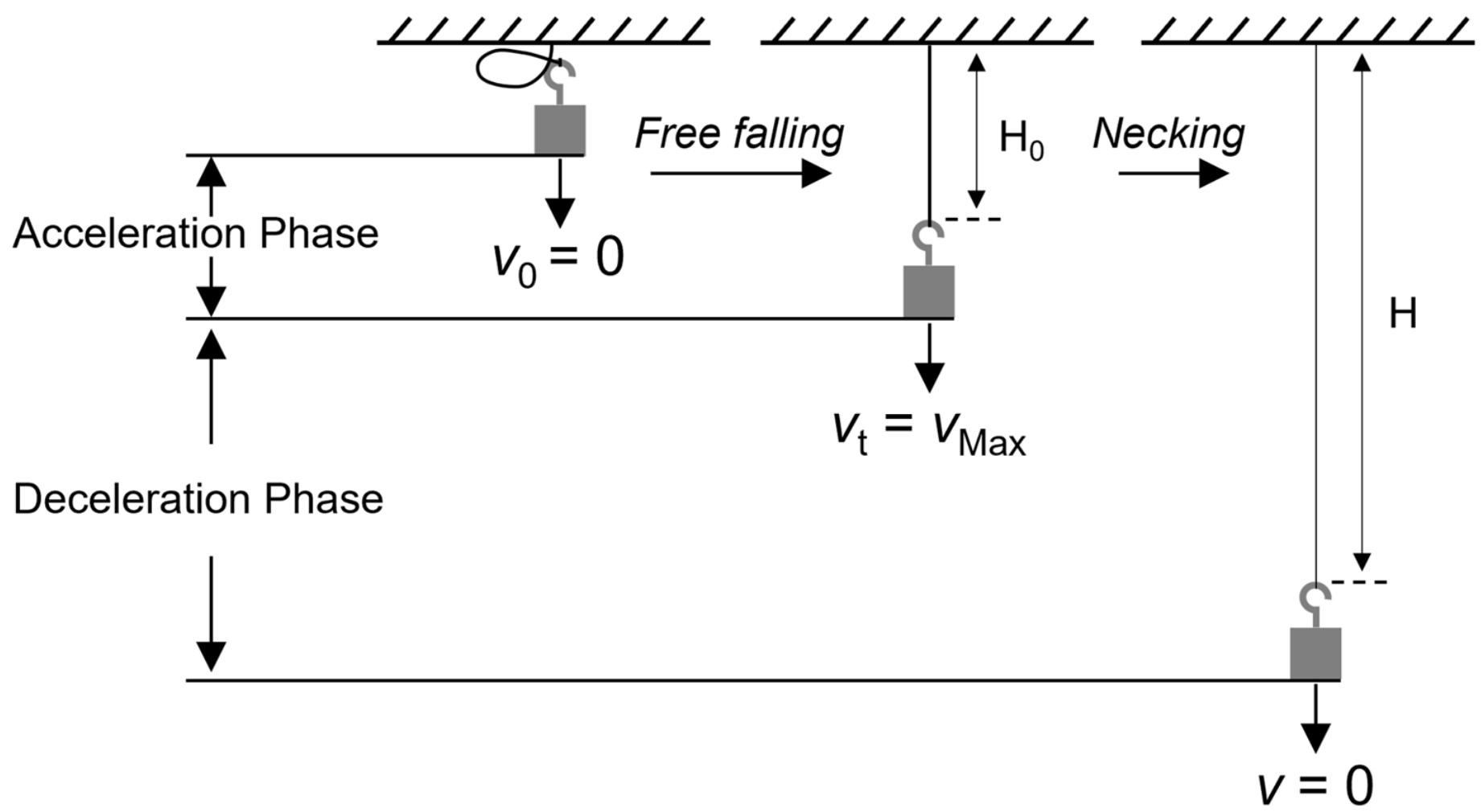

Fig. S30. Characterization of impact force damping.

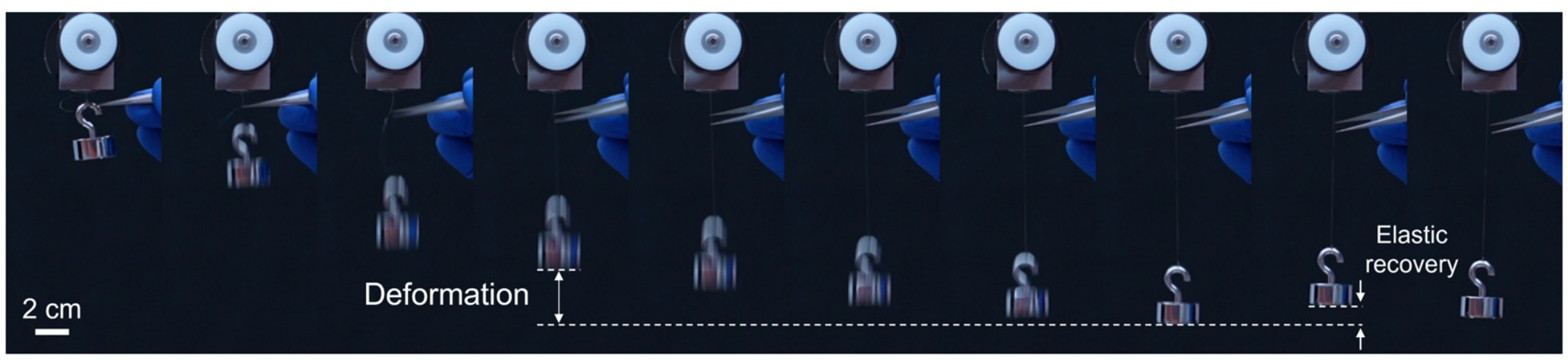

Fig. S31. Impact force damping of HCHC ($C_{400}O_{1818}C_{400}$) with a dropping weight of 25 g and dropping height of 5 cm.

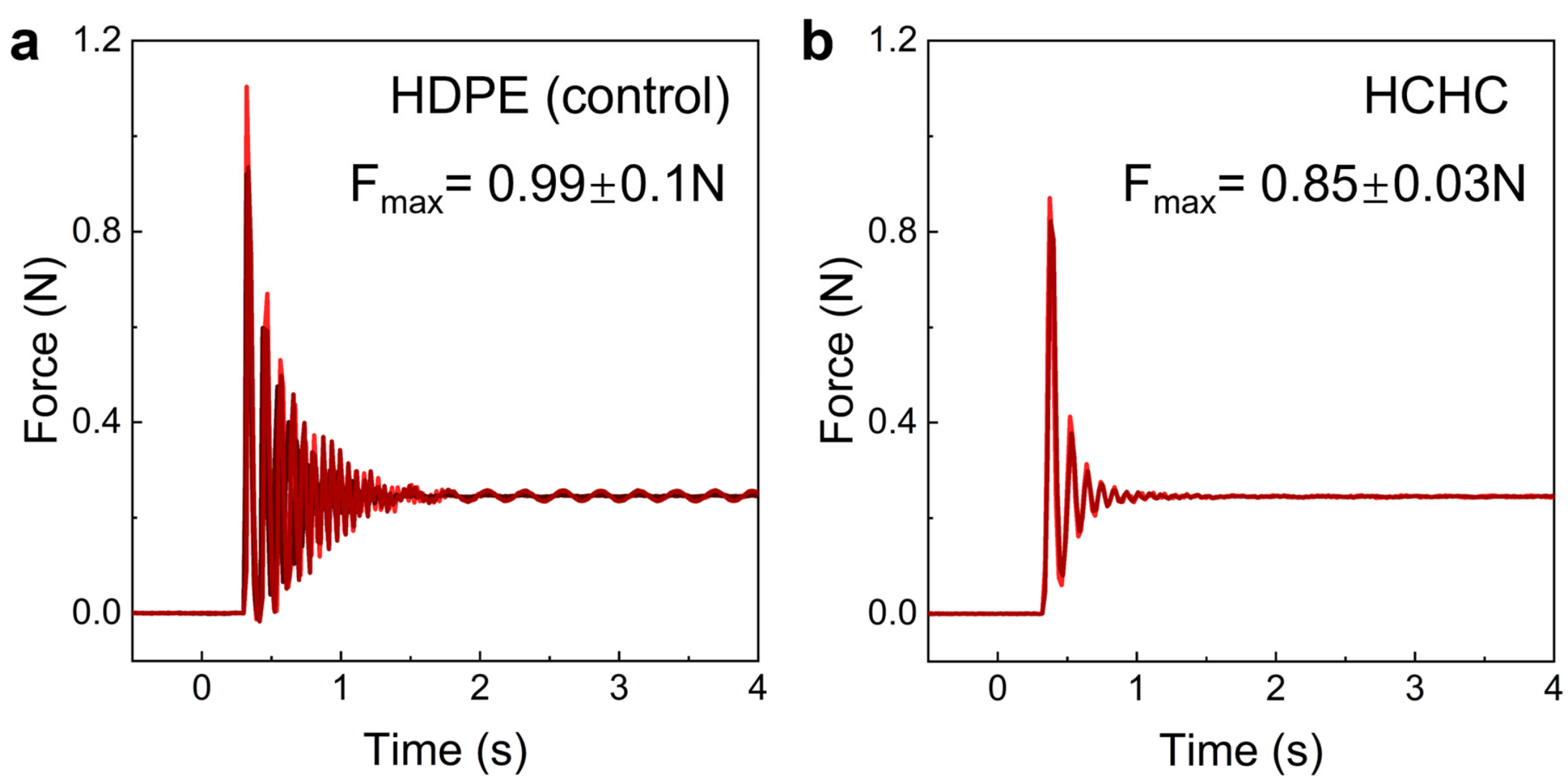

Fig. S32. Force–time curves of **(a)** High-density polyethylene (HDPE) and **(b)** HCHC ($C_{400}O_{1818}C_{400}$) under impact loading with a dropping weight of 25 g and dropping height of 5 cm.

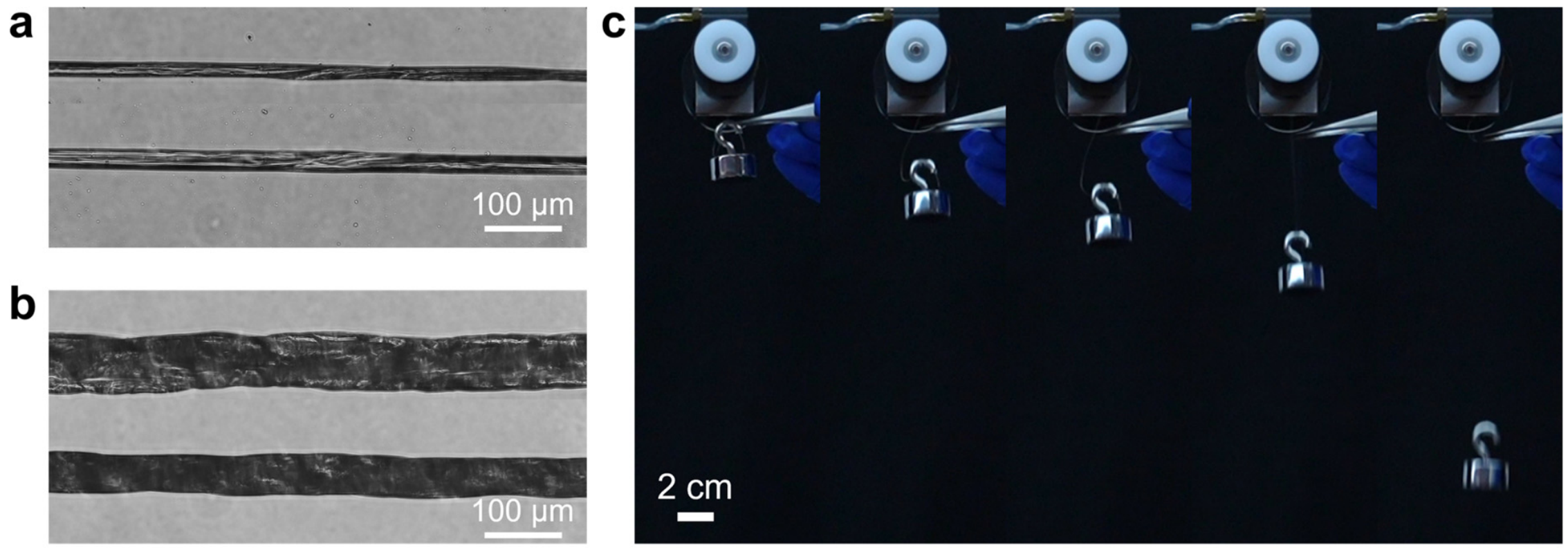

Fig. S33. Impact force damping of ultra-high-molecular-weight polyethylene (UHMWPE). **(a, b)** Bright-field optical microscopy image of UHMWPE monofilaments and twisted yarn (prepared using 7 monofilament). **(c)** UHMWPE was broken by the impact of a 25-gram weight drop from a height of 5 cm.

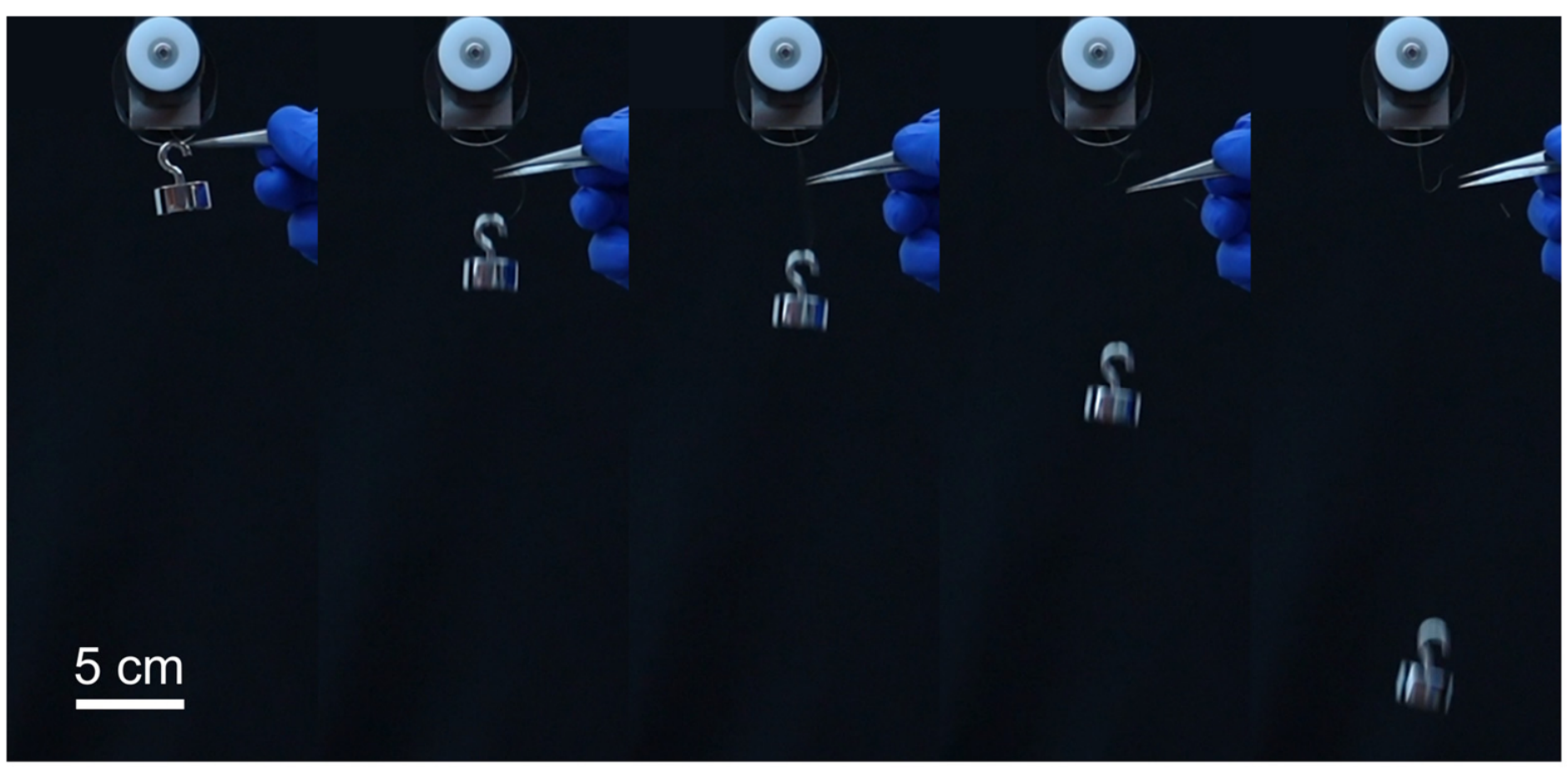

Fig. S34. Kevlar fiber was broken by the impact of a 25-gram weight drop from a height of 5 cm.

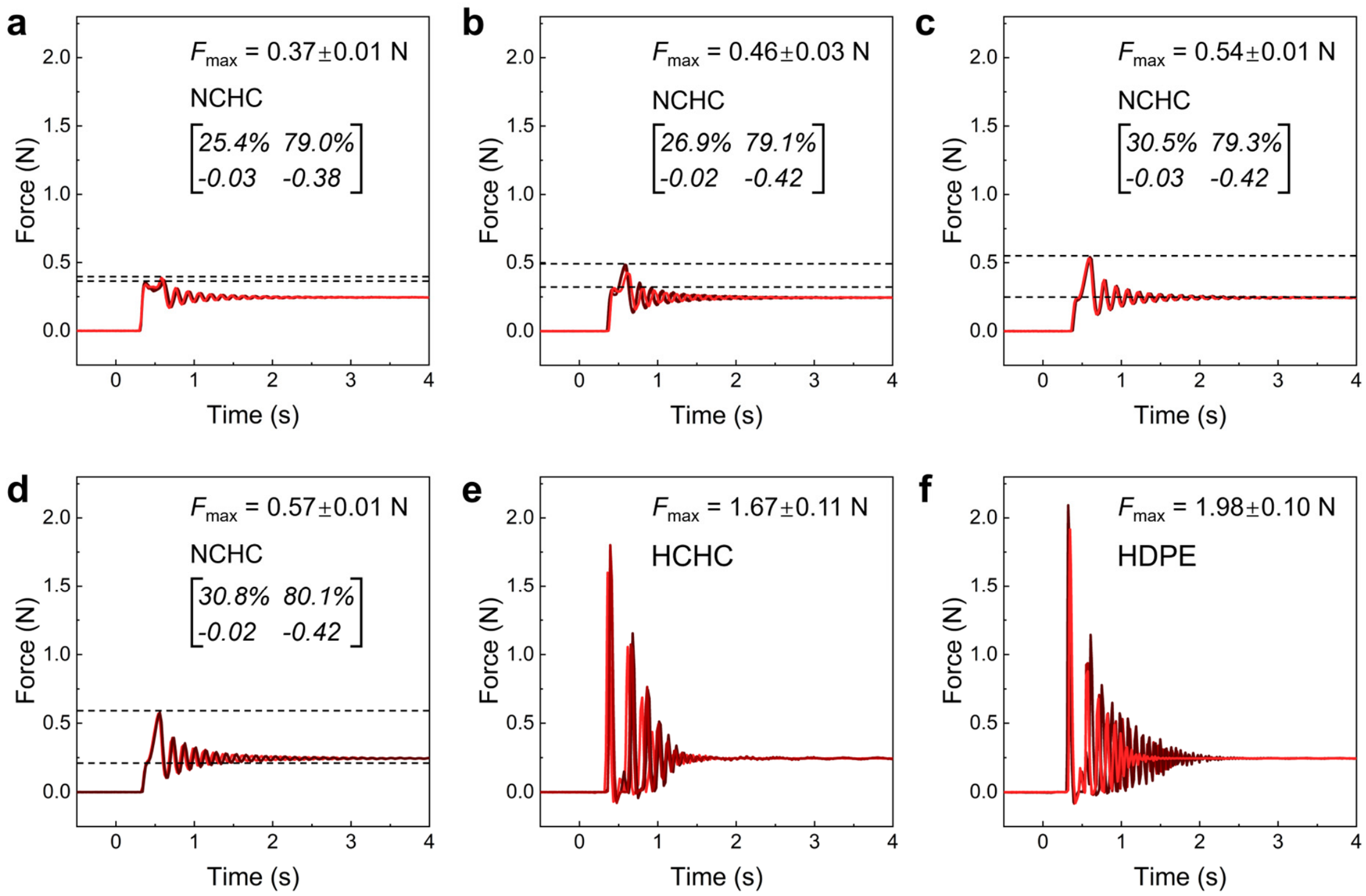

Fig. S35. Impact force damping of microstates between NCHC and HCHC compared with High-density polyethylene (HDPE). **(a–d)** Force–time curves of NCHC with different matrix parameters under impact loading. **(e)** Force–time curves of HCHC under impact loading. **(f)** Force–time curves of HDPE under impact loading. The dropping weight was 25 g and dropping height was 15 cm. The total length of the testing fiber was 5 cm, consisting of a 2 cm segment of NCHC serving as the buffering zone and a 3 cm segment of HCHC as the connecting section. The polymer used was $C_{400}O_{1818}C_{400}$.

8. Actuation behaviors of MMC

Thermo-responsive behaviors of PEO and PCL crystal

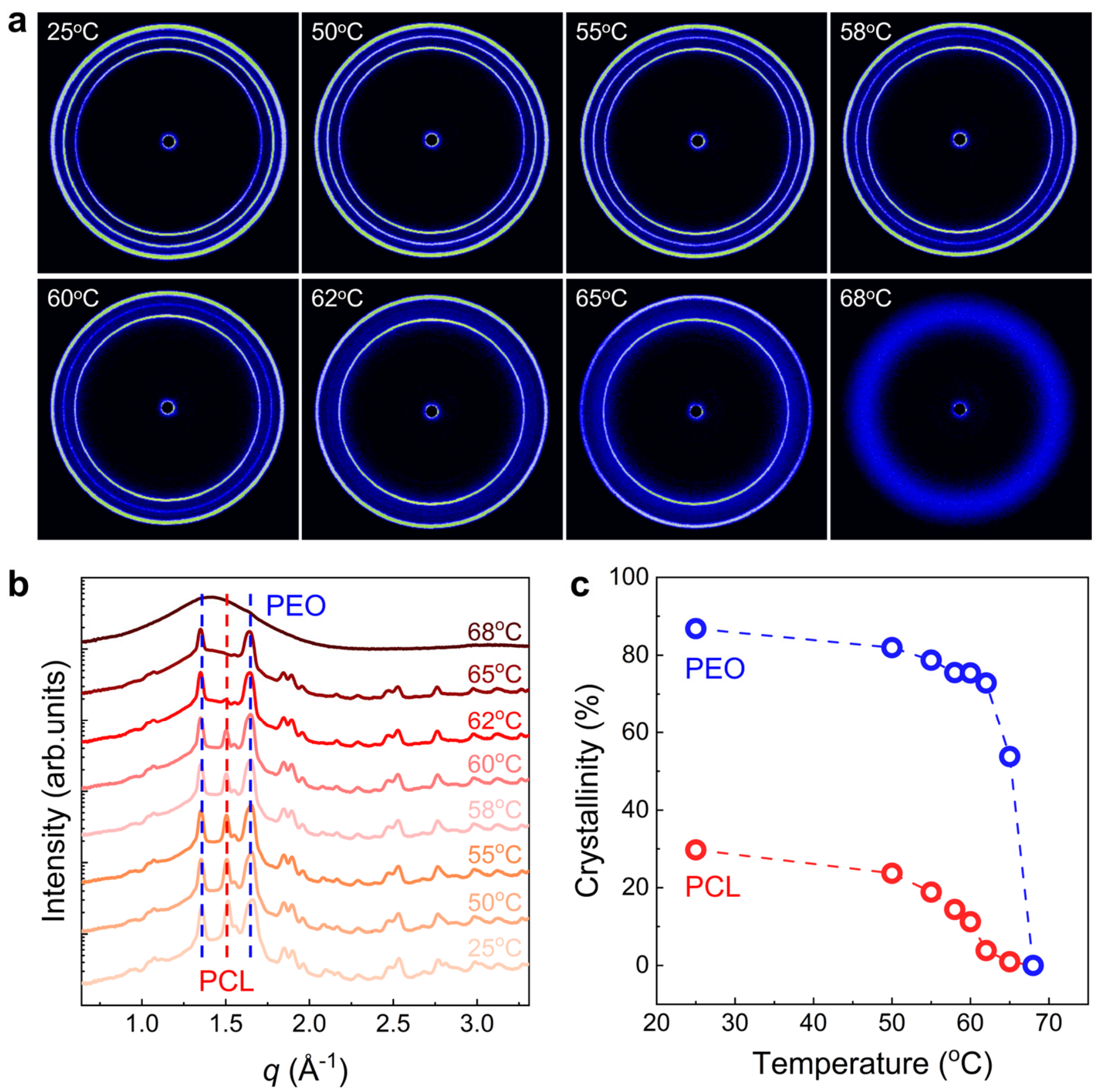

Fig. S36. WAXS characterization of crystal scattering peaks from PCL and PEO during heating. **(a)** Two-dimensional WAXS plot of NCNC during heating. **(b)** One-dimensional WAXS profile of *NCNC* state at increased temperature show that the crystallinity of PCL and PEO decreased, while the crystal structure remained the same. **(c)** Crystallinity calculated of both PCL and PEO decreased as temperature increased. The heating rate was 10°C/min.

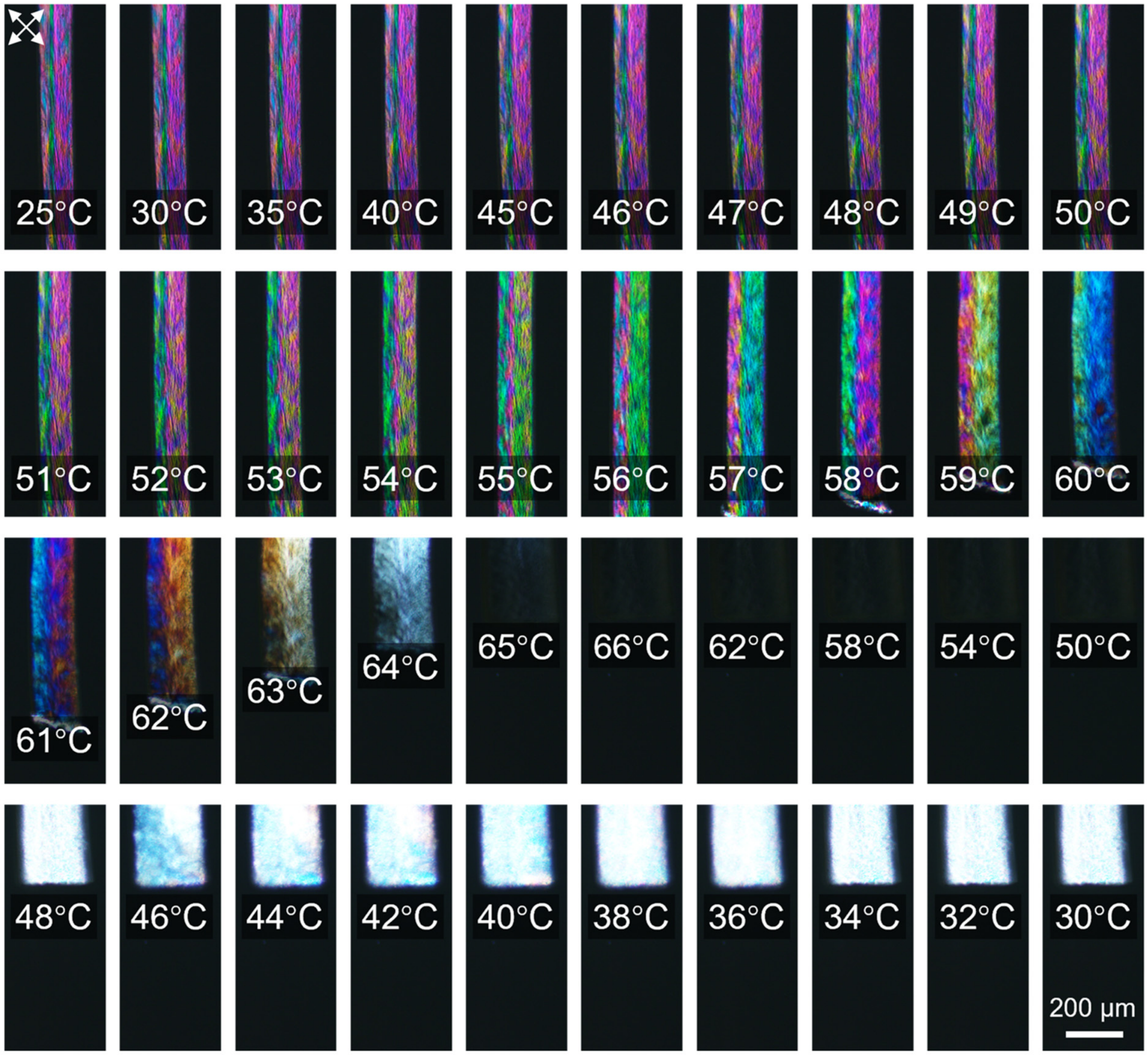

Fig. S37. POM images of HCHC during heating. When the temperature reaches around 58ºC, the crystalline domains begin to melt, inducing contraction of the sample. Representative POM images from n = 3 independent measurements showing similar trends.

Tunable actuation triggered by heat

Actuation: The linear actuation movements of the fibers were quantified using the video analysis and modelling tool, Tracker 5.1.x (The Open-Source Physics Project).(*12*) The hanging weight was marked using a blue tape for movement tracking. The original length of fibers was measured for calibrating in the video footage, and the marked position was analyzed through frame-by-frame tracking. The rotational movement was quantified by frame-by-frame analyzing the video with Tracker 5.1.x. The rotational rate was calculated based on revolutions divided by time. The energy density was then calculated by using the work done by the

fibers divided by the fibers mass.

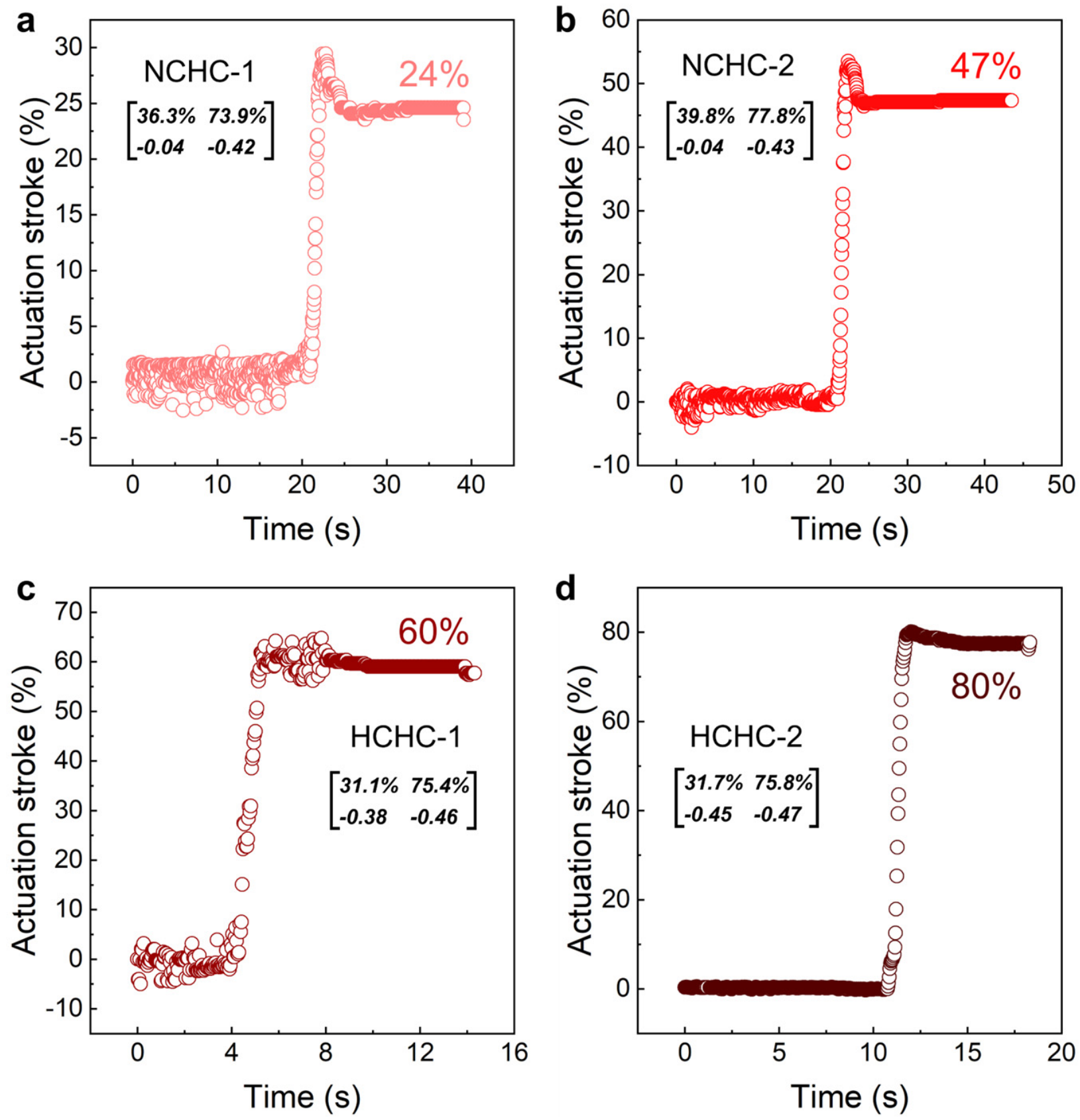

Fig. S38. (a–d) Experimental actuation stroke of microstates between NCHC and HCHC with heat as stimulus ($C_{215}O_{1818}C_{215}$).

Tunable actuation triggered by hydration

The oriented fiber was placed vertically, with the upper end clamped to an iron stand and the lower end attached to a needle marked with a reference point. A graduated cylinder filled with deionized water was then raised from below so that the entire fiber was submerged within ~1 s. Upon hydration, dissolution of the oriented PEO segments drives contraction. The process was video-recorded, and the motion was analyzed using Tracker 5.1.x (The Open-Source Physics Project) to extract the actuation stroke and velocity. The oriented fiber was clamped at both ends in a load frame, and deionized water was then applied until the fiber was fully wetted. The force generated upon hydration was recorded, and the actuation stress was obtained by dividing this force by the initial cross-sectional area of the fiber.

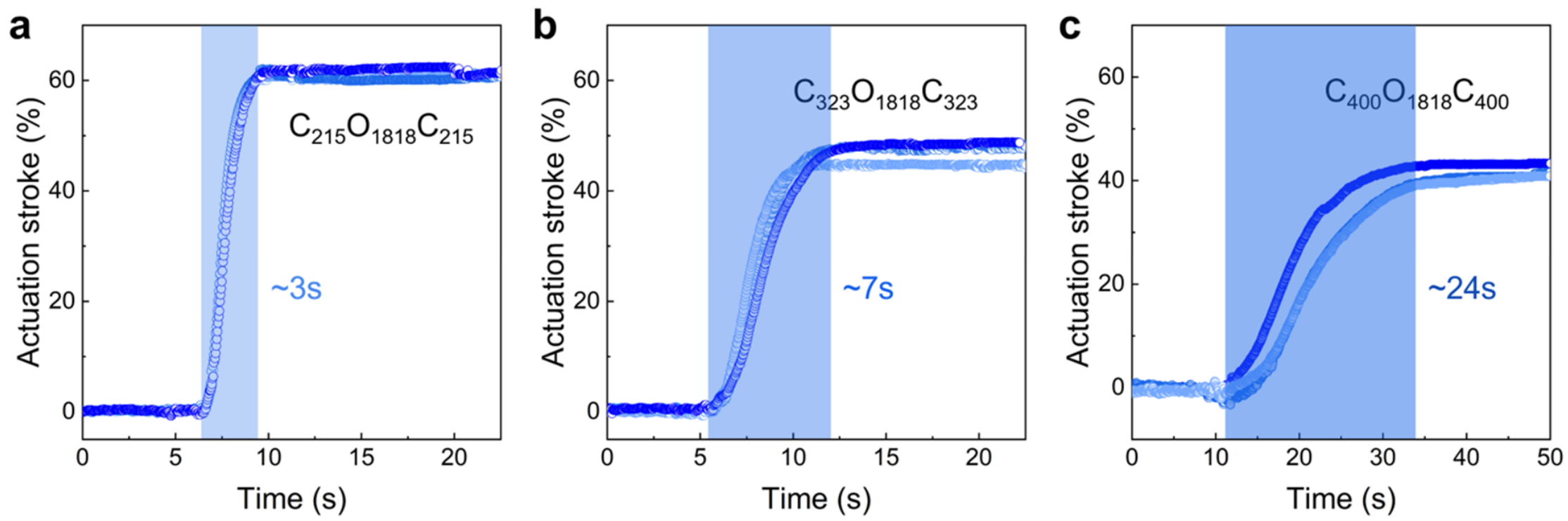

Fig. S39. Actuation performance of HCHC with different hydrophobic block ratios. **(a)** $C_{215}O_{1818}C_{215}$, (b) $C_{323}O_{1818}C_{323}$, (c) $C_{400}O_{1818}C_{400}$. The response speed and actuation stroke decrease with increasing hydrophobic block ratio. The hydration process was implemented through bath immersion, ensuring uniform exposure of the samples to the aqueous environment.

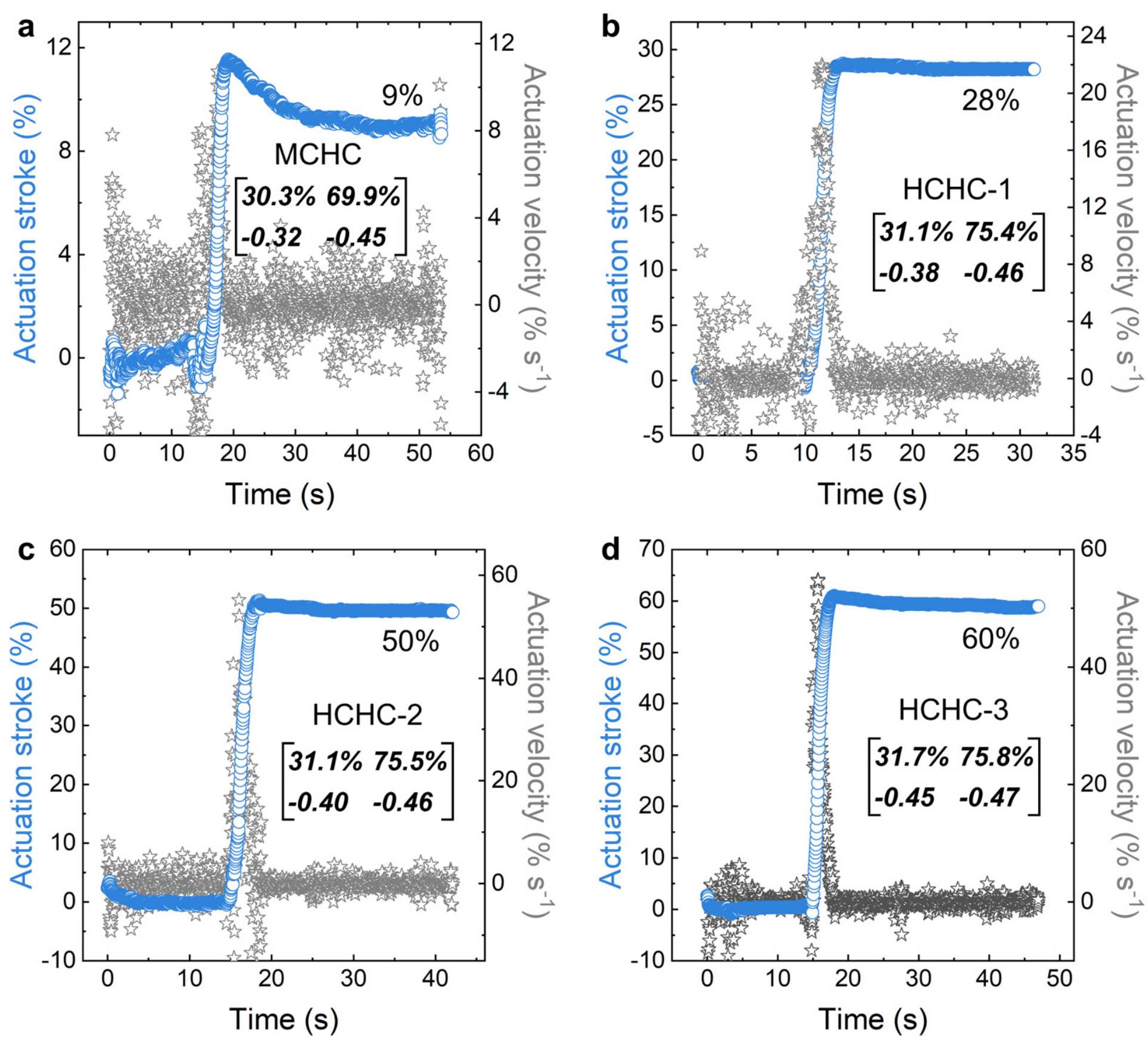

Fig. S40. (a–d) Actuation stroke and velocity of microstates between MCHC and HCHC under hydration ($C_{215}O_{1818}C_{215}$). Bath immersion was employed as the hydration method.

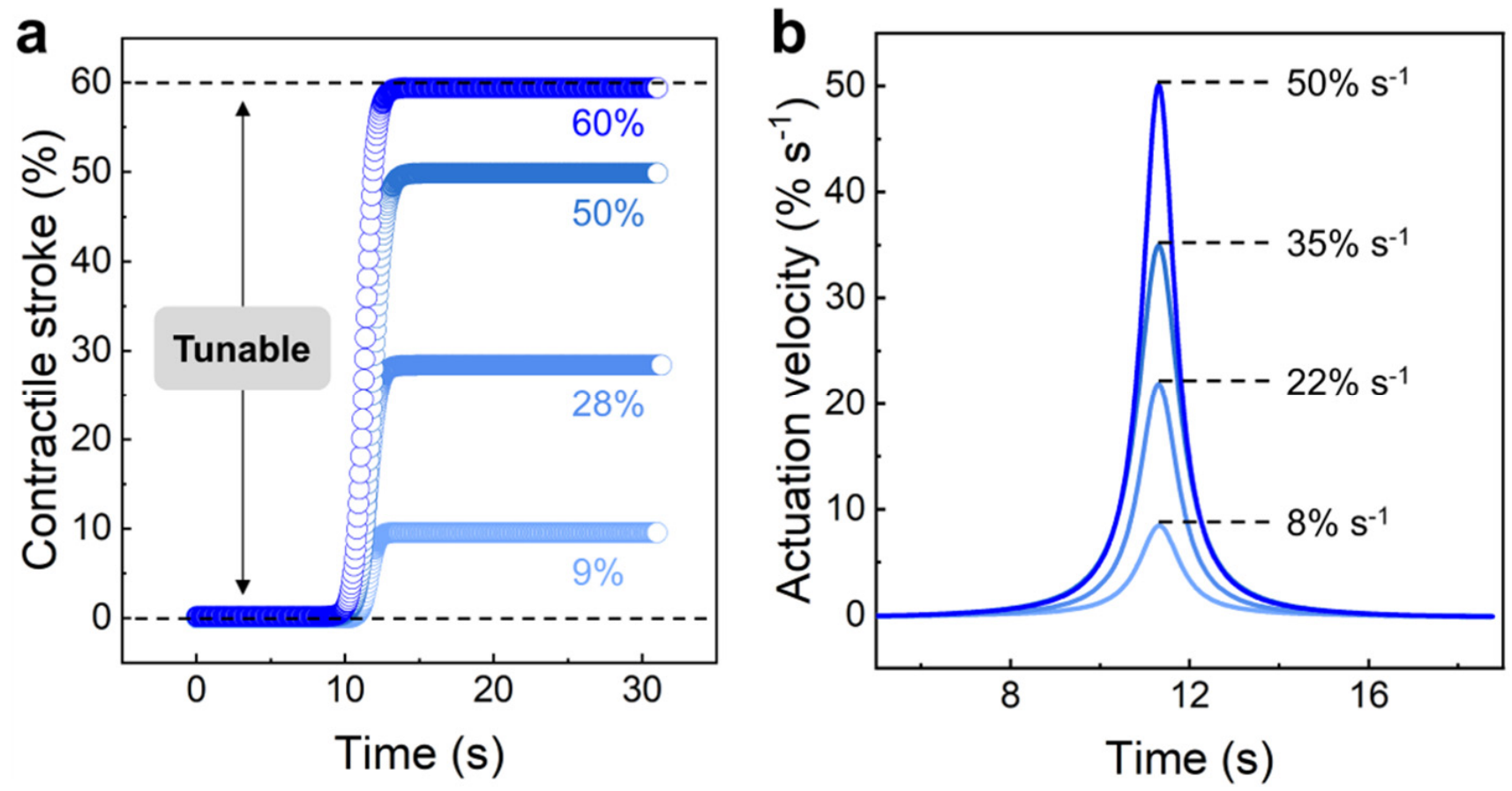

Fig. S41. **(a)** The actuation stroke triggered by hydration was fitted with Boltzmann function to. **(b)** The actuation velocity fitted with Lorentz function. Hydration was carried out by bath immersion.

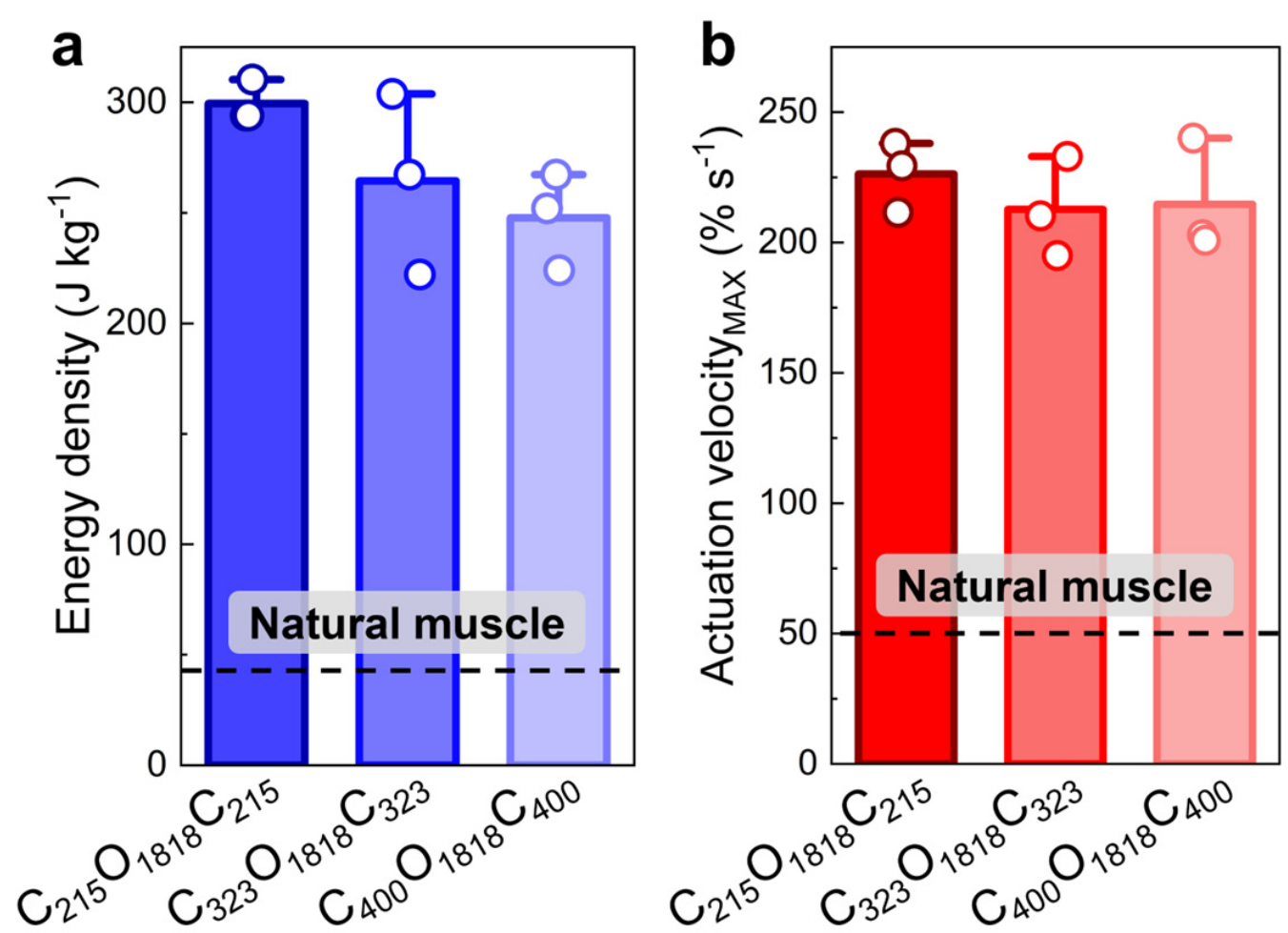

Fig. S42. **(a)** Energy density and **(b)** maximum actuation velocity of HCHC state referenced to natural muscle(*13*). Bars show mean values, error bars represent s.d., circles indicate individual measurements (n = 3 independent measurements).

9. Applications of MMC-based actuators

Self-closure surgical suture

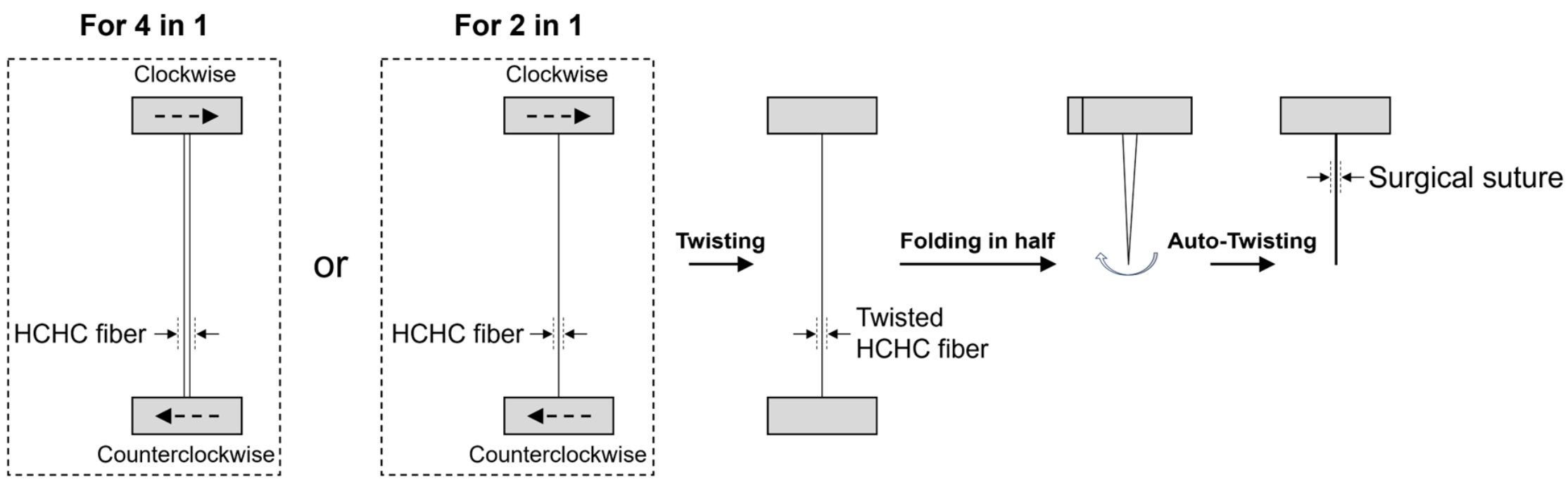

Fig. S43. Fabrication process of surgical sutures.

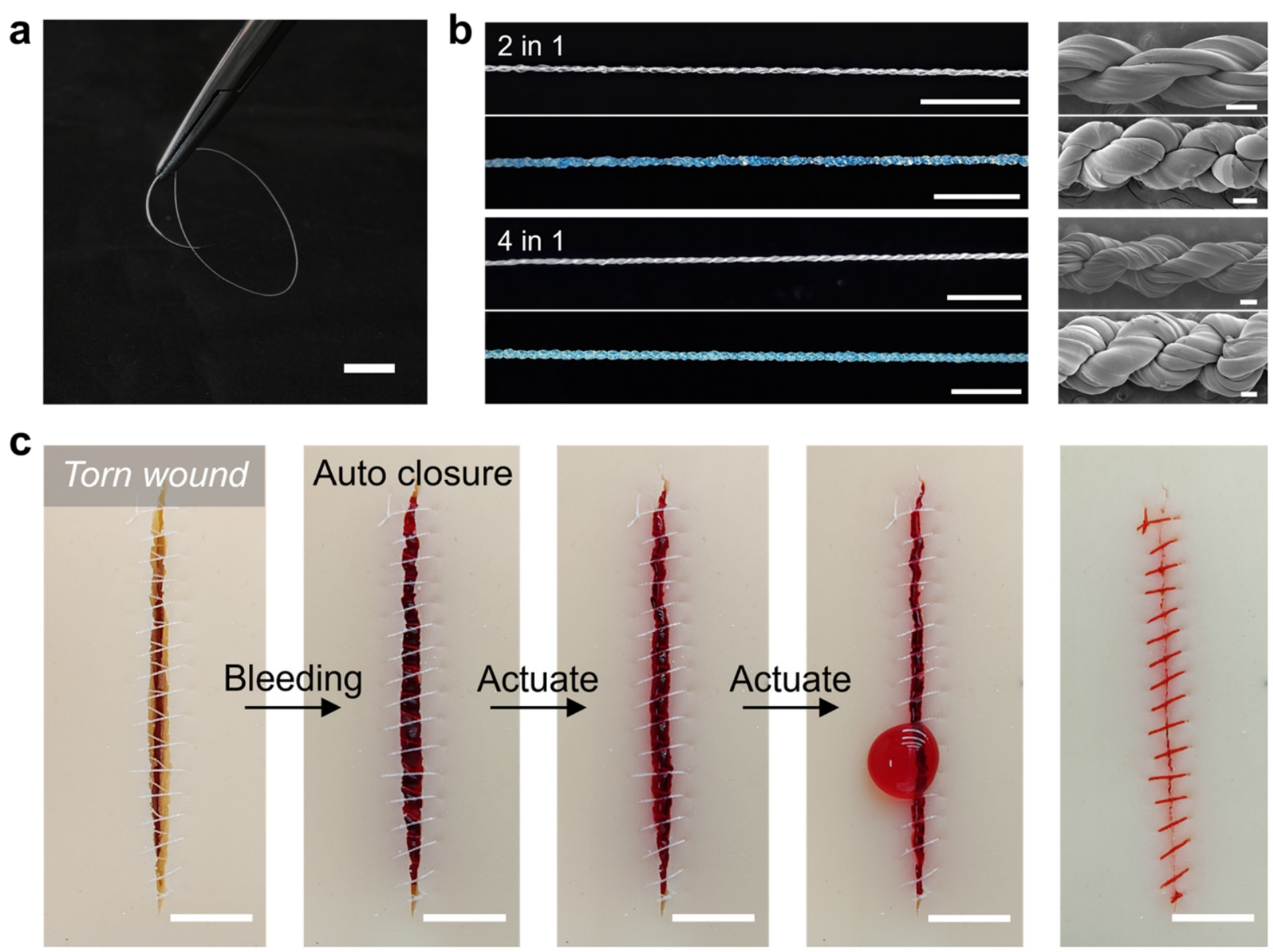

Fig. S44. (a) Image of surgical suture fabricates with HCHC. Scale bar is 1 cm. **(b)** Optical and SEM images of HCHC-based braided sutures, where "2 in 1" and "4 in 1" denote yarns fabricated from two and four fibers, respectively. Hydration-induced transition from HCHC braided yarn (top) to MCAm hydrogel braided yarn (bottom). Scale bar for optical images is 1 cm and for SEM images is 200 μm. Representative SEM images three independent measurements showing similar results. **(c)** Torn wound sutured with HCHC surgical sutures exhibit autonomous closure in response to hemorrhagic stimuli. Scale bar is 1 cm.

Blood-triggered tension-reduce tape

In Fig. S45, we demonstrate an MMC-based intelligent tension-relief patch designed for non-invasive wound closure. The underlying mechanism is similar to that of the intelligent surgical suture shown in Fig. S44, where blood exudate stimulates the fibers to generate contractile force and close the wound. Unlike sutures, however, this patch enables non-invasive closure.

The preparation process is as follows: Five NCNC fibers (each 1 cm in length) were fixed at both ends onto two adhesive patches using glue, as shown in Fig. S45b. During cold drawing, NCNC fibers exhibit tunable high extensibility (Fig. S45c), which allows flexible adjustment according to wound size. Depending on the requirement, part or all of the NCNC fibers can be processed into HCHC fibers. Upon hydration, five HCHC fibers can generate a contractile force of 0.22 N (Fig. S45d). By adjusting the number of fibers, the generated force can be tailored to match the modulus of the surrounding tissue, thereby achieving an ideal closure effect.

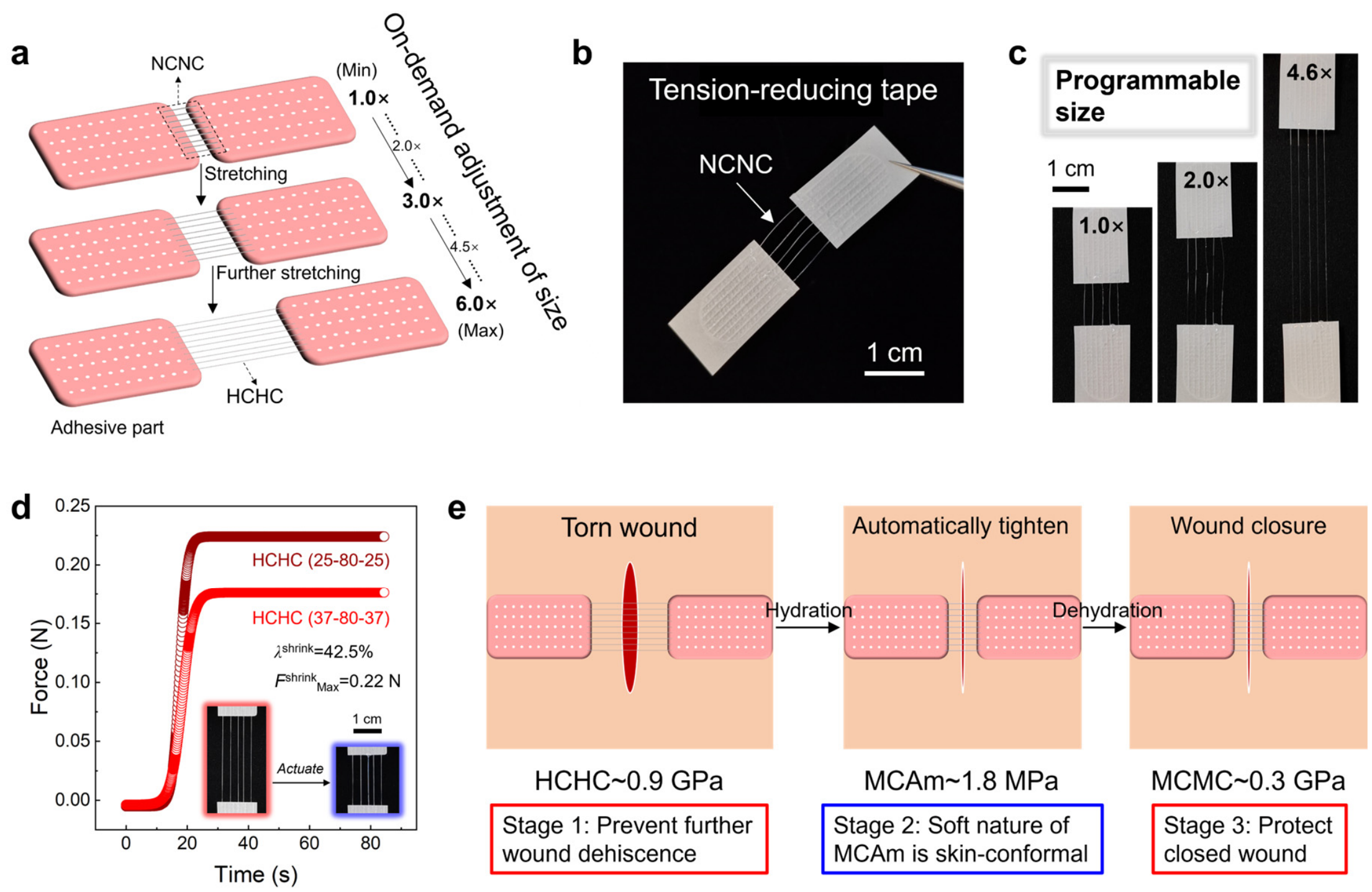

Fig. S45. **(a)** Schematic demonstration of multimodal-responsive tension-reducing tapes with on-demand adjustment of size. **(b)** Image of NCNC state tension-reducing tape. **(c)** NCNC state tension-reducing tape can be programed to different sizes to match various wound dimensions. **(d)** The actuation force of HCHC state tension-reducing tape under hydration. Mist spray was employed as the hydration method. **(e)**

Mechanically adaptive wound closure system. Protection phase: The as-prepared HCHC state tension-reducing tape provided structural reinforcement to resist wound tearing during initial closure. Adaptation phase: upon hydration-triggered actuation, the HCHC state transformed into soft MCAm hydrogel state, fitting tissue morphology without causing secondary injury. Stabilization phase: water evaporation induces reversible transformation to high-modulus MCMC state, maintaining long-term wound apposition and preventing re-opening.

Moisture-responsive fabrics

As a proof-of-concept, we demonstrate an MMC-based smart fabric in which HCHC fibers were sewn onto a commodity textile. To mimic perspiration, water was sprayed onto a flat surface to form a thin liquid layer, and the fabric was then placed in contact with the wetted surface. Upon hydration, the HCHC fibers contracted and lifted the textile locally, transforming the initially flat fabric into a corrugated structure with five regularly spaced ridges aligned with the embedded fibers (Fig. S46b, c). Open channels formed in the valleys between adjacent ridges, and contact with the wetted surface was confined to the ridge tops, reducing the fabric–surface contact area. The resulting ventilation channels between the fabric and the skin would be expected to enhance local airflow and heat dissipation.

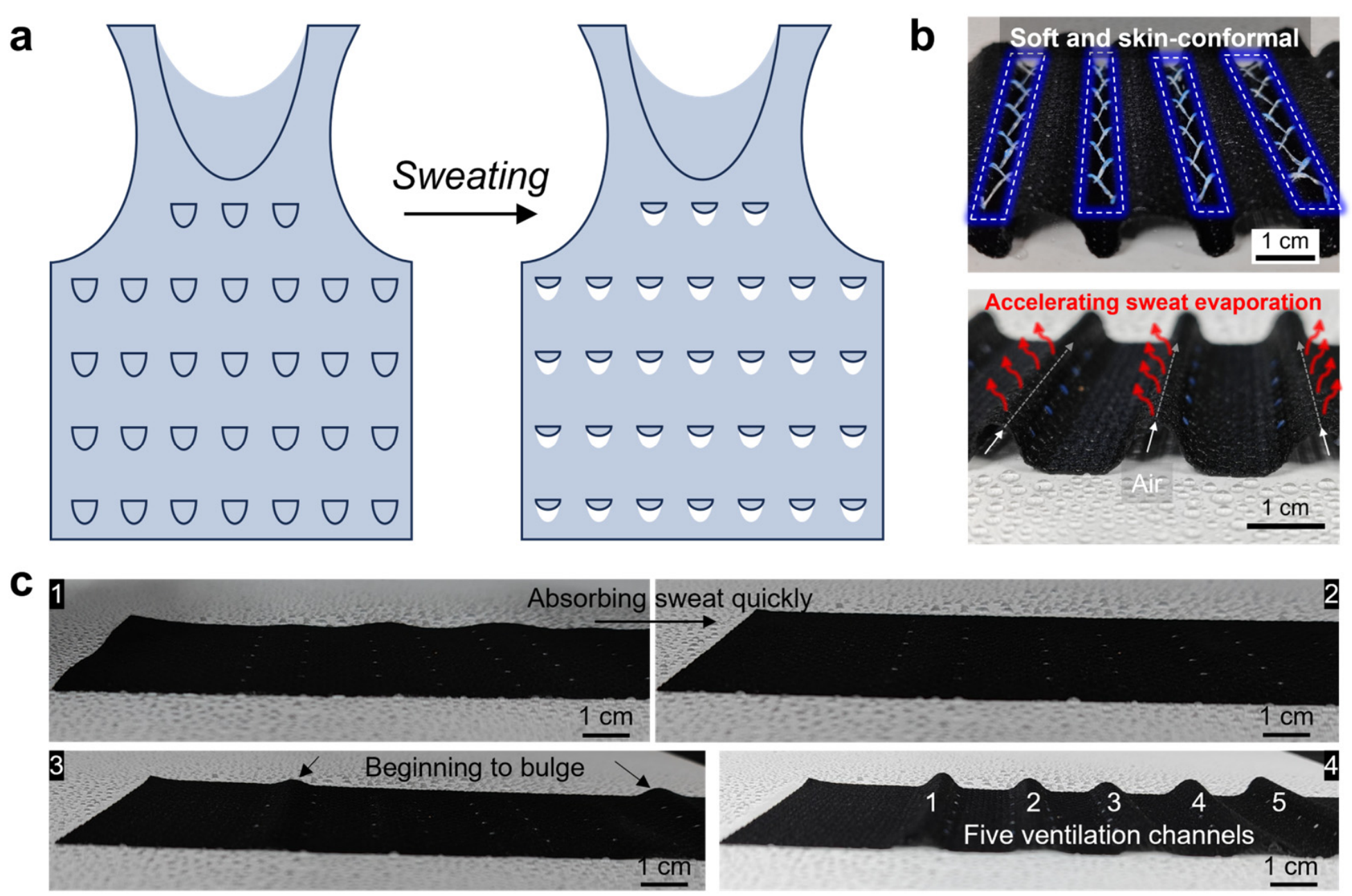

Fig. S46. Proof-of-concept demonstration of moisture-responsive fabric. (a) Schematic illustration: in

the dry state the fabric lies flat against the body, while upon wetting (e.g., by perspiration) the fabric forms localized ridges, opening ventilation channels between the fabric and the skin (*14*). **(b)** Photographs of the fabric with sewn-in HCHC fibers in the dry state (top) and after hydration (bottom), showing the formation of a corrugated structure. **(c)** Time-lapse images of the fabric placed on a wetted surface: (1) initial flat state, (2) after surface wetting, (3) onset of ridge formation, (4) fully developed corrugation with five ridges aligned with the embedded HCHC fibers. Contact with a pre-wetted surface was employed as the hydration method.

Rotational microengine

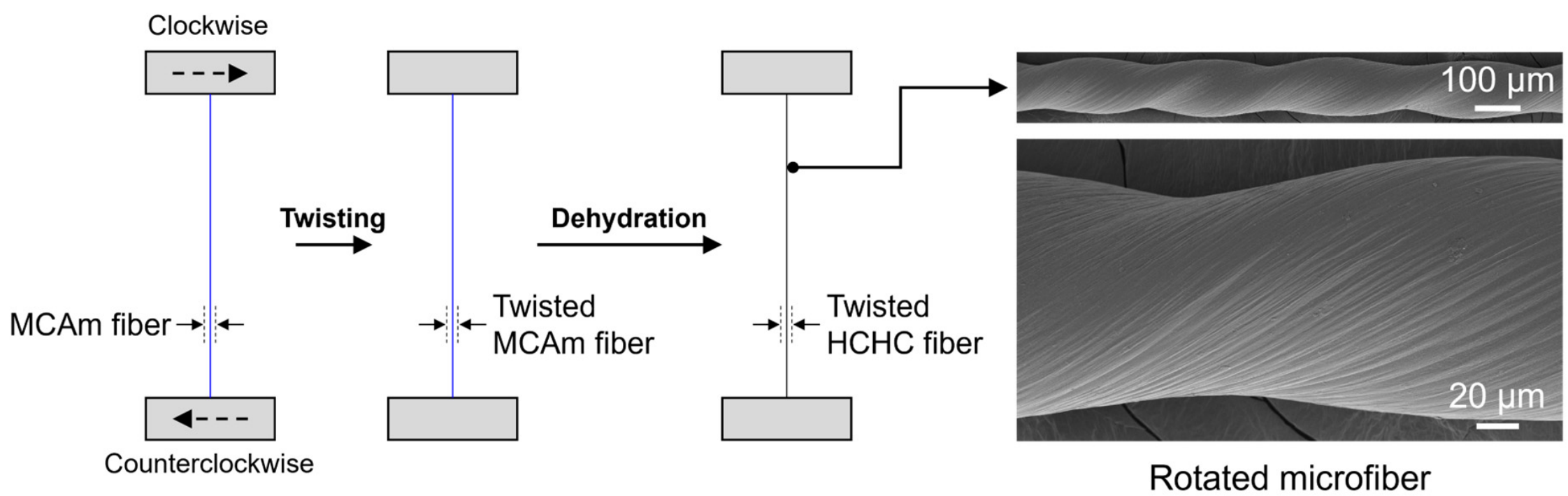

Fig. S47. Fabrication process and SEM image of rotational fiber based microengine. Representative SEM images from three independent measurements show similar results.

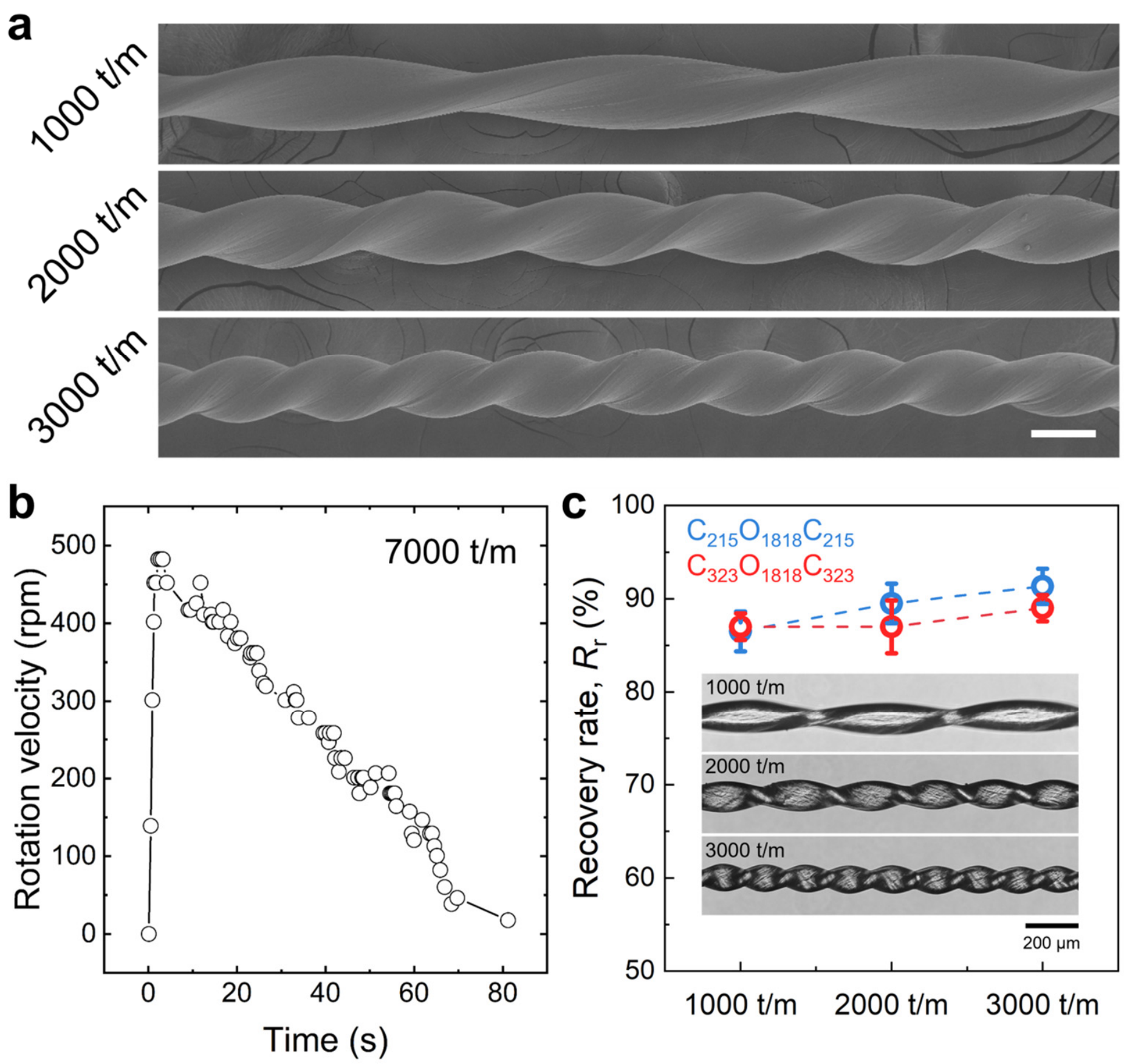

Fig. S48. (a) SEM images of the rotated microfibers show the twisted structure with twist of 1000, 2000, and 3000 turns per meter (t/m), respectively. Scale bar is 100 μm. Similar results were obtained in three independent experiments. **(b)** Rotation velocity of the microengine with twist of 7000 t/m under hydration stimulation. **(c)** Recovery rate R_r (the ratio of recovery number of rotations to the applied number of turns) of microengine with different hydrophobic block ratios and twisting degrees (*15*). Bath immersion was employed as the hydration method. Circles show mean values, error bars represent s.d. (n = 3 independent measurements). For the micrographs, similar results were obtained in three independent experiments.

10. Recyclability of MMC

To evaluate the recyclability of the MMC, used materials were recycled by lyophilizing and redissolving in THF. Rapid injection was used again to fabricate the material. Polymer molecular weight and distribution, mechanical performance and actuation performance of the recycled material were then evaluated and compared to original materials.

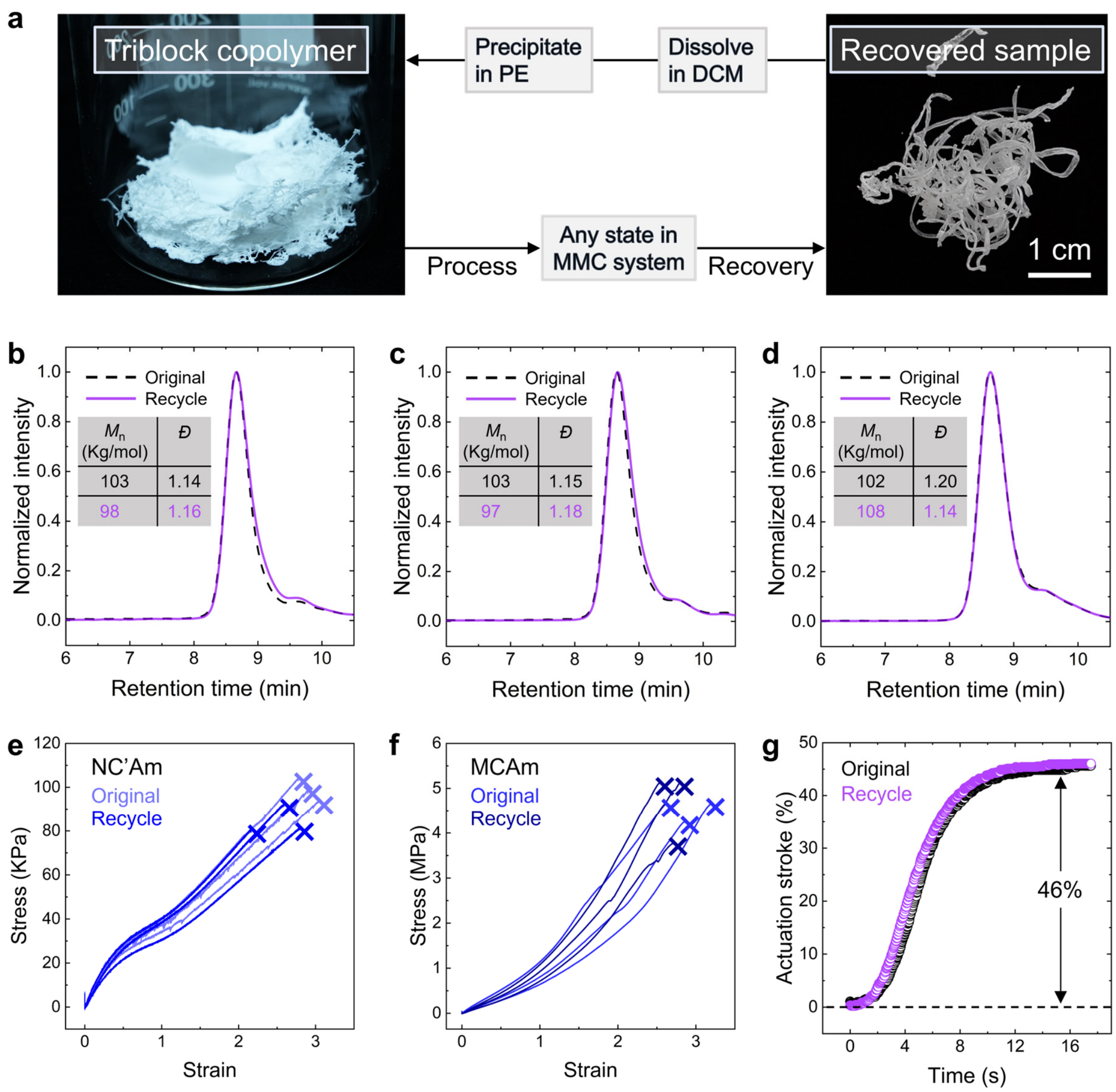

Fig. S49. (a) The material can be recycled back to raw triblock copolymer and reused. **(b–d)** SEC traces of original and recycled triblock copolymer. **(e, f)** Tensile stress-strain curves of original and recycled NC'Am and MCAm. **(g)** Actuation stroke-time curves of original and recycled HCHC.

11. Biocompatibility of MMC

The biocompatibility was assessed via hemolytic activity, CCK-8 assay, and Live/dead cells staining test.

Sterile defibrillated rabbit blood was obtained from Nanjing SenBeiJiaBiological Technology Co., Ltd. The obtained blood was firstly centrifuged (3000 rpm, 10 min) and washed with sterile PBS for 3 times to collect hemocyte, subsequently the collected blood cells were redispersed in sterile PBS to form blood cell suspension (5 wt%). Then, the blood cell suspension was mixed with sample and cultured at 37ºC for 2 h (0.1 mg material matrix, 0.1 mg polymer sample weight), followed by centrifugation to collect supernatant and optical density detection at 540 nm. Triton X-100 solution (0.2%, v/v) and PBS were selected as positive control and negative control, respectively.

To culture cells in a fiber-conditional medium, 1 mg fiber was incubated with 1 mL culture medium (DMEM) at 37ºC for 24 h to form the fiber-conditional medium. The fiber was sterilized via UV light before using. Normal human dermal fibroblast cells were seeded in a petri dish at a density of 1.5×10^4 cells per cm^2 and cultured for 2 days. Subsequently, the cell medium was replaced with fiber-conditional medium and pristine culture medium, followed by an additional 24 h incubation. Cells in pristine culture medium were used as controls. The cells were then stained using reagents in LIVE/DEAD® Viability/Cytotoxicity Kit for mammalian cells (Invitrogen™). Cell viability was assessed using a Fluorescence microscope (Leica DMIL LED), where live cells emitted bright green fluorescence (Ex/Em: ~495 nm/~515 nm), whereas dead cells emitted bright red fluorescence (Ex/Em: ~528 nm/~617 nm).

Hemolytic activity characterization

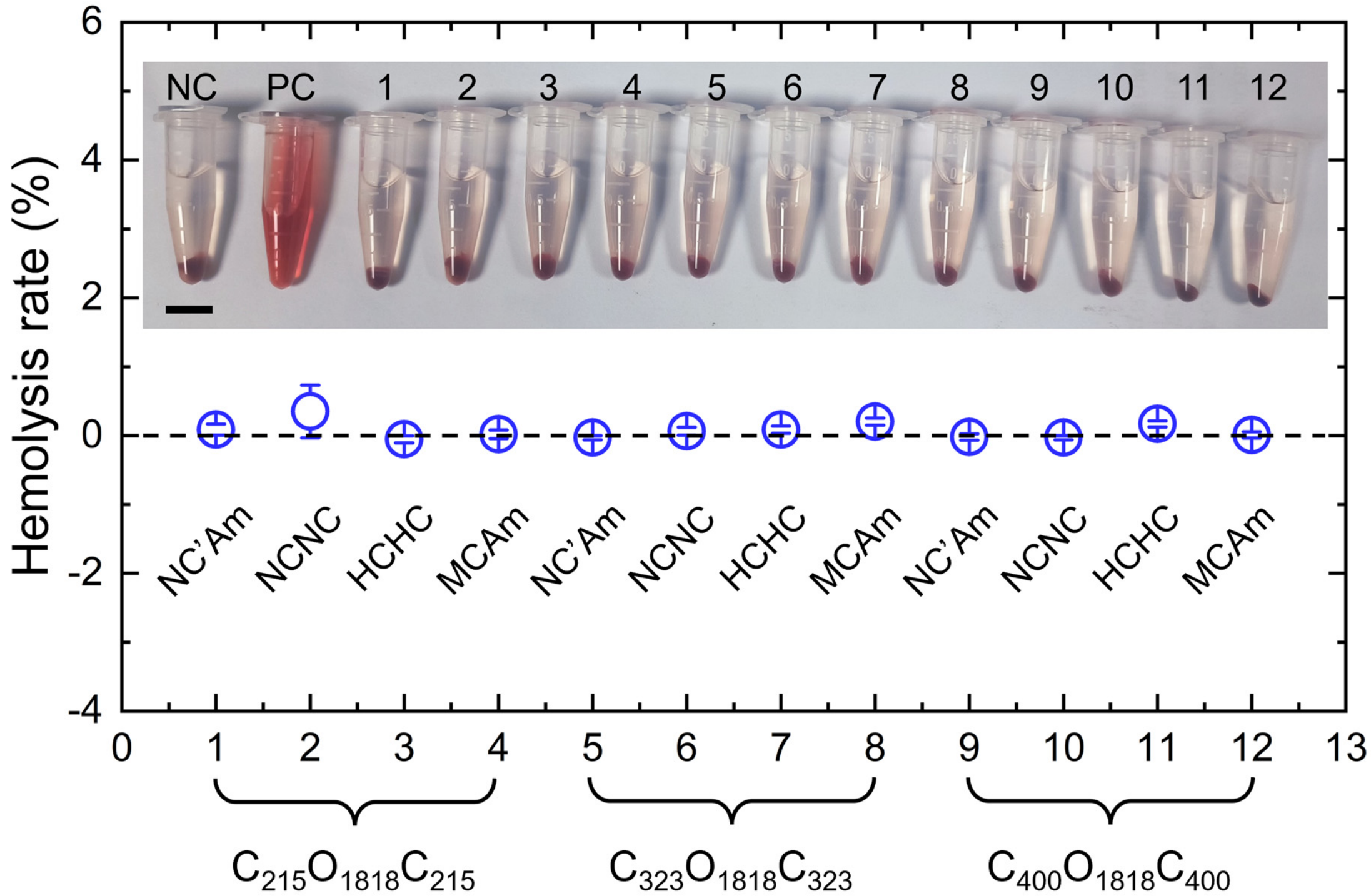

Fig. S50. The hemolysis rate of NC'Am, NCNC, HCHC and MCAm state were less than 0.5%. Scale bar is 1 cm. Circles show mean values, error bars represent s.d. (n = 3 independent measurements).

Cytotoxicity characterization

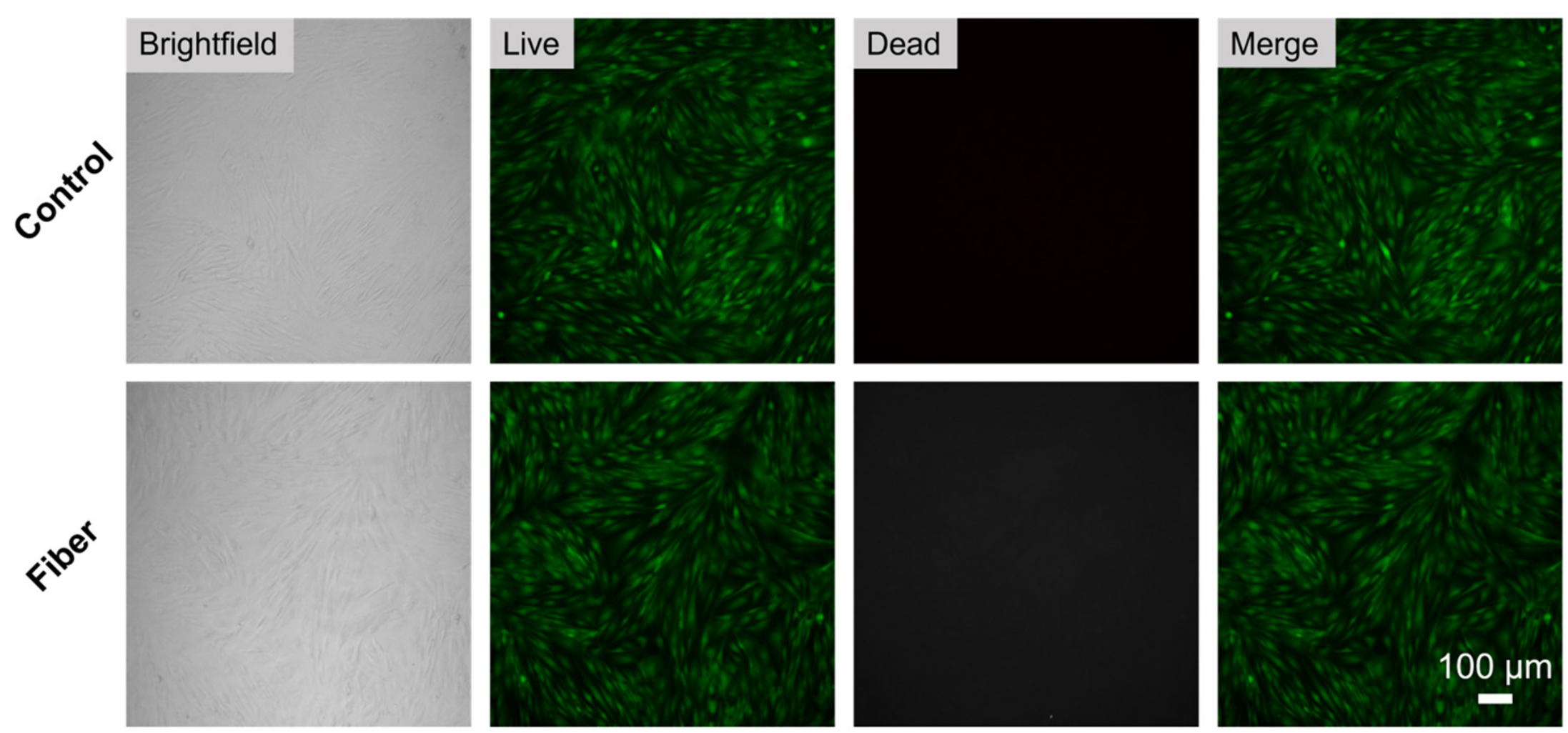

Fig. S51. In vitro cytotoxicity determined by live/dead cell staining. Human dermal fibroblast cells were

cultured with NC'Am state for 2 days and subjected to staining.

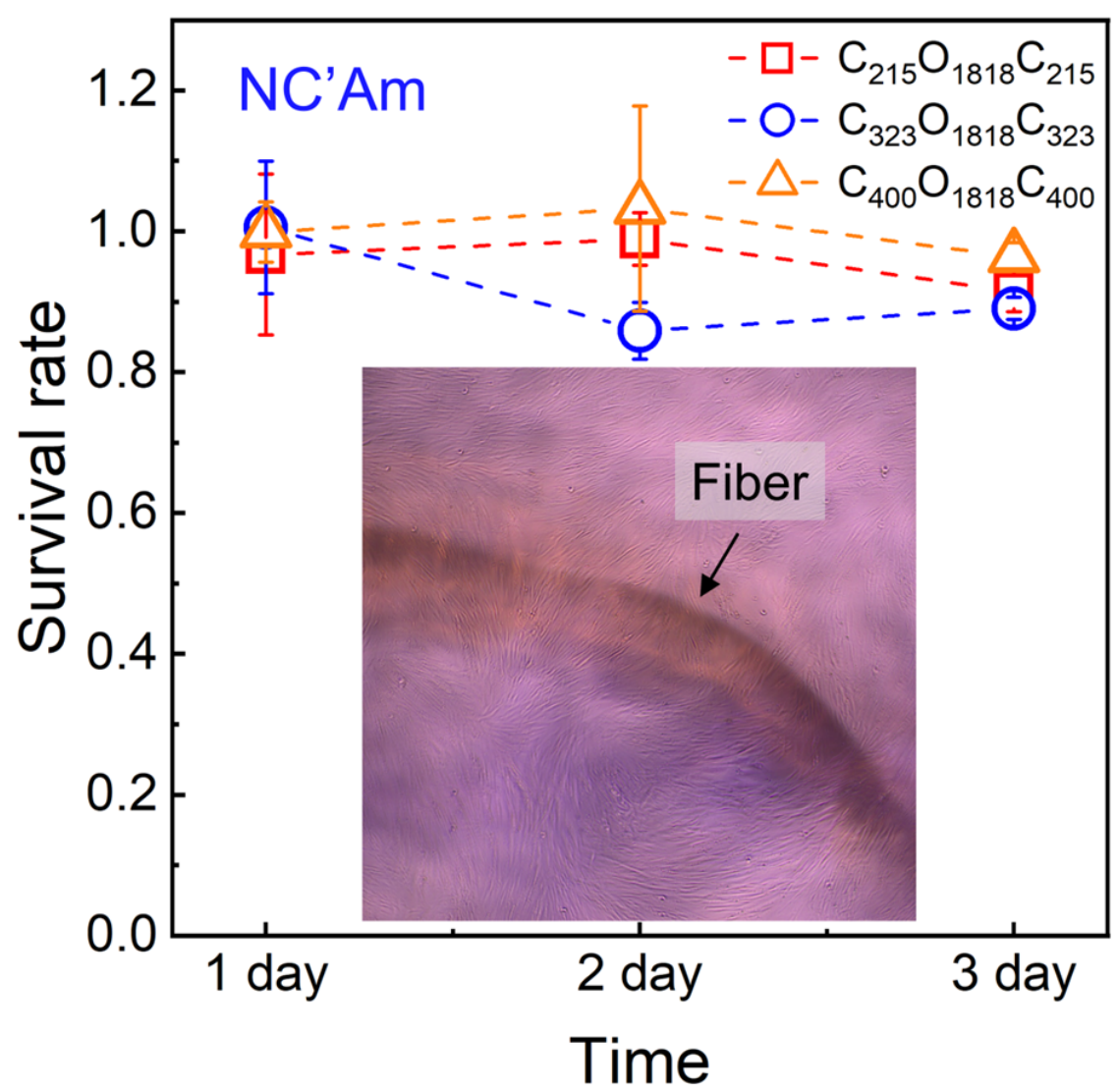

Fig. S52. In vitro cytotoxicity was determined by CCK-8 assay. Survival rate of normal human dermal fibroblast cells was determined after co-culturing with NC'Am state material for 1 day, 2 days and 3 days, respectively. Symbols show mean values, error bars represent s.d. (n = 3 independent measurements). For the micrograph, similar results were obtained in three independent experiments.

Supplementary References

1. C. Lang *et al.*, Solvent-non-solvent rapid-injection for preparing nanostructured materials from micelles to hydrogels. *Nat. Commun.*, 10:3855 (2019).
2. J. Yi *et al.*, Water-responsive supercontractile polymer films for bioelectronic interfaces. *Nature* **624**, 295-302 (2023).
3. J. R. Capadona, K. Shanmuganathan, D. J. Tyler, S. J. Rowan, C. Weder, Stimuli-Responsive Polymer Nanocomposites Inspired by the Sea Cucumber Dermis. *Science* **319**, 1370-1374 (2008).
4. L. Cera *et al.*, A bioinspired and hierarchically structured shape-memory material. *Nature Materials* **20**, 242-249 (2021).
5. T. Tian *et al.*, A body temperature and water-induced shape memory hydrogel with excellent mechanical properties. *Polymer Chemistry* **10**, 3488-3496 (2019).
6. F. Yang, A. Cholewinski, L. Yu, G. Rivers, B. Zhao, A hybrid material that reversibly switches between two stable solid states. *Nature Materials* **18**, 874-882 (2019).
7. M. Jorfi, M. N. Roberts, E. J. Foster, C. Weder, Physiologically Responsive, Mechanically Adaptive Bio-Nanocomposites for Biomedical Applications. *ACS Applied Materials & Interfaces* **5**, 1517-1526 (2013).
8. A. Balasubramanian, R. Morhard, C. J. Bettinger, Shape-Memory Microfluidics. *Advanced Functional Materials* **23**, 4832-4839 (2013).
9. I. M. Van Meerbeek *et al.*, Morphing Metal and Elastomer Bicontinuous Foams for Reversible Stiffness, Shape Memory, and Self-Healing Soft Machines. *Advanced Materials* **28**, 2801-2806 (2016).
10. W. Shan, T. Lu, C. Majidi, Soft-matter composites with electrically tunable elastic rigidity. *Smart Materials and Structures* **22**, (2013).
11. G. Chung *et al.*, Bioinspired, Rapidly Responsive Magnetically Tunable Stiffness Metamaterials. *Advanced Materials* **37**, (2025).
12. C. Lang *et al.*, Nanostructured block copolymer muscles. *Nature Nanotechnology* **17**, 752-758 (2022).
13. J. D. W. Madden *et al.*, Artificial muscle technology: physical principles and naval prospects. *IEEE Journal of Oceanic Engineering* **29**, 706-728 (2004).
14. W. Wang *et al.*, Harnessing the hygroscopic and biofluorescent behaviors of genetically tractable microbial cells to design biohybrid wearables. *Sci. Adv.* **3**, e1601984 (2017).
15. S. Utsumi *et al.*, Giant nanomechanical energy storage capacity in twisted single-walled carbon nanotube ropes. *Nature Nanotechnology* **19**, 1007-1015 (2024).
16. J. K. Percus, G. J. Yevick, Analysis of Classical Statistical Mechanics by Means of Collective Coordinates. *Physical Review* **110**, 1-13 (1958).
