# Supplementary material for: Block copolymer multistate material circuit
Source: Nat Commun. 2026 Oct 6;17:11393. doi: 10.1038/s41467-026-78278-2 (PMC13736946; doi:10.1038/s41467-026-78278-2)
Supplement: Supplementary file 4 [file 41467_2026_78278_MOESM2_ESM.pdf]

Description of Additional Supplementary Files

Supplementary Movie 1: Load-bearing behavior of an HCHC fiber. An HCHC fiber with a mass of 0.3 mg and a diameter of 76 μm supports a 300 g load, approximately one million times its own mass.

Supplementary Movie 2: Direct tensile comparison between an HCHC fiber and SUS304 stainless-steel wire. An HCHC fiber and a SUS304 stainless-steel wire of equal length and mass are pulled against each other until the stainless-steel wire fractures.

Supplementary Movie 3: Impact-induced structural rearrangement of MCHC. The movie shows the response of MCHC to impact loading, accompanied by structural rearrangement from MCHC to HCHC.

Supplementary Movie 4: Comparison of the impact responses of HCHC, HDPE, and Kevlar. The deformation and failure behaviors of the three materials are compared under identical impact-loading conditions.

Supplementary Movie 5: Heat-triggered rotational actuation of an HCHC-based microengine. Heating induces rotational actuation of the microengine and produces an on–off actuation response.

Supplementary Movie 6: Water-triggered actuation of HCHC fibers. Hydration dissolves the highly aligned B-block crystallites and converts HCHC into the soft hydrogel state MCAm, producing continuous actuation until the stored energy is released.

Supplementary Movie 7: Simulated wound closure driven by contraction of HCHC fibers. Upon contact with blood, the HCHC fibers contract and pull the wound edges together.

Supplementary Movie 8: Sweat-triggered opening of ventilation channels in a moisture-responsive fabric. Exposure to sweat causes the incorporated HCHC yarns to contract, opening channels between the fabric and skin and increasing local airflow.
